# A multi-mineral intervention to improve disease-related and mechanistic biomarkers in ulcerative colitis patients: Results from a randomized trial

**DOI:** 10.1371/journal.pone.0337408

**Published:** 2025-12-08

**Authors:** Muhammad N. Aslam, Danielle Kim Turgeon, Henry D. Appelman, Ryan Stidham, Shannon McClintock, Ron Allen, Gillian Moraga, Isabelle Harber, Kara J. Jencks, Molly M. McNeely, Ananda Sen, Karl J. Jepsen, James Varani

**Affiliations:** 1 Department of Pathology, University of Michigan Medical School, Ann Arbor, Michigan, United States of America; 2 Department of Internal Medicine, University of Michigan Medical School, Ann Arbor, Michigan, United States of America; 3 Division of Gastroenterology and Hepatology, Mayo Clinic, Rochester, Minnesota, United States of America; 4 Department of Family Medicine, University of Michigan Medical School, Ann Arbor, Michigan, United States of America; 5 Department of Biostatistics, University of Michigan Medical School, Ann Arbor, Michigan, United States of America; 6 Department of Orthopaedic Surgery, University of Michigan Medical School, Ann Arbor, Michigan, United States of America; Imam Abdulrahman Bin Faisal University, SAUDI ARABIA

## Abstract

**Introduction:**

The long-term goal of our ongoing studies is to determine if, and to what extent, a multi-mineral product (Aquamin) could benefit individuals with ulcerative colitis (UC). As a step toward achieving that goal, we carried out a pilot 180-day biomarker trial (clinicaltrials.gov ID: NCT03869905) in patients with UC in remission or with mild disease.

**Approach:**

A total of 28 subjects participated in the study. Each subject was randomly assigned to receive either Aquamin for 180 days or placebo for 90 days. At Day-90, placebo subjects crossed over to Aquamin for the final 90 days. At Day-0, -90 and -180, serum samples were analyzed for C-reactive protein (CRP), alkaline phosphatase (ALP), intestine-specific ALP (ALPI), and for biomarkers of bone turnover (osteocalcin, TRAP5b and bone-specific ALP [BALP]). Stool specimens were assessed for fecal calprotectin (fCAL) and colon biopsies were examined histologically by Geboes scoring at the same time points. Each subject underwent DEXA scanning (at Day-0 and Day-180 only). In addition, mass spectrometry-based proteomic assessment was performed using colon biopsies obtained at each time point.

**Results:**

Subjects who received Aquamin for the complete 180-day period (a total of 12) demonstrated improvements in all biomarkers (CRP, fCAL, ALP, ALPI, and Geboes scoring); this was not observed in the placebo group (16 subjects). When cumulative pre-post differences were compared between the Aquamin and placebo groups, Aquamin treatment significantly decreased these differences (a 24% decrease as compared to a 38% increase with placebo, p = 0.0284). Subjects who received Aquamin for 90-days showed intermediary responses. Subjects receiving Aquamin for 180 days also demonstrated increases in bone mineral density (BMD) and bone mineral content (BMC), resulting in a statistically significant increase (7.3%, p = 0.0324) in the hip strength index over the treatment period. This was accompanied by increases in osteocalcin and TRAP5b and a decrease in BALP. The proteomic screen demonstrated upregulation of multiple gut barrier proteins, cell surface transporter molecules and certain proteins with anti-inflammatory potential in response to Aquamin. Aquamin treatment also led to downregulation of several proteins associated with the pro-inflammatory state.

**Conclusion:**

The results presented here suggest that the use of a multi-mineral intervention improves disease-related biomarkers in patients with UC. These studies suggest the potential value of the mineral intervention as a low-cost, non-toxic adjuvant therapy for mild UC or for individuals with UC in remission.

## Introduction

Ulcerative colitis (UC) is a chronic disease manifested as diffuse mucosal inflammation along with superficial ulcer formation in the inner lining of the large intestine [1,2]. Large bowel injury may be focal in nature or widespread. Additional extra-colonic features may also exist; among the most prevalent are inflammation of the bile duct [3,4] and a propensity toward bone loss [5].

Dysregulation of the immune system [1,2] is thought to be the underlying pathophysiological mechanism, and the therapeutic armamentarium currently used for UC is aimed, primarily, at controlling inflammation [6]. Agents that target inflammation broadly including steroids and non-steroidal anti-inflammatory drugs such as mesalamine have long been treatment options [7]. More recently, biological agents (small molecules and antibodies) targeting specific components of the inflammatory process have become part of the treatment combination [8,9]. The use of biologics has dramatically expanded in recent years, but improved clinical outcomes continue to be approximately 20–30% over placebo in UC with mild-to-moderate disease [6,9], suggesting that additional aspects of UC pathophysiology need to be addressed.

Abnormal barrier function in the large bowel, leading to increased mucosal permeability, is another potentially important pathologic feature of UC [10–13]. The colonic barrier is designed to isolate the interstitium from colonic contents (e.g., bacterial cells and cell wall components, soluble bacterial toxins, pollens, other particulates, food allergens) while still providing for selective transport of water and essential minerals across the colonic wall. While inflammation, itself, is a cause of barrier dysfunction [11–13], pre-existing barrier defects provide a milieu in which inflammation can take hold [12]. While an intact barrier is essential to colonic health, there are no therapies that directly address barrier defects in UC [13,14] or in other inflammatory conditions of the bowel.

Our own past studies have shown that a marine red algae-derived multi-mineral product (Aquamin) can significantly improve gastrointestinal health in experimental animals when the mineral supplement is provided as a dietary component. In long-term (15–18 month) mouse studies, Aquamin inclusion in the diet significantly reduced the incidence of colon polyp formation [15,16] as well as tumors throughout the gastrointestinal tract [16] and in the liver [17]. A reduction in inflammation throughout the gastrointestinal tract and systemically was associated with these beneficial effects [15,16]. Additional studies by Aviello et al have demonstrated Aquamin’s ability to inhibit colitis in the IL10-/- mouse [18].

How a mineral supplement might function to produce gastrointestinal health benefits is not fully understood. In organoid culture studies carried out with human colon tissue from healthy subjects [19–21], or UC patients [21,22], as well as with the colon tissue exposed to a pro-inflammatory milieu [23,24], Aquamin treatment increased the elaboration of numerous proteins that contribute to barrier structure / function [19–24]. The same mineral supplement reduced the levels of several proteins that promote inflammation and increased the expression of proteins that counteract the pro-inflammatory response [19,22–24]. Subsequently, a biomarker trial in healthy human subjects revealed that many of the same protein changes seen in organoid culture were also seen in colonic biopsies after 90-days of daily Aquamin ingestion [25]. Based on the findings from preclinical studies and a pilot study in human subjects demonstrating that Aquamin can upregulate proteins involved in barrier integrity and modulate inflammation-related moieties, we hypothesize that Aquamin may have efficacy when used as adjunct treatment for individuals with UC or other chronic inflammatory bowel conditions where barrier integrity is compromised. As a way to begin testing this hypothesis, we conducted a pilot biomarker trial in subjects with UC in remission or with mild disease.

## Materials and methods

### Intervention

The interventional study was conducted with U.S. Food and Drug Administration (FDA) approval of Aquamin^®^ as an Investigational New Drug (IND# 141600). Aquamin^®^ is a calcium-, magnesium- and multi-trace element-rich product obtained from the calcified fronds of red marine algae of the *Lithothamnion* genus [26]. The calcium and magnesium ratio in Aquamin^®^ is approximately twelve to one (12:1); Aquamin^®^ also contains measurable levels of seventy-two additional trace elements. Aquamin^®^ is sold as a dietary supplement (GRAS 000028; Marigot Ltd., Cork, Ireland) and is used in various products for human consumption in Europe, Asia, Australia, and North America. A single batch of Aquamin-TG^®^ (Food Grade) was utilized for the trial presented here. It was provided in hydroxypropyl methylcellulose (HPMC) capsules, each formulated to contain 600 mg of Aquamin^®^ standardized to deliver 200 mg of calcium per capsule. Patients were prescribed four capsules of Aquamin^®^ daily (two in the morning and two in the evening), providing a total of 800 mg of calcium per day. Aquamin^®^ was administered orally in addition to their ongoing UC maintenance medications. S1 Table provides a complete list of elements detected in Aquamin and their relative daily intake amounts. Mineral composition of Aquamin-TG^®^ was established via an independent laboratory (Advanced Laboratories; Salt Lake City, Utah) using Inductively Coupled Plasma Optical Emission Spectrometry. Aquamin-TG^®^ has been used in our previous trial [25,27]. Maltodextrin (Nutrition Group PLC) was used as a placebo and was prescribed for oral administration in capsules that matched the active treatment in size, color, and weight (600 mg per capsule).

### Study design

#### Overall goal.

This single-site study was a pilot-phase, double-blind, randomized-controlled, one-way crossover interventional trial in which participants with UC, either in remission or with mild disease, were included. The overall goal of the study was to determine if taking Aquamin daily for 180 days could alter expression of several disease-related and mechanistic biomarkers.

### Regulatory oversight

This investigator-initiated interventional study was conducted with FDA approval, and with JV serving as the sponsor. Oversight was provided by the Institutional Review Board at the University of Michigan Medical School (IRBMED, HUM#156676). The Michigan Institute for Clinical and Health Research (MICHR) provided site monitoring, data collection, and data analysis tools (RedCap; Research Electronic Data Capture). A data and safety monitoring committee (DSMC) was responsible for reviewing the study every month to safeguard the welfare of study participants and ensure the study’s appropriateness and data integrity. The study was registered as an interventional clinical trial on clinicaltrials.gov (study identifier NCT03869905) in March 2019. The first subject was enrolled in October 2019, and the last subject completed participation in March 2023. All study participants provided written informed consent prior to enrollment and before starting any intervention. The trial protocol is available in S1 Text File, and the CONSORT (Consolidated Standards of Reporting Trials) checklist is presented in S2 Text File. This trial involving human participants was carried out in accordance with recognized ethical guidelines, for example, the Declaration of Helsinki, the International Ethical Guidelines for Biomedical Research Involving Human Subjects (CIOMS), the International Council for Harmonisation (ICH) Good Clinical Practice guidelines, the Belmont Report, and the U.S. Common Rule.

### Study participants

**Fig 1:**
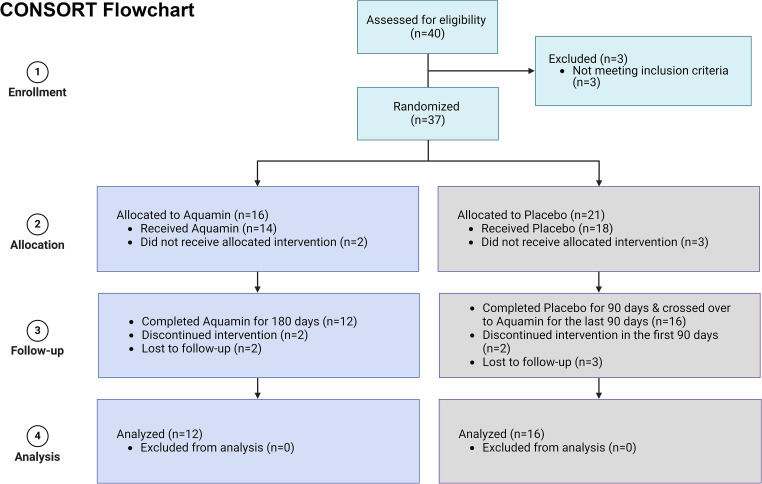
CONSORT Flow Diagram Depicting Randomization and Intervention Allocation. CONSORT flow diagram illustrates the process of participant randomization and allocation to intervention groups. The diagram shows the number of subjects assigned to each group, with an indication of those who did not receive the allocated intervention and were subsequently lost to follow-up in both groups. One study participant completed 90 days of participation in the placebo group and remained on Aquamin for the full 180-day period after crossing over due to the closure of the clinical research unit during the COVID-19 pandemic. As a result, this participant was analyzed in the study’s placebo (n = 16) and Aquamin 180-day (n = 13) groups except for proteomics.

The participants were recruited through the Michigan Medicine IBD (Inflammatory Bowel Disease) clinics and web portal (UMHealthResearch) and by posting flyers in the hospital. All study interactions and procedures were conducted in the Michigan Clinical Research Unit. Fig 1 presents a CONSORT flow diagram, detailing subject enrollment, randomization, and intervention allocation. Our initial intent was to include 40 subjects (20 per arm) in this pilot-phase study. While there was no prior clinical data on which to base this number, our earlier trial with healthy subjects had demonstrated that statistically significant differences in proteomic findings could be seen with as few as ten subjects per arm [25]. Based on the expected dropout rate, 20 subjects per group were assumed to be adequate. As it turned out, a total of 40 subjects were screened, and 37 subjects were enrolled in this interventional trial. Four subjects withdrew and five subjects were lost to follow-up. 28 subjects who started on treatment completed the study. This included 12 subjects who received Aquamin for the entire period of 180 days and 16 subjects who were randomized initially to placebo (Day-0 to Day-90) and crossed over to Aquamin for the final 90-days. Participants were males or non-pregnant females in generally good health, with a confirmed diagnosis of UC based on Mayo score [28] (which includes rectal bleeding, stool frequency, assessment by physician and endoscopic findings). Eligible individuals were either in remission or had mild disease at enrollment, as determined by the study gastroenterologist (DKT), with a stable maintenance therapy and a Mayo score of 5 or less. S2 Table presents the Disease Activity Index for Ulcerative Colitis (UCDAI), also known as Mayo score, at baseline, reflecting disease status prior to the start of the intervention. Exclusion criteria included individuals with severe acute UC, or individuals with Crohn’s disease, gastrointestinal/ colonic malignancy or gastrointestinal hemorrhagic disorders. Individuals with coagulopathy or receiving therapeutic doses of Coumadin or heparin were also excluded as were those with a history of kidney disease or kidney stones. A washout period of 30 days was required for certain medications, including supplements containing calcium or other minerals, vitamin D, fiber, antibiotics, steroids, and non-steroidal anti-inflammatory drugs except standard of care UC drugs (i.e., mesalamine). [Note: There were no deviations from the approved study protocol except for the following. One study participant completed the initial 90 days of participation immediately prior to the onset of the COVID-19 pandemic. The subject was scheduled to crossover to Aquamin for the last 90 days of the study in accordance with the study protocol, but due to the closure of the clinical research unit to make space for COVID-19 emergencies, remained on Aquamin for the complete 180-day period. This subject is included in both the placebo group for the first 90 days and as part of the Aquamin treatment group for the final phase.]

### Study protocol

**Fig 2:**
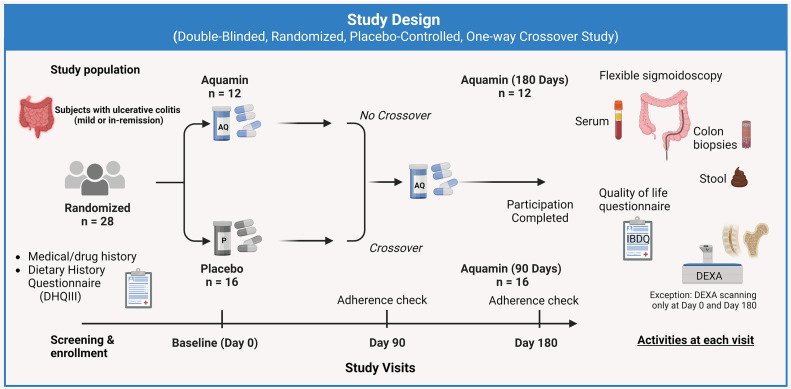
Schematic overview of study design. Study design is presented in graphical form. Subjects were randomized to either placebo or Aquamin. After 90 days of intervention subjects on placebo crossed over to Aquamin, while subjects on Aquamin remained on Aquamin treatment. There were, thus, three groups: i) Aquamin treatment for 180 days, ii) Placebo for 90 days and iii) Aquamin for 90 days.

Fig 2 summarizes the study design of this trial. Each subject had four study visits – i.e., i) screening and enrollment; ii) baseline (Day-0), iii) midpoint (Day-90) and iv) final visit (Day-180). Briefly, at the screening visit, individuals who chose to participate after having the study explained were given the NIH Diet History Questionnaire III (DHQ III) [29], a food frequency questionnaire that includes portion size and dietary supplement questions, as a way to evaluate baseline calcium intake levels [25,27]. Participants were also asked about use of dietary supplements, antibiotics, and non-steroidal anti-inflammatory drugs. A brief drug and medical history was taken and a brief physical examination was performed. S3 Table provides the therapeutic treatment log and maintenance medications used by study participants at the time of study initiation. It also provides disease status at the start of the participation (including pre-participation colonoscopy and pathology report) with year of diagnosis and location and extent of UC lesions. Incidence of the last flare was documented. Eligible subjects signed informed consent at the screening visit prior to any study participation.

At the baseline visit, participants underwent flexible sigmoidoscopy (unprepped; *i.e.,* without bowel cleansing procedure). Twelve 2.8 mm colonic biopsies were obtained along with five stool specimens from within the sigmoid colon (20 cm above the anus) using cold Captura Pro biopsy forceps (Cook Medical). If there was evidence of an active UC lesion, the endoscopist avoided these areas while taking biopsies. Tissue samples were saved in 10% formalin for histology or snap-frozen in liquid nitrogen and saved at −80^o^C for proteomic analysis. Stool samples were either transported in liquid nitrogen and stored at −80^o^C or transported on ice and processed for fecal calprotectin (fCAL) assessment using extraction buffer (BÜHLMANN) and then stored at −20^o^C. Venous blood samples were obtained.

After baseline sigmoidoscopy, participants were randomized to either placebo or Aquamin (providing 800 mg of calcium per day). Participants continued with their ongoing maintenance therapy for UC. During the interventional period, participants were contacted by study coordinators monthly to assess study progress and adherence to the study protocol as well as to identify unwanted side effects. Compliance was assessed by capsule log entries and by counting unused capsules returned at the end of each 90-day period.

At the end of the initial 90-day intervention period (90 ± 5 days), participants again underwent unprepped flexible sigmoidoscopy and ten colonic biopsies along with five stool specimens were collected and stored as at baseline. Blood was also taken for serum markers as at baseline. After the 90-day visit, subjects on Aquamin remained on Aquamin, while subjects on placebo switched over to Aquamin for the final 90 days. At the end of 180-day interventional period, participants underwent the same procedure as at baseline and midpoint and completed all the tasks including capsule count to confirm compliance. [Note: the additional biopsies and stool specimens collected at each timepoint were retained for backup or saved for future use in immunohistochemical, microbial and metabolic analyses. At the baseline visit and at the Day-90 and Day-180 visits, multiple clinical and research endpoints were evaluated as described in the following sections.]

### Serum biomarkers

This included a comprehensive metabolic panel in which total albumin, bilirubin, aspartate aminotransferase (AST), alanine aminotransferase (ALT), and alkaline phosphatase (ALP) were assessed along with additional moieties designed to assess liver and kidney function. C-reactive protein (CRP) levels were assessed along with the metabolic panel. The Michigan Medicine laboratory processed these serum samples and generated individual reports for each participant according to their standard operating procedure. Additional serum samples were used to evaluate the intestine-specific form of alkaline phosphatase (ALPI) by ELISA (AssayGenie), and other serum endpoints related to bone health (see below). These were assessed as research endpoints. The comprehensive metabolic panel was evaluated primarily as part of a safety study although CRP and ALP values are routinely assessed as part of UC management [30,31].

### Fecal calprotectin

Stool samples collected at each visit (Day-0, Day-90 and Day-180) were assessed for fCAL at the end of the study by ELISA (BÜHLMANN). fCAL is a major cytosolic protein of neutrophils and its level in stool specimens is a reliable, quantitative, non-invasive measure of mucosal inflammation in UC [32,33]. Previous studies have demonstrated a change in fCAL level corresponding to altered disease activity status [32,33]. Fresh stool samples were processed after each visit using the extraction buffer (B-CAL-EX by BÜHLMANN) to extract calprotectin and saved at −20^o^C. ELISAs were conducted at the end of the study to avoid batch-to-batch variability.

### Histological evaluation of colonic biopsies

After formalin-fixation and paraffin-embedding, colon biopsies were sectioned and stained with hematoxylin and eosin (H&E) to assess structural features in the colonic wall and mucosal inflammation. The assessment was performed in a blinded fashion by the GI histopathologist (HDA) using a simplified Geboes Scoring System; this provides a validated semi-quantitative assessment of the histological findings of colon affected by UC [34]. It consists of five grades, ranging from grade 0 (No abnormality) to grade 4 (Ulcer formation). Grades differentiate between quiescent, mildly active, and moderate to severely active disease levels. Higher Geboes scores also provide indication of the severity of mucosal inflammation.

### Symptomatic and quality of life UC assessments

A clinical instrument and quality of life survey – i.e., Inflammatory Bowel Disease Questionnaire (IBDQ) – was used to longitudinally monitor subjects’ UC-related symptoms over the course of the study [35]. It is a 32-item questionnaire that captures disease parameters on four domains of functioning and well-being: bowel and systemic symptoms, and emotional and social functions [35]. The total IBDQ score ranges from 32 to 224 and is generated by tallying up all 32 items. IBDQ was administered on each study visit. Higher IBDQ scores represent improved quality of life. Patients were also asked to share their experience in their own words at the completion of the study or at the close-out call two weeks later.

### Dual-energy X-ray absorptiometry (DEXA) scanning and serum bone biomarkers

DEXA scanning was performed at baseline (Day 0) and the final visit (Day 180) but was not done at the Day-90 visit with the anticipation that 90 days of intervention would not be long enough to detect differences and would not justify exposure of subjects to the additional radiation. DEXA scanning was performed using a GE Lunar Prodigy ADVANCE Plus (enCORE-based X-ray Bone Densitometer). DEXA outputs provided bone mineral density (BMD), bone mineral content (BMC) and area values for upper femoral shaft including femoral neck, and lumbar spine. These DEXA outputs along with additional variables (buckling ratio, cross-sectional area or moment of inertia and additional geometric parameters) along with variables such as age and gender were used by the software associated with the DEXA scan to calculate a hip strength index as described by Yoshikawa et al [36].

Serum bone turnover markers (Osteocalcin, tartrate-resistant acid phosphatase 5b [TRAP5b] and bone-specific alkaline phosphatase [BALP]) were assessed by ELISAs (AssayGenie) at all three study visits (i.e., baseline, Day-90 and Day-180).

### Proteomic assessment of tissue biopsies – Data-Independent Acquisition

Proteomic assessment of the colon mucosal biopsies collected at each study visit was conducted at the IDeA National Resource for Quantitative Proteomics (Little Rock, AR) using the Data-Independent Acquisition (DIA) approach. A complete description of the technical details for proteomic data acquisition is presented as S3 Text File.

Following data acquisition, data were searched using an empirically corrected library against the UniProt Homo sapiens database (April 2022) and a quantitative analysis was performed to obtain a comprehensive proteomic profile. Proteins were identified and quantified using EncyclopeDIA [37] and visualized with Scaffold DIA using 1% false discovery thresholds at both the protein and peptide levels. Protein MS2 exclusive intensity values were assessed for quality using ProteiNorm [38]. The data were normalized using VSN [39] and analyzed using proteoDA to perform statistical analysis using Linear Models for Microarray Data (limma) with empirical Bayes (eBayes) smoothing to the standard errors [40]. The abundance ratio (Log_2_ fold-change) was calculated by comparing baseline Log_2_ VSN normalized intensities to the post-intervention intensities. The current study’s proteomic analysis involved a directed search of proteins involved in differentiation, barrier-related cell adhesion proteins, ion transporters and pro- or anti-inflammatory molecules. (Note: The complete set of unbiased findings from the colonic biopsies will be fully described in a separate report. Additionally, a parallel set of proteomic data obtained from serum samples taken at the same time points and analyzed in the same manner will be included). QIAGEN Ingenuity Pathway Analysis (IPA) was used to find significantly altered pathways by the participating proteins. IPA also provided predictions on the activated or inhibited status of these pathways. This commercial software is based on a knowledge database which can identify specific pathways, and it can generate biological networks influenced by a given set of proteins and their observed expression. These proteomics data are deposited to Mass Spectrometry Interactive Virtual Environment–MassIVE (massive.ucsd.edu), a data repository for open access, [Identification ID is MassIVE MSV000095472 and ProteomeXchange accession number is PXD054344].

### Statistical evaluation

This pilot study was designed to assess feasibility and tolerability, as well as to evaluate both disease- and research-related biomarkers. We anticipated that the study would demonstrate trends useful for generating effect size estimates and formulating a research hypothesis for a future fully powered, large-scale trial. It was anticipated that Aquamin would have a positive effect on specific biomarkers, for some of which the effect would be more prominent than the others.

Based on the study design, pre-intervention and post-intervention values were obtained from each subject for the various serum analytes in the metabolic panel, as well as for CRP, fCAL, ALPI, histological scores, bone DEXA markers and each ELISA marker. Group means and standard deviations were generated for individual endpoints and the pre-post data were analyzed by paired t-test to calculate two-tailed p values using 95% confidence level. Then pre-post-intervention values from the Day-180 Aquamin-treatment group and pre-post data from the placebo group were compared. Group means were used to calculate a composite value for both groups and analyzed for significance by a two-tailed unpaired t-test. Similarly, the means of serum bone markers (Osteocalcin, TRAP5b and BALP) were combined to obtain a composite score for two-group analysis and significance was assessed by two-tailed unpaired t-test. GraphPad Prism v10.2 was used for these analyses.

Proteomic data were generated by the DIA approach, and the limma software package was used to generate p-values reflective of differences between the means of the two groups (comparator being the respective pre-intervention value). The unadjusted p-values were corrected using Benjamini-Hochberg method (also known as the False Discovery Rate) in limma. For pathways enrichment analysis, IPA uses the Fisher’s Exact Test to calculate a statistical significance (p-value) of overlap of the dataset molecules with various sets of molecules that represent annotations such as Canonical Pathways. A p-value <0.05 was considered significant. IPA calculates the Z-score by comparing observed expression to the expected expression in the knowledgebase, predicting up or downregulation and weighted by the underlying findings. A Z-score of ≥ 2 or ≤ −2 is considered significant.

Due to the small sample size, analyses were not adjusted for baseline sociodemographic data including gender, or for existing treatments or baseline dietary calcium intake amounts.

## Results

### Participant characteristics

Twenty-eight subjects completed the study. This included 12 subjects who received Aquamin for 180 days and 16 subjects who were randomized initially to placebo (Day-0 to Day-90) and crossed over to Aquamin for the final 90-days (Figs 1 and 2). Demographic profiles including ethnicity, gender and age are presented in S4 Table. Calcium-intake levels based on the DHQ3 survey recorded at the start of the study and body mass index (BMI) values are also provided in S4 Table.

S5 Table provides a list of intervention-related adverse events (AEs). There were no serious AEs reported, and AEs related to gastrointestinal symptoms were similarly distributed between the two cohorts: 29 AEs with placebo and 28 AEs with Aquamin. This is consistent with our previous findings in healthy subjects [25,27] and suggests that safety or tolerability will not be an issue with Aquamin use for 180 days in UC patients.

### Serum chemistry (metabolic panel) values

A panel of serum chemistry values from the comprehensive metabolic panel was obtained for each subject at the baseline visit and at Day-90 and Day-180 visits. The chemistry markers were assessed primarily as part of a safety evaluation but were also used to identify any potentially beneficial response to Aquamin. Results of these studies are presented in Table 1. With the exception of ALP (see below), there was little difference in pre- and post-treatment values for the metabolic panel analytes in either treatment group and most of the values remained within the normal reference range of the tests (Table 1).

**Table 1:** Serum chemistry (metabolic panel) values at the baseline, Day-90 and Day-180.

Group		Total protein	Albumin	AST	ALT	ALP	Bilirubin	Calcium
		*(6.0–8.3 g/dL)*	*(3.5–5.0 g/dL)*	*(M:14–20 F:10–36 U/L)*	*(M:10–40 F:7–35 U/L)*	*(25–100 U/L)*	*(0.0–1.4 mg/dL)*	*(8.8–10.4 mg/dL)*
**Placebo**	**Pre**	7.20 ± 0.45	4.63 ± 0.24	24.50 ± 5.09	22.31 ± 8.01	78.81 ± 24.89	0.71 ± 0.25	9.50 ± 0.26
	**Post**	7.11 ± 0.38	4.57 ± 0.19	22.50 ± 3.81	19.63 ± 5.74	80.69 ± 22.66	0.67 ± 0.34	9.46 ± 0.22
**Aquamin Day-90**	**Pre**	7.12 ± 0.43	4.53 ± 0.22	24.41 ± 5.64	21.63 ± 9.39	76.85 ± 17.48	0.68 ± 0.34	9.51 ± 0.28
	**Post**	7.10 ± 0.43	4.53 ± 0.25	24.48 ± 6.60	21.70 ± 12.53	74.33 ± 20.29	0.68 ± 0.30	9.61 ± 0.35
**Aquamin Day-180**	**Pre**	7.16 ± 0.48	4.46 ± 0.24	26.08 ± 6.79	23.92 ± 11.83	79.00 ± 18.61	0.68 ± 0.34	9.60 ± 0.33
	**Post**	7.10 ± 0.59	4.47 ± 0.29	25.38 ± 5.58	21.62 ± 8.83	71.85 ± 15.63*	0.68 ± 0.35	9.64 ± 0.45
**Group**		**Sodium**	**Potassium**	**Chloride**	**Carbon Dioxide**	**BUN**	**Creatinine**	**Glucose**
		***(136–145 mmol/L)***	***(3.5–5.2 mmol/L)***	***(96–106 mmol/L)***	***(23–30 mmol/L)***	***(10–20 mg/dL)***	***(M:0.6–1.2 F:0.5–1.1 mg/dL)***	***(<100 mg/dL)***
**Placebo**	**Pre**	138.88 ± 1.78	4.30 ± 0.29	103.88 ± 3.03	28.25 ± 2.11	13.75 ± 4.57	0.83 ± 0.18	88.69 ± 16.86
	**Post**	139.19 ± 1.64	4.27 ± 0.25	104.69 ± 1.96	28.38 ± 1.96	12.56 ± 3.29	0.81 ± 0.16	88.75 ± 18.70
**Aquamin Day-90**	**Pre**	139.67 ± 1.86	4.26 ± 0.29	105.26 ± 1.87	27.52 ± 2.17	13.15 ± 3.21	0.84 ± 0.16	88.63 ± 16.55
	**Post**	139.48 ± 1.74	4.24 ± 0.24	104.63 ± 2.10	28.26 ± 1.97	14.81 ± 4.01	0.87 ± 0.15	90.59 ± 12.39
**Aquamin Day-180**	**Pre**	140.08 ± 1.98	4.32 ± 0.36	105.23 ± 2.49	26.85 ± 2.27	13.69 ± 2.98	0.87 ± 0.17	87.69 ± 12.72
	**Post**	139.15 ± 1.34	4.35 ± 0.30	104.31 ± 2.59	27.85 ± 1.52	15.00 ± 4.45	0.88 ± 0.19	85.77 ± 8.36

A comprehensive serum metabolic panel was conducted for each participant at the Michigan Medicine Pathology and Clinical Laboratory at baseline, Day-90, and Day-180 as part of the safety assessment. The normal range (reference range) for each analyte is given at the top of the panel. Values represent the mean and standard deviation for each analyte, calculated by analyzing the data from each subject at each timepoint. An asterisk (*) in the Aquamin^®^ Day-180 sample for ALP indicates p < 0.05 (pre-post, paired, two-tailed). For AST, ALT, and Creatinine, reference ranges are different for males and females; however, due to small sample size, these have been merged to provide single mean value for each analyte.

AST: Aspartate aminotransferase; ALT: Alanine aminotransferase; ALP: Alkaline phosphatase; BUN: Blood Urea Nitrogen.

### Total serum alkaline phosphatase

**Fig 3:**
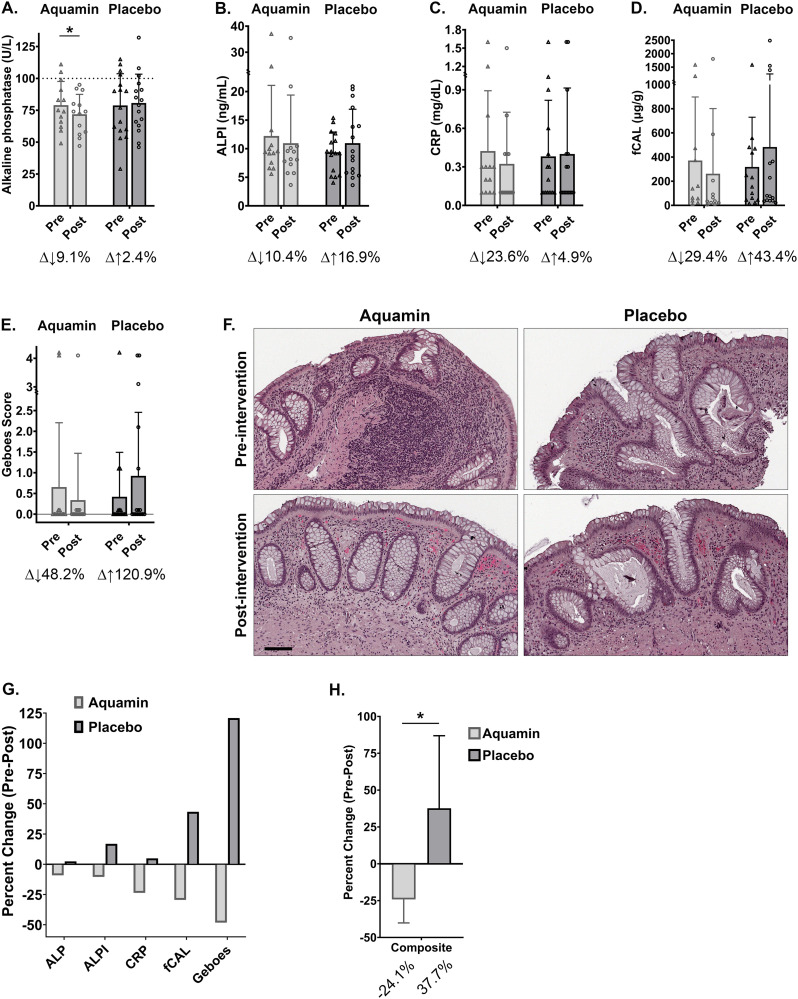
Chemistry and histology endpoints: Aquamin (180-day treatment group) versus placebo. A. Serum alkaline phosphatase (ALP): Values (U/L) determined as part of the serum chemistry (metabolic) panel. B. Serum intestine-specific alkaline phosphatase (ALPI): Values (ng/mL) determined by ELISA. C. Serum C-reactive protein (CRP): Values (mg/dL) determined in serum. D. Fecal calprotectin (fCAL): Values (µg/g) determined by ELISA (BÜHLMANN) in stool samples. E. Histological assessment of colon biopsies: Values presented as a simplified Geboes score. All values represent treatment group means and standard deviations. Delta values shown beneath each pair of bars indicate percentage change in the post-treatment value relative to the pretreatment value. Statistical significance between pre- and post-treatment values was determined by paired t-test; asterisk (*) indicates p < 0.05. F. Representative histological appearance of colon tissue from one subject in each treatment group, assessed at Day 0 (pre-intervention) and at the end of treatment (post-intervention). Pre-intervention colon biopsies from both subjects show similar histological features of UC, including crypt architectural distortion and infiltration of pro-inflammatory cells. Post-intervention biopsies from the placebo group continued to display these abnormalities, whereas notable improvement was observed in the subject treated with Aquamin. G. Aquamin versus placebo comparison: Pre- and post-treatment differences for each endpoint are plotted for the two groups to show the divergence between them. H. A composite score was generated by combining the five individual endpoint scores. Statistical significance (p < 0.05) between group (composite) values was determined by unpaired t-test.

Among the entire panel of serum markers assessed, only one marker measurably changed over the course of treatment; this was ALP, which decreased by 9.1% (i.e., from 79 ± 19 U/L to 72 ± 16 U/L) in the group of individuals receiving Aquamin for the entire 180-day period (p = 0.0046 by paired t-test) (Fig 3A). By comparison, the average ALP level increased by 2.4% in the placebo group (Fig 3A). When placebo and Aquamin groups were compared by two-sample t-test comparing independent means applied to the pre-post differences for ALP, the p-value was equal to 0.07.

When serum ALP data were examined on a subject-by-subject basis, we identified eight individuals whose ALP values were above the 100 U/L level that is the upper limit of normal range (The reference range for normal ALP at the Michigan Medicine Laboratory is 25–100 U/L). Of the eight individuals, three were from the cohort that was randomly assigned to receive Aquamin for 180 days, while the other five were in the placebo group. At the end of the treatment period, there was an average decrease of 12% among the individuals treated with Aquamin (−17%, −10% and −8% compared to pretreatment values; p < 0.06 by paired t-test). In contrast, there was virtually no change in the average serum ALP level among the five subjects in the placebo group (19%, 20%, 4%, −7% and −26% compared to pretreatment values).

### Intestine-specific alkaline phosphatase

Serum ALP activity reflects contributions from multiple tissue sources of enzyme [41]. As a follow-up, levels of intestine-specific ALP (ALPI) were assessed by ELISA. It can be seen in Fig 3B that the intestinal isoform of ALP decreased by 10.4% in subjects receiving Aquamin over the 180-day treatment period. In contrast, ALPI increased among the 16 individuals in the placebo group by an average of 16.9% (Fig 3B).

When ALPI levels were compared among the 8 subjects with elevated total ALP, there was an average 27% decrease among the three Aquamin-treated subjects (−17%, −23% and −40%, respectively; p < 0.05 by paired t-test) while the average ALPI level increased by 26% among the five subjects receiving placebo only (13%, 26%, 43%, 48% and −14% change from pretreatment). This difference in ALPI between the Aquamin-treated and placebo groups was statistically significant (p = 0.018 by unpaired t-test).

### Serum C-reactive protein

CRP values are presented in Fig 3C. Prior to initiation of treatment, CRP levels were above baseline in 16 specimens (out of 28 total). Average CRP values were similar between the placebo and Aquamin-treatment groups (0.38 mg/dL and 0.42 mg/dL, respectively). Over the course of treatment, there was a 23.6% decline in CRP values among subjects receiving Aquamin (group average = 0.32 mg/dL) and a 4.9% increase in the placebo group (average = 0.40 mg/dL) (Fig 3C).

### Fecal calprotectin

fCAL levels were assessed in stool specimens collected from each subject prior to initiation of treatment and at the Day-90 and Day-180 visits. In parallel, colon biopsies obtained at each timepoint were examined histologically using the criteria described in the Materials and Methods Section. As can be seen from Fig 3D, pretreatment fCAL values were similar between the Aquamin treatment group and the placebo group (371 µg/gram versus 320 µg/gram). This included 8 of 14 placebo group values above the ≥ 160 μg/gram level that is considered a mark of ongoing inflammation [42] and 5 of 11 values in the 180-day Aquamin treatment group. At the end of treatment period, there was a 29.4% average decrease in fCAL values in the Aquamin treatment group while a 43.4% increase was seen in the placebo group. Among individuals receiving Aquamin for the 180-day period, 3 of 11 still had fCAL values ≥160 μg/gram at the end of treatment period while 6 of 14 subjects in the placebo group were still above this “cutoff” level (Fig 3D).

### Histological evaluation of colon biopsies

Blinded histological examination of H&E-stained sections of colonic biopsies was performed in parallel with fCAL measurements. Histological grading utilized simplified Geboes scoring (Fig 3E). The majority of pretreatment tissue sections were histologically normal or demonstrated only architectural evidence of past injury (abnormal crypt size and shape) (Fig 3F). Evidence of an inflammatory infiltrate was observed in five specimens (out of 28), and in three of these specimens, the inflammatory cells (mostly neutrophils) were present in the epithelium as well as in the lamina propria. Epithelial damage accompanying the inflammatory cell infiltration was observed in two of the three specimens. Given the lack of evidence for active disease in most specimens, the average group scores were low (less than 1.0) to begin with. The group score increased among placebo recipients over the 90-day treatment period (121% increase), due primarily to the presence of the inflammatory infiltrate seen in four post-intervention specimens. In contrast, in the group of individuals receiving Aquamin for 180 days, the score decreased by 48%; this was due largely to reduced inflammatory cell infiltrate seen in the biopsy specimen from one of the two individuals with a high score at initiation. Fig 3F provides examples of pre- and post-treatment histology in a Day-180 Aquamin-treatment group specimen and in a placebo group specimen.

Fig 3G summarizes the pre- and post-treatment findings for each parameter, comparing results from the Aquamin-treatment group to placebo. A composite index made up of the five individual parameters presenting pre-post differences for Aquamin intervention and placebo in Fig 3G is shown in Fig 3H. The divergence in outcomes between the Aquamin-treatment and placebo groups is striking (p = 0.0284 by unpaired t-test).

### Summary of findings after 90-days of Aquamin-treatment

At the end of the intervention period, a total of 28 subjects had received Aquamin for at least 90-days (i.e., 12 subjects from the 180-day Aquamin treatment group at the intermediate timepoint and 16 subjects from the placebo group that crossed over to Aquamin on Day-90). To summarize, trends were similar to what was observed with Aquamin over the 180-day period but less definitive. For example, we observed a 3.3% decline in serum ALP, a 16% increase in ALPI and a 10.3% decline in CRP after 90 days of treatment with Aquamin. At the same time, there was an 18.9% increase in fCAL (compared to 43% increase in placebo). Histological analysis of colon specimens after 90 days of treatment revealed a 41% decrease in the simplified Geboes score as compared to 121% increase with placebo. Day-90 values for the entire group of subjects are presented in S1 Fig.

### Subject perceptions (IBDQ scores and subject self-reporting)

The IBDQ questionnaire was employed as a way to assess subject perception of changes in their UC-related symptoms over the course of treatment. A score of 180 or higher (out of a total possible score of 224) on the complete questionnaire is considered to be indicative of the subject being in a state of remission. Consistent with the “in remission” or mild disease classification, only 3 of the 28 enrolled subjects reported a score of <180 to begin with. Overall, there was little change in average group scores between Day-0 and study completion. In the subjects consuming Aquamin for 180 days, average scores on the complete questionnaire improved from 200 ± 13 to 204 ± 13 while in the placebo group, scores at initiation and post-intervention were 199 ± 20 and 200 ± 18. In subjects who crossed over to Aquamin for 90 days, the IBDQ score increased from 200 ± 18 to 202 ± 14. With the 10-question, bowel-specific survey, pre- and post-intervention scores were 64 ± 5 and 65 ± 4, respectively in the Aquamin-treatment group, while in the placebo group, pre- and post-intervention scores were 63 ± 8 and 63 ± 7, respectively. Similar trends were observed in the UCDAI, as most subjects remained in remission after completion of the intervention, except for one subject in the placebo group who experienced a flare with a score of 10. Pre- and post-intervention scores are presented in S2 Table.

At the end of their 180-day treatment period or at the 2-week post-intervention close-out call, subjects were given the opportunity to provide comments as to their perception of how the study had gone (S6 Table). A total of 19 subjects (10 from the group that had received Aquamin for the complete 180-day period and 9 from those who were in the placebo group for the first 90 days and Aquamin for the final 90 days) chose to respond. To summarize, eleven subjects reported feeling better or having “more energy” and five subjects noted improvements in their gastrointestinal symptoms. Three subjects reported reduced usage of prescribed UC medicines and two reported decreased musculoskeletal pain (S6 Table).

### Bone biomarker changes with Aquamin

**Fig 4:**
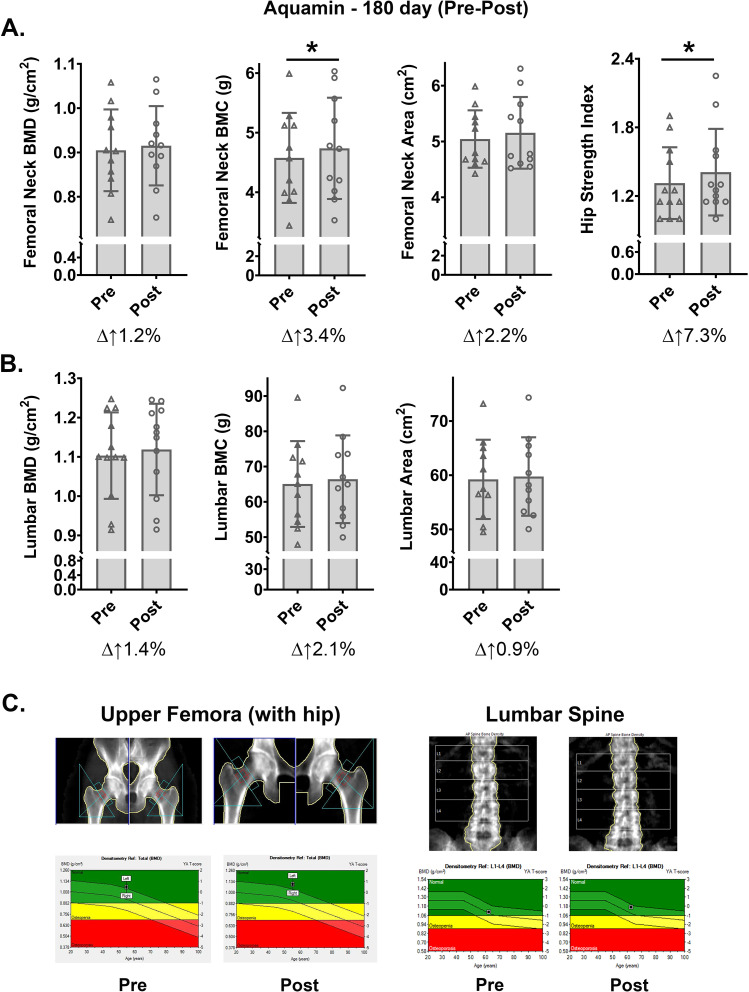
DEXA scan endpoints: Aquamin (180-day treatment group). A. Femoral neck. BMD, BMC and area values were obtained directly from DEXA scans. Hip strength index was calculated according to Yoshikawa et al [36]. B. Lumbar vertebrae. BMD, BMC and area values were read directly from DEXA scans. All values are treatment group means and standard deviations. Delta values shown beneath each pair of bars indicate percentage change in the post-treatment value relative to the pretreatment value. Statistical significance between pre- and post-treatment values was determined by paired t-test; asterisk (*) indicates p < 0.05. C. DEXA scan inserts: Scans of the Upper femora (left) and lumbar spine (L1-L4, right) were performed at Day 0 (pre-intervention) and Day 180 (post-intervention). The graphs at the bottom of these scans display BMD values for both the femora and spine. On the left, Aquamin improved femoral BMD by 3.4% in a 55-year-old patient, while on the right, lumbar BMD improved by 6% in a 63-year-old participant after 180 days of Aquamin intervention.

Because bone loss is a common occurrence in UC [5] and because our earlier preclinical studies in mice demonstrated inhibition of bone loss with Aquamin [43–45], each subject underwent DEXA scanning at initiation and at Day-180. Shown in Fig 4 are BMD and BMC values obtained from two regions of interest – i.e., femoral neck and lumbar (L1-L4) vertebrae – in subjects that had received Aquamin for the complete 180-day period. Changes were seen in both regions but findings from the femoral neck region were of particular interest. Over the course of the six-month interventional period, the average femoral neck BMD value increased by 1.2% while the average BMC value increased by 3.4% (statistically significant compared to baseline) (Fig 4A). The increase in BMC relative to BMD reflects an increase in surface area captured by the DEXA scan (calculated to be 2.2% increase compared to pretreatment values). These improvements in bone structure assessed by DEXA scan were sufficient to drive a 7.3% (statistically significant) increase in hip bone strength calculated according to the method described by Yoshikawa et al [36] (Fig 4A). When BMD, BMC and area were assessed in the LI-L4 vertebrae, 1.4%, 2.1% and 1% increases were seen in subjects who received Aquamin for 180 days (Fig 4B). Representative DEXA scans from both regions of interest – upper femora and lumbar spine – carried out at Day 0 and Day 180 are shown in Fig 4C. In contrast to these findings, there were essentially no changes in BMD and BMC values at either site in subjects who were on placebo for the first 90 days and then crossed over to Aquamin for the last 90 days (S2 Fig).

**Fig 5:**
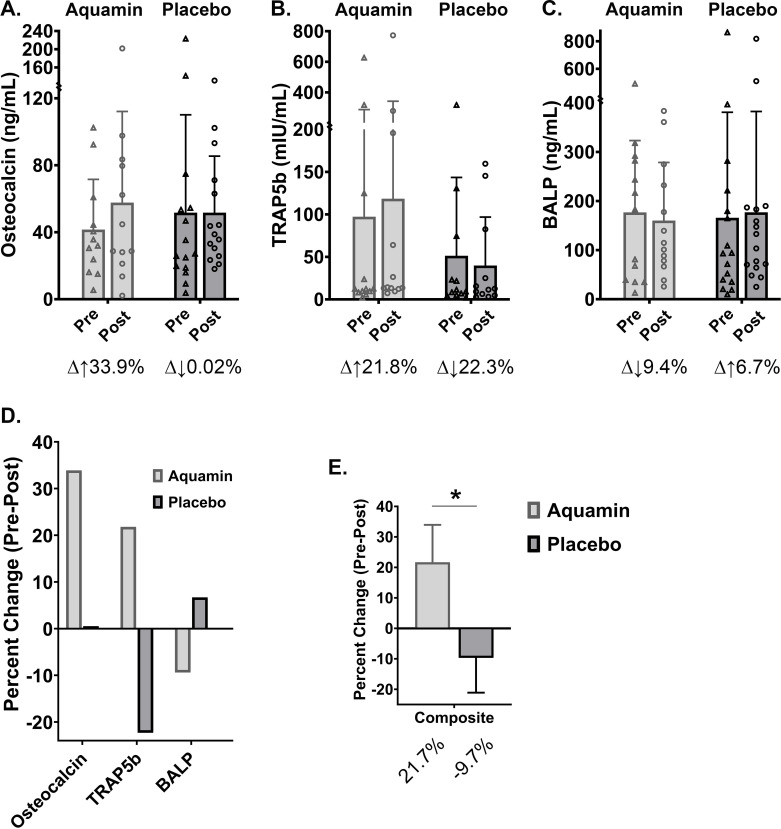
Bone-related serum chemistry endpoints: Aquamin (180-day treatment group) versus placebo. A. Serum osteocalcin: Values (ng/mL). B. Serum TRAP5b: Values (mIU/mL). C. Serum BALP: Values (ng/mL). All values were determined by ELISA (AssayGenie). Values are treatment group means and standard deviations. Delta values shown beneath each pair of bars indicate percentage change in the post-treatment value relative to the pretreatment value. D. Aquamin versus placebo comparison: Pre- and post-treatment differences for each endpoint are plotted for the two participant groups to show the divergence between the groups. E. A composite score was generated by combining the three individual endpoint scores. Statistical significance between group (composite) values was determined by unpaired t-test; asterisk (*) indicates p < 0.05. [Note: For the composite graph, inverse BALP values are plotted because while increased osteocalcin and TRAP5b are associated with improved bone metabolism, decreased BALP is reflective of improved bone metabolism].

As part of the effort to understand bone changes observed in the DEXA scans, serum levels of osteocalcin and TRAP5b were measured by ELISA. Fig 5A and 5B demonstrate changes in both proteins. In the subjects receiving Aquamin for 180 days, osteocalcin was increased by 34% and TRAP5b by 22%. In the placebo group, the average osteocalcin value was unchanged between pre- and post-treatment while the TRAP5b values decreased (22% reduction) (Fig 5A and 5B). In addition to osteocalcin and TRAP5b, we also assessed serum levels of bone-specific ALP (BALP). Consistent with previous findings [46], serum BALP accounted for a much greater percentage of total serum ALP than ALPI. Based on the average baseline values from all 28 subjects, BALP and ALPI levels were 170.4 ng/mL and 10.7 ng/mL, respectively. Among subjects who received Aquamin for 180 days, BALP decreased by 9.4%, whereas among subjects who received placebo, BALP values increased by 6.7% (Fig 5C). Fig 5D summarizes the pre- and post-treatment findings for each serum marker, comparing results from the Aquamin-treatment group and placebo. A composite index made up of the three independent markers is also shown (Fig 5E). The divergence in outcomes between the Aquamin-treatment and placebo is striking (p = 0.0316; unpaired t-test). Lastly, there was a decrease of 9% in serum osteocalcin, an increase of 4% in TRAP5b, and a decrease of 6% in BALP at the end of the study among individuals who began taking Aquamin at the Day-90 timepoint (after crossing over). These trends were less convincing compared to what was observed with Aquamin over the entire 180-day period but still improved over findings from the placebo group.

### Proteins of interest: Proteomic analysis

**Fig 6:**
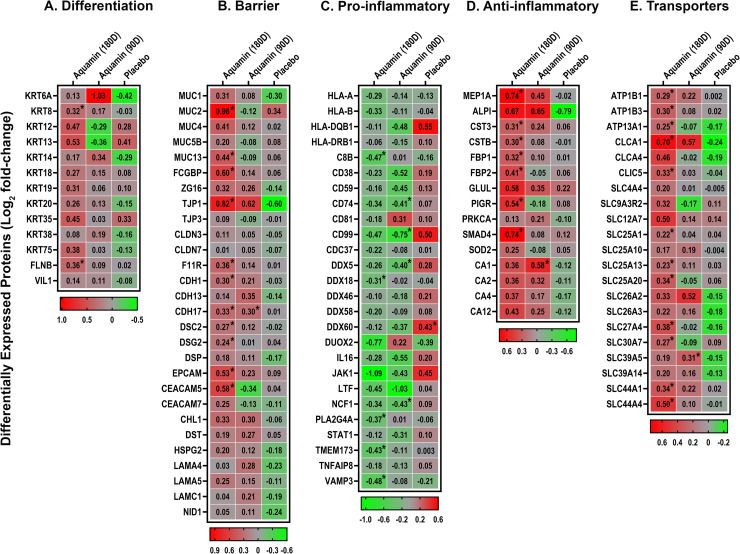
Proteins of interest identified in colon biopsies by directed search of the proteomic database. Proteins involved in A. Differentiation; B. Barrier formation; C and D. Inflammation and E. Transport are shown. Data are presented as a heatmap illustrating the pre- and post-intervention changes (up- or downregulation) of individual proteins of interest. Values reflect the Log_2_ abundance ratio relative to the respective pre-intervention levels for placebo (n = 16) and Aquamin treatment (n = 12) for 180 days (180D). Protein expression is also shown for subjects who crossed over from placebo and received Aquamin for the last 90 days (90D) (n = 16). Mean values for individual proteins were compared to their respective pre-intervention values for statistical differences using the limma package. An asterisk (*) indicates a significant difference from the respective baseline expression at p < 0.05.

Colonic biopsies obtained from each subject at Day-0, Day-90 and Day-180 were examined using a data independent acquisition mass spectrometry approach to protein profiling as described in the Materials and Methods Section. The proteomic screen was used as a way to determine if treatment with Aquamin would have a detectable effect on proteins that contribute to epithelial cell differentiation and barrier formation. In parallel, proteins that are inflammation-related (including those that directly influence pathophysiology and those that may simply be reflective of an inflammatory environment) were also assessed as were moieties that contribute to electrolyte transport and fluid balance control in the colon. Findings from our previous preclinical studies with human colon tissue in organoid culture [19–24] and our earlier biomarker and interventional trial with healthy adults [25] were used to guide the present assessment. Protein expression data from this directed search are presented in Fig 6 as heatmap panels.

Fig 6A and 6B demonstrate the upregulation of proteins involved in differentiation (multiple keratins and filamin) and barrier formation. Among barrier proteins, we saw enhanced expression of multiple cadherins, along with proteins involved in desmosome formation (i.e., DSC2, DSG2 and DSP). Tight junctional proteins, i.e., TJP1 (ZO1) and Junctional adhesion molecule A (JAM-A or F11R) were also strongly upregulated, but other components of tight junctions were only modestly induced or not detected. Both TJP1 and JAM-A play significant roles in tight junction formation and mucosal repair [47,48]. Several mucins and other cell adhesion molecules that make up the mucinous layer were also elevated with intervention. Among these were MUC2 and FCGBP. These proteins are secreted by goblet cells; reduced expression is consistent with structural weakening of the mucus barrier, and this may occur prior to the onset of inflammation [49]. Upregulation of these proteins with intervention is consistent, therefore, with improvement in the mucosal barrier structure. Another protein upregulated with intervention (i.e., ZG16) suggests decreased binding of bacteria to the colonic epithelium [49,50]. Additional adhesion molecules such as EPCAM and CHL1 (also known as L1CAM2) were also upregulated in response to Aquamin treatment, and their increased expression may have beneficial roles in immune homeostasis and barrier integrity [51,52]. Finally, components of the basement membrane were responsive to Aquamin. These results are consistent with findings from our previous colon organoid culture studies [19,21,22]. It is of interest to note that there was little change in expression levels for the majority of these same barrier proteins in the placebo group over the course of treatment (Fig 6B). Where pre- and post-treatment differences were observed in the placebo group, the majority of the proteins were downregulated post-treatment.

Fig 6C identifies a series of inflammation-related proteins that were downregulated in response to Aquamin treatment. Although most of the moieties identified in this panel have multiple functions, several of these proteins are known to either directly contribute to inflammation or are part of the innate inflammatory response. For example, membrane-associated Phospholipase A2 (PLA2G2A) is frequently upregulated in gastrointestinal tract inflammation and is associated with epithelial cell damage [53]; it was suppressed with Aquamin. Another potentially interesting example is the JAK1 protein; it was reduced by 2-fold with Aquamin while in the placebo group it was increased by 1.4-fold over the period of intervention. JAK1 is a tyrosine kinase and is critical for IFN-α/β signaling [54]. Among other Aquamin-sensitive proteins was LTF, a neutrophil granule protein [55]. The reduced level of this protein, like fCAL (Fig 3D above), is consistent with fewer neutrophils in the intestinal wall. Another Aquamin-downregulated protein – i.e., neutrophil cytosol factor 1 (NCF1; also known as p47phox) – is required for activation of the NADPH oxidase as part of superoxide production in neutrophils [56].

Another group of inflammation-related proteins was upregulated with Aquamin (Fig 6D). Proteins in this diverse group can play multiple roles in countering inflammation (e.g., have anti-protease or antioxidant activity, control antibody transport across cellular membranes, regulate cytokine generation) or may simply be a reflection of (in this case) a lessened inflammatory state. The upregulation of SMAD4 is of particular interest in that deletion of this moiety is strongly correlated with bowel inflammation [57] as is PIGR, which signals through SMAD activation and plays a critical role in gut immune homeostasis. Its reduced expression has been noted in connection with the intestinal inflammation associated with IBD [51,58]. MEP1A is another Aquamin-upregulated protein. Reduced MEP1A expression is correlated with increased disease activity [59]. The finding of increased ALPI (in the biopsy specimens) is also of interest. An elevated level of ALPI in the tissue biopsies may help explain the reduction in the amount of enzyme shed into the plasma. In tissue, loss of ALPI function or reduced expression is associated with intestinal inflammation [60]. Finally, the carbonic anhydrases are a family of enzymes that help maintain acid–base homeostasis, regulate pH, and fluid balance [61]. Their decreased expression has been associated with UC [62]; four isoenzymes were upregulated with Aquamin (Fig 6D).

Fig 6E shows expression levels for a number of integral membrane proteins that are responsible for establishing and maintaining electrochemical gradients. These proteins are essential for regulating the transport of a variety of organic solutes and inorganic minerals across plasma membranes and across intracellular membranes. As such, they play critical roles in osmoregulation in various tissues. In the colon, specifically, SLC26A3 (also known as DRA [downregulated in adenoma]) plays a critical role in anion transport and fluid absorption [63]. ATP1B1 and ATP1B3 also contribute to regulation of fluid absorption in the colon [64]. What role (if any) that each of these Aquamin-upregulated moieties presented in Fig 6E play in UC will require additional study.

It should be noted, finally, that we observed a high level of concordance between the two Aquamin cohorts in protein expression profiles; that is, the majority of proteins impacted by Aquamin at 180 days of treatment were also altered in the 90-day treatment cohort. The 90-day protein expression data are presented in the heatmap panels (Fig 6A-6E) along with data from the other treatment groups.

**Fig 7:**
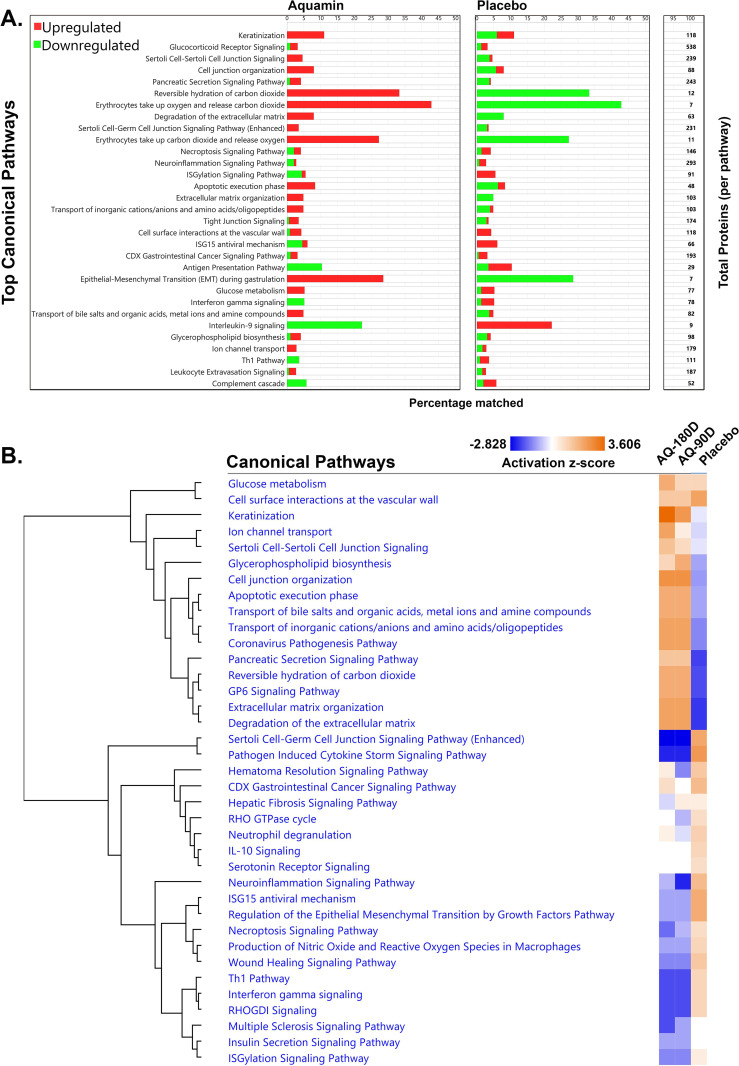
Canonical pathways. A. Top canonical pathways affected (activated or suppressed) by the proteins shown in Fig 6. Pathways were curated by QIAGEN Ingenuity^®^ Pathway Analysis (IPA) and sorted by p-value. The stacked bar chart displays the percentage of proteins in the database compared to the total number of proteins contributing to each pathway. Red bars depict upregulation, and green bars represent downregulation of proteins in each significantly enriched canonical pathway. The total number of proteins involved in each pathway is listed on the right margin of the graph. The left set of bars illustrates pathways influenced by Aquamin (180-day)-responsive proteins, while the right set shows how the same pathways were affected by the placebo. B. Comparative analysis of canonical pathways. Pathways from the three treatment groups were compared using z-score activation and prioritized with hierarchical clustering, visualized in a heatmap to show trends. Increased z-score activation is represented by orange color, while suppression is depicted by dark blue in the heatmap. Concordance is observed between the Aquamin groups (180- and 90-day).

Fig 7 identifies canonical pathways influenced by proteins shown in Fig 6. S7 Table provides the raw data upon which Fig 7 is constructed. These pathways (curated by Qiagen IPA) were identified based on the p-value of the overlap between identified proteins in the database (i.e., in Fig 6) and proteins in each given pathway (Fig 7A). Keratinization, Cell Junction Organization, Extracellular Matrix Organization, Transport of Inorganic Cations/Anions and Amino Acids/Oligopeptides and Ion Channel Transport are among the top pathways identified in this manner. These are upregulated with Aquamin [with z-score greater than 2.2 and –log(p-value) of 2.75]. The z-score for the same pathways ranged from –0.28 to –2.3 with placebo treatment suggesting downregulation (S7 Table). A key feature of Fig 7A is the strong dichotomy between the two interventions (Aquamin treatment for 180 days and placebo).

A comparative analysis of canonical pathways among all three treatment groups based on z-score activation is shown in Fig 7B. The key feature of this heatmap is the strong concordance between the two Aquamin-treatment groups and divergence of both with placebo. Some of the altered pathways listed include Pathogen Induced Cytokine Storm Signaling Pathway, Th1 Pathway, Interferon gamma signaling, Necroptosis Signaling Pathway, ISGylation Signaling Pathway, Neutrophil Extracellular Trap Signaling Pathway, Production of Nitric Oxide and Reactive Oxygen Species in Macrophages, ISG15 antiviral mechanism, Neuroinflammation Signaling Pathway, and IL-10 Signaling. These inflammation-related pathways were upregulated in placebo samples while Aquamin downregulated the majority of pathways (Fig 7B). S7 Table provides the data from which Fig 7 was constructed.

Additional analyses of the proteomic findings are presented in the supporting information section. S3 Fig shows the distribution of proteins presented in Fig 6 by means of volcano plots. S3 Fig demonstrates the extent of up- or downregulation (abundance ratio) for each protein and the associated p-value for each protein in response to Aquamin (90- or 180-day) treatment or placebo. The divergence of the placebo group from the two Aquamin-treatment groups is apparent.

**Fig 8:**
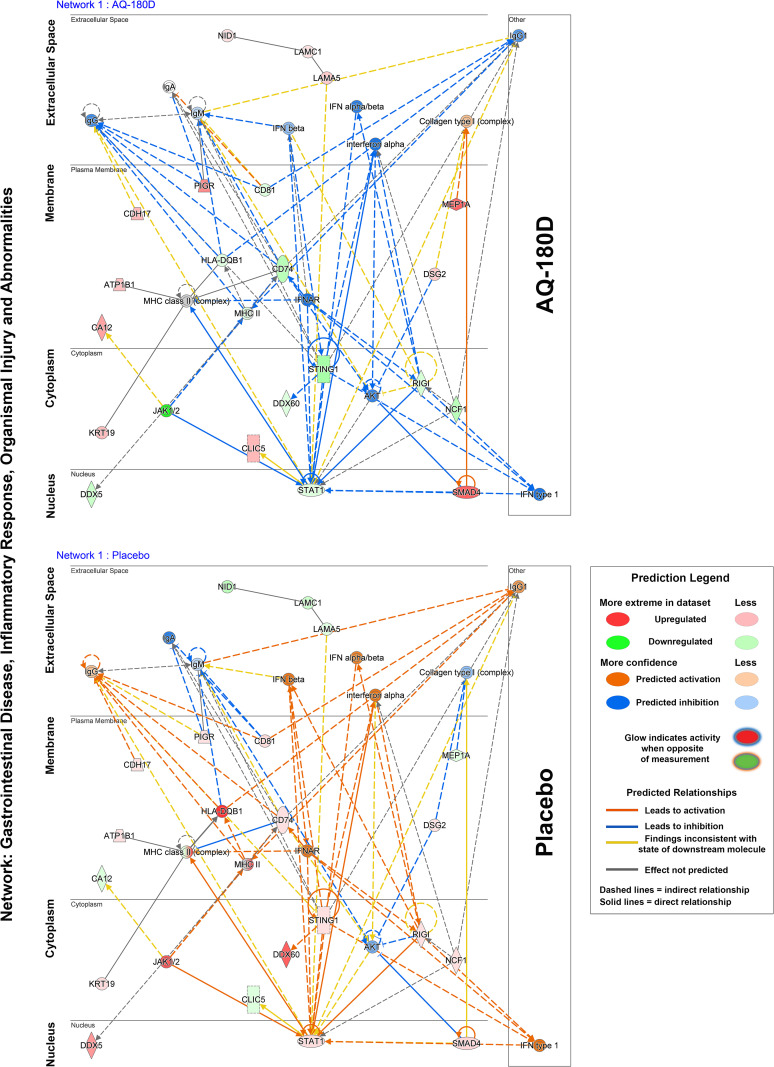
A subcellular layout of IPA-generated network analysis. One of the top networks, illustrated in Fig 8, is associated with gastrointestinal disease, inflammatory response, and organismal injury (Network 1). This network comprises 21 molecules from our dataset with a score of 39. Each network is limited to 35 molecules (IPA default setting) to ensure easy interpretability. The likelihood of these molecules being part of the network is indicated by a p-value calculation. The networks were generated by proteins altered by Aquamin (**top**) and Placebo (**bottom**). The color-coded figure legend explains the confidence levels associated with the predicted activity and interaction among these molecules, which are based on their measured expression levels. Notably, the network molecular expression and interactions among these molecules in the Aquamin and placebo groups are completely opposite. The figure legends use color coding to represent the expression and interactions with dark and light colors, respectively, to illustrate the predicted differences. The raw data for the network analysis is provided in S8 Table.

The Qiagen IPA analysis tool allows one to identify functional protein networks and to use the identified networks to predict involvement in biological functions and disease processes. The top protein network identified in this manner (Gastrointestinal Disease, Inflammatory Response, Organismal Injury and Abnormalities) (Fig 8) reveals a network of pro-inflammatory molecules reduced in expression by Aquamin, while the same molecules are upregulated in placebo. The raw data for this network analysis is provided in S8A-S8D Table in S8 Table. S8A Table in S8 Table identifies the seven top functional networks identified in this manner. S8B and S8C Tables in S8 Table present a list of molecules involved and relationships of their interactions in the top network. S8D Table in S8 Table presents disease or function annotations along with the predicted p-values for the molecules involved in these processes. Interestingly, colitis is listed as one of the top diseases with these altered molecules. Increased expression of CD74, NCF1, STING1 (TMEM173), and HLA-DQB1, as well as decreased expression of MEP1A, are the underlying causal molecules in placebo samples. Aquamin reversed their expression in virtually all instances. In summary, these proteomic data demonstrate that Aquamin markedly increased the expression of proteins involved in epithelial barrier integrity while decreasing the expression of pro-inflammatory proteins in colonic tissue. In contrast, placebo-treated samples showed little improvement in barrier proteins and upregulation of inflammatory pathways, highlighting Aquamin’s potential dual role in strengthening the mucosal barrier and reducing inflammation.

## Discussion

The present study is part of an effort to determine if (and to what extent) a multi-mineral intervention derived from marine red algae could have a beneficial impact on individuals with UC. Over the 180-day treatment period, there were shifts in the levels of several individual biomarkers that, together, suggest the potential for disease improvement. Among the changes seen were reductions in serum ALP (both total and intestine-specific), as well as serum CRP and fCAL (markers of chronic inflammation and neutrophil accumulation in the colon [30–33,42], respectively). The reduction in fCAL was accompanied by fewer tissue neutrophils as observed in histological sections of colon biopsies. Pre-, post-treatment differences in ALP reached a level of statistical significance, but while the improvements observed with CRP and colon neutrophil markers did not, improvements were not observed in the placebo group with any of these endpoints. This was clearly seen when a composite score based on the five biomarkers was generated for the Aquamin-treatment and placebo groups (Fig 3H). In addition to modulation of disease-related biomarkers, there was subjective evidence (i.e., participant responses at the end of the intervention period) for efficacy. Taken together, these findings suggest that even the low level of residual disease in our subject population may be amenable to further amelioration by inclusion of a multi-mineral intervention in the maintenance regimen.

As part of the present study, a proteomic screen was used to assess changes in a broad range of potentially relevant proteins. UC subjects who received Aquamin for 180 days demonstrated upregulation of several proteins that contribute to the gastrointestinal barrier. In parallel, proteins associated with inflammation were downregulated, while several proteins with anti-inflammatory potential were upregulated. Inflammation is, unarguably, a major contributor to barrier breakdown in UC [1,2]. However, barrier dysfunction has been noted in UC patients even in the absence of inflammation [49,65]. Further, our own prior studies have demonstrated improved barrier structure and function in organoids from healthy subjects [19,20], as well as increased barrier protein expression in biopsies from healthy human subjects following Aquamin intervention [25]. Based on this, we hypothesize that Aquamin might improve gastrointestinal health in UC patients (who usually have a compromised barrier) by strengthening the colonic barrier. This effect could result either from a direct upregulation of barrier-associated proteins or from an anti-inflammatory effect.

Finally, both in the studies reported here and in our past studies [22,23], Aquamin treatment modulated expression of several cell surface transporter proteins, including moieties that help regulate fluid resorption in the colon. This activity could help to improve UC symptomatology even without affecting a major change in trans-epithelial barrier function and/or inflammation. While the present study did not address this issue directly, several subjects self-reported improvements in bowel function as well as an overall improvement in “well-being” at their final visit (see S6 Table).

While the primary manifestations of UC are inflammation and superficial ulcer formation in the colonic wall, complications of the disease can be seen in other tissues. Bone loss is a common occurrence [5] and managing this is always a challenge [66]. The pathophysiology of UC-related bone loss is not completely understood, but systemic inflammation and poor mineral absorption in the gut are both thought to be contributing factors [67]. Given this understanding, subjects in our trial underwent DEXA scanning at baseline and at the Day-180 timepoint. The response of the femur to Aquamin treatment suggests benefits to bone strength in two ways. First, the increase in femoral neck BMC indicates increased bone accrual over the 6-month time frame. Second, the increase in femoral neck area implies new bone deposition on the periosteal surface. Both effects would be expected to increase bone strength. These two benefits together were sufficient to drive a statistically significant (7.3%) increase in the calculated hip bone strength. With pharmaceutical interventions, improvements of this magnitude in BMD and BMC values often require 2–3 years [68]. Improvements in BMD and BMC in the lumbar spine (L1-L4) were also evident, but the ratio of BMC to BMD was not as striking as that seen in the femoral neck. This was not unexpected, since the relative amount of trabecular bone compared to cortical bone in the vertebrae is higher than in the femoral neck. Still, the greater increase in BMC compared to BMD is an indication of an increase in bone surface area and provides a rationale for increased strength [69].

Along with the changes seen by DEXA scan, we also observed increases in both osteocalcin (osteoblast marker) and TRAP5b (osteoclast marker) in serum (Fig 5A and 5B). At the same time, bone-specific ALP (an indicator of high bone turnover in several conditions including UC [70]) decreased in the 180-day Aquamin cohort (Fig 5C). A composite score comprising the three bone markers demonstrated the divergence between Aquamin-treatment and placebo (Fig 5D). If similar results to those shown here can be observed in a larger trial, the benefit to bone health would be sufficient, we believe, to justify consideration for use in UC, regardless of how much improvement in colon-specific biomarkers were seen. The potential for reduction in bone loss independent of UC should also be considered.

Mechanism(s) contributing to bone improvement cannot be determined from the present work, but our previous studies in mice may provide insight. In preclinical studies, dietary Aquamin supplementation reduced bone mineral loss and improved bone strength and stiffness [43–45]. In those studies, there were changes in the bone content of several cationic elements. The most striking change was a large (6- to 10-fold) increase in bone strontium. When available, strontium replaces some of the calcium in the bone lattice to produce a crystalline structure that is harder [71,72]. Since strontium is one of the most prevalent cationic elements in Aquamin (S1 Table), the beneficial activity of Aquamin on bone may reflect increased mineralization *per se*. At the same time, previous work has shown that strontium stimulates the activity of both osteoblasts and osteoclasts to enhance bone turnover [73,74]. Aquamin’s main action, therefore, may be mediated through effects on bone cell metabolism. While strontium (and calcium, of course [75]) are central to the beneficial effects of Aquamin on bone health, previous studies have suggested that other trace elements also contribute to bone health [76,77]. These minerals are also present in Aquamin (S1 Table). Finally, inflammation itself is a contributor to bone loss [66,67]; thus, any reduction in systemic inflammation achieved by mineral supplementation (either directly or indirectly) could beneficially impact bone. Unfortunately, the experimental approaches needed to distinguish between these and other possibilities are not readily amenable to the type of study conducted here.

Hepatobiliary manifestations are frequently observed in patients with UC [3,4]. Inflammation of the bile ducts with potential for scarring is a concern in UC. Up to 80% of individuals diagnosed with primary sclerosing cholangitis (PSC) have an underlying UC while up to 5% of individuals with UC have some degree of biliary tract inflammation [4,78,79]. Often there is evidence of bile duct involvement before bowel symptoms are present [80]. Since this study did not directly assess liver involvement and there is no readily available way to identify or quantify the hepatic contribution to total serum ALP levels, the present study provides no direct evidence to suggest that Aquamin benefits the liver. Analogous to what was seen with bone, however, our long-term (15–18 month) studies in mice demonstrated beneficial effects in the liver with dietary Aquamin. This included a reduction in liver inflammation along with reduced fibrotic changes, an almost complete elimination of tumor formation [17] and a decrease in toxic bile acids [81]. In a 20-week follow-up study in high-fat diet-fed mice [82], Aquamin reduced the production of toxic lipid intermediates and oxidants in association with a reduction in liver inflammation seen histologically. Finally, in our trial with healthy subjects, Aquamin reduced the total bile acid content in the colon as well as levels of several toxic secondary bile acids [27]. These past findings demonstrate that mineral supplementation can benefit the liver, but whether positive effects in the liver will be seen clinically requires further investigation.

Taken together, the data from this study demonstrate that daily ingestion of a marine algae-derived multi-mineral product has the potential to improve several biomarkers of gastrointestinal health, as well as bone health, in UC patients. While in our view these findings support continued research into the use of Aquamin as an ancillary intervention in UC, there were obvious limitations to the study. The number of subjects (twenty-eight in total) was small; the duration of treatment (180 days) was relatively short; and the interventional agent was given at a low dose (i.e., standardized to provide 800 mg of calcium per day). Additionally, subjects in the trial were from a cohort with UC in remission or at the mild disease stage. All of these deficiencies could be addressed with a larger trial. Our hope is to conduct a 1–2 year open-label trial, pending FDA approval for such an effort. The lack of significant drug-related adverse events, the absence of negative changes in the comprehensive serum chemistry panel and the absence of tolerability issues should support approval for a longer-term study.

Finally, and perhaps, most importantly, a subsequent trial could include patients with moderate to severe UC. Because we envision the multi-mineral supplement to be used as an ancillary treatment, more seriously ill patients could remain on their primary therapy as dictated by standard of care while also consuming the multi-mineral supplement. The mineral supplement might help not only to counteract pathophysiological events linked directly to inflammation and tissue damage but, with its potential for improving barrier integrity, could retard rapid drug loss through the “leaky gut,” which is an issue in some patients with active UC [83,84]. Either effect would be beneficial.

Another issue is the incomplete understanding of potential therapeutic mechanisms. While the current study and past work all support barrier improvement—by demonstrating upregulation of proteins involved in barrier structure—as a primary effect of Aquamin, there is no direct evidence at this point to substantiate improvement of barrier function in UC patients. To address this important issue, we have begun a study to measure gastrointestinal permeability in subjects with UC using the urine lactulose-mannitol ratio approach [85] (clinicaltrials.gov: NCT04855799). When complete, this study should go a long way toward establishing the relationship between the protein changes seen here and functional changes in the gut barrier.

## Conclusions

In summary, preclinical studies have provided compelling evidence that daily ingestion of a multi-mineral product can improve barrier structure and function and reduce pro-inflammatory changes in the gastrointestinal tract. Consistent with this past body of evidence, the results presented here (and summarized in the graphical abstract) demonstrate that the same multi-mineral product improves disease-related biomarkers in patients with UC. Continued development as an ancillary treatment in UC is justified, we believe, by this work. We envision using Aquamin standardized to deliver no more than 1,000 mg of calcium per day. Aquamin dosage standardized to deliver this amount of calcium would be within the range approved for long-term use as a dietary supplement. At this level, beneficial effects of calcium may be seen without the unwanted side effects sometimes observed with higher calcium supplement intake [86]. At the same time, the wide range of additional trace minerals in the product could provide benefits to UC patients that are not achievable with calcium alone [87]. While, hopefully, the benefits would not be limited to individuals with minimal disease, people with UC in remission or those with mild to moderate disease might benefit most from this approach. Current therapeutic development is focused mostly on addressing severe disease. Even though mild and moderate disease represents approximately 70% of the population with UC, there have been no therapeutic improvements to specifically address mild or less severe UC since the introduction of mesalamine in the 1980s [7]. Identifying ways to impact mild to moderate disease is essential. Finally, given its favorable safety profile and potential benefits in improving barrier integrity and mitigating inflammation, Aquamin may also play a role in the prevention of UC, which warrants additional studies.

## Supporting information

S1 Fig Chemistry and histology endpoints: Aquamin (90-day treatment group) versus placebo. A. Serum alkaline phosphatase (ALP): Values (U/L) determined as part of the serum chemistry panel. B. Serum intestine-specific alkaline phosphatase (ALPI): Values (ng/mL) determined by ELISA. C. C-reactive protein (CRP): Values (mg/dL) determined as part of serum chemistry panel. D. Fecal calprotectin (fCAL): Values (µg/g) determined by ELISA (BÜHLMANN). E. Histological assessment: Values are presented as a simplified Geboes score. All values are treatment group means and standard deviations. Delta values shown beneath each pair of bars indicate percentage change in the post-treatment value relative to the pretreatment value. F. Aquamin versus placebo comparison: Pre- and post-treatment differences for each endpoint are plotted for the two participant groups to show the divergence between the two groups. G. A composite score was generated by combining the five individual endpoint scores.
(TIF)

S2 Fig DEXA scan endpoints: Placebo plus Aquamin (90-day treatment group). As no DEXA was performed at the 90-day visit, this group represents subjects who were on placebo for the first 90 days and then on treatment after crossing over to Aquamin at Day-90. A. Femoral neck. BMD, BMC, and area values read directly from DEXA scans. Hip strength index was calculated according to Yoshikawa et al [36]. B. Lumbar vertebrae. BMD, BMC and area values read directly from DEXA scans. All values are placebo plus Aquamin treatment group means and standard deviations. Delta values shown beneath each pair of bars indicate percentage change in the post-treatment value relative to the pretreatment value.
(TIF)

S3 Fig Protein distribution. Proteins presented in Fig 6 are shown in the volcano plots. Top. Aquamin treatment group (180-day), Middle. Aquamin treatment group (90-day), Bottom. Placebo. The abundance ratio (x-axis) for each protein is presented in Log_2_-transformed values and p-value (y-axis) is presented in –Log_10_ values. Red dots represent upregulated proteins, and green dots represent downregulated proteins.
(TIF)

S1 Table Aquamin Mineral Composition. (PDF)

S2 Table Mayo Score – Disease Activity Index for Ulcerative Colitis (UCDAI). (PDF)

S3 Table Disease Status and Drug Log (List of maintenance medications). (XLSX)

S4 Table Demographics. (PDF)

S5 Table Treatment-related reportable adverse events. (PDF)

S6 Table Subjective Feedback. (PDF)

S7 Table Canonical Pathways. (XLSX)

S8 Table Functional networks identified by network analysis in IPA. (XLSX)

S1 Text File. Full Trial Protocol. (PDF)

S2 Text File. CONSORT checklist. (PDF)

S3 Text File. Technical details of proteomic analysis. (PDF)

S1 Material The graphical abstract visually summarizes the study’s key findings, emphasizing markers associated with the UC disease, relevant underlying mechanisms, and the observed impacts on colon and bone health. (TIF)
