# Supplementary figures and images for: A multi-mineral intervention to improve disease-related and mechanistic biomarkers in ulcerative colitis patients: Results from a randomized trial
Source: PLoS One. 2025 Dec 8;20(12):e0337408. doi: 10.1371/journal.pone.0337408 (PMC12685183; doi:10.1371/journal.pone.0337408)

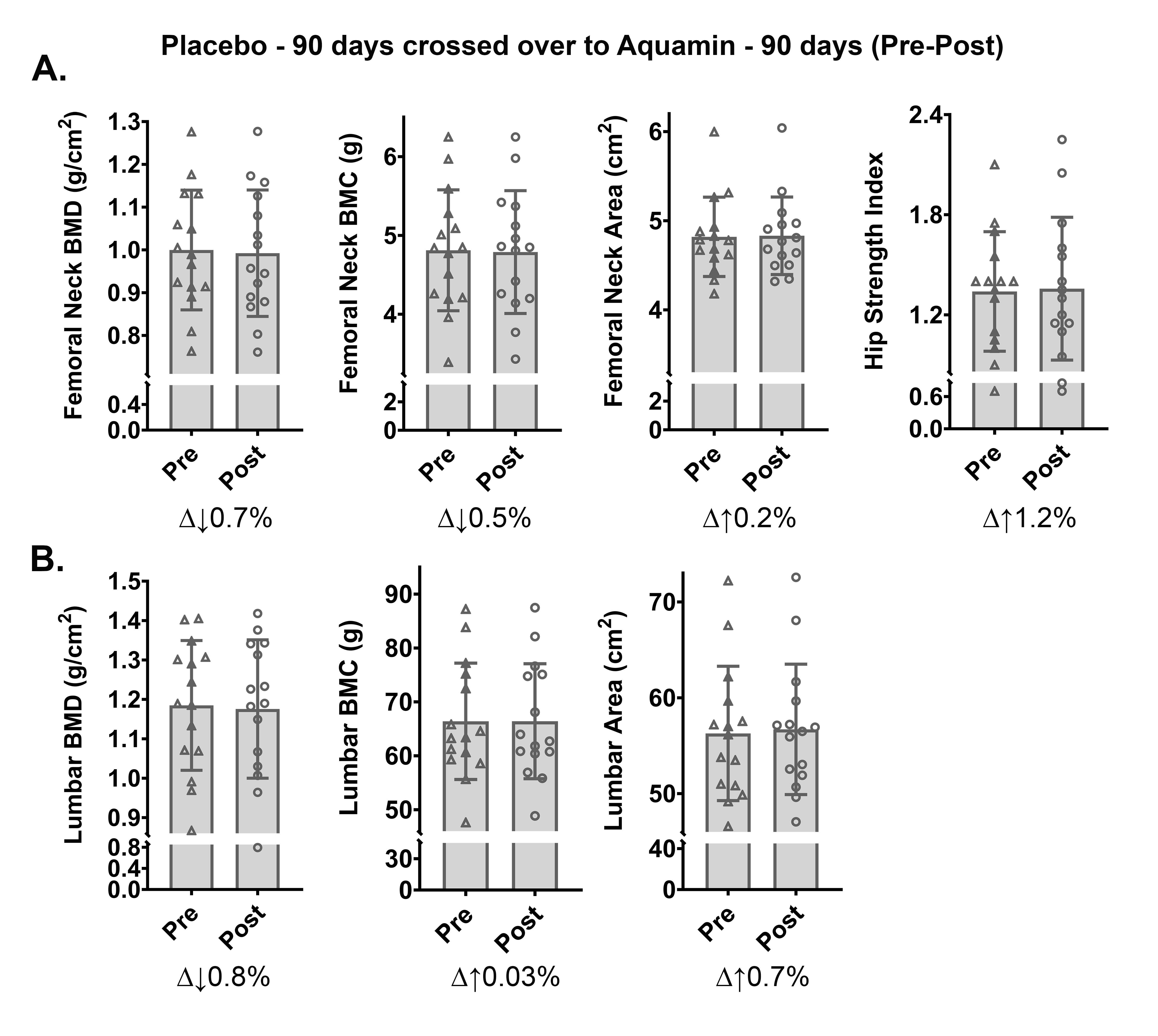

Supplement: S2 Fig — As no DEXA was performed at the 90-day visit, this group represents subjects who were on placebo for the first 90 days and then on treatment after crossing over to Aquamin at Day-90. A. Femoral neck. BMD, BMC, and area values read directly from DEXA scans. Hip strength index was calculated according to Yoshikawa et al [36]. B. Lumbar vertebrae. BMD, BMC and area values read directly from DEXA scans. All values are placebo plus Aquamin treatment group means and standard deviations. Delta values shown beneath each pair of bars indicate percentage change in the post-treatment value relative to the pretreatment value. (TIF) [file pone.0337408.s002.tif]

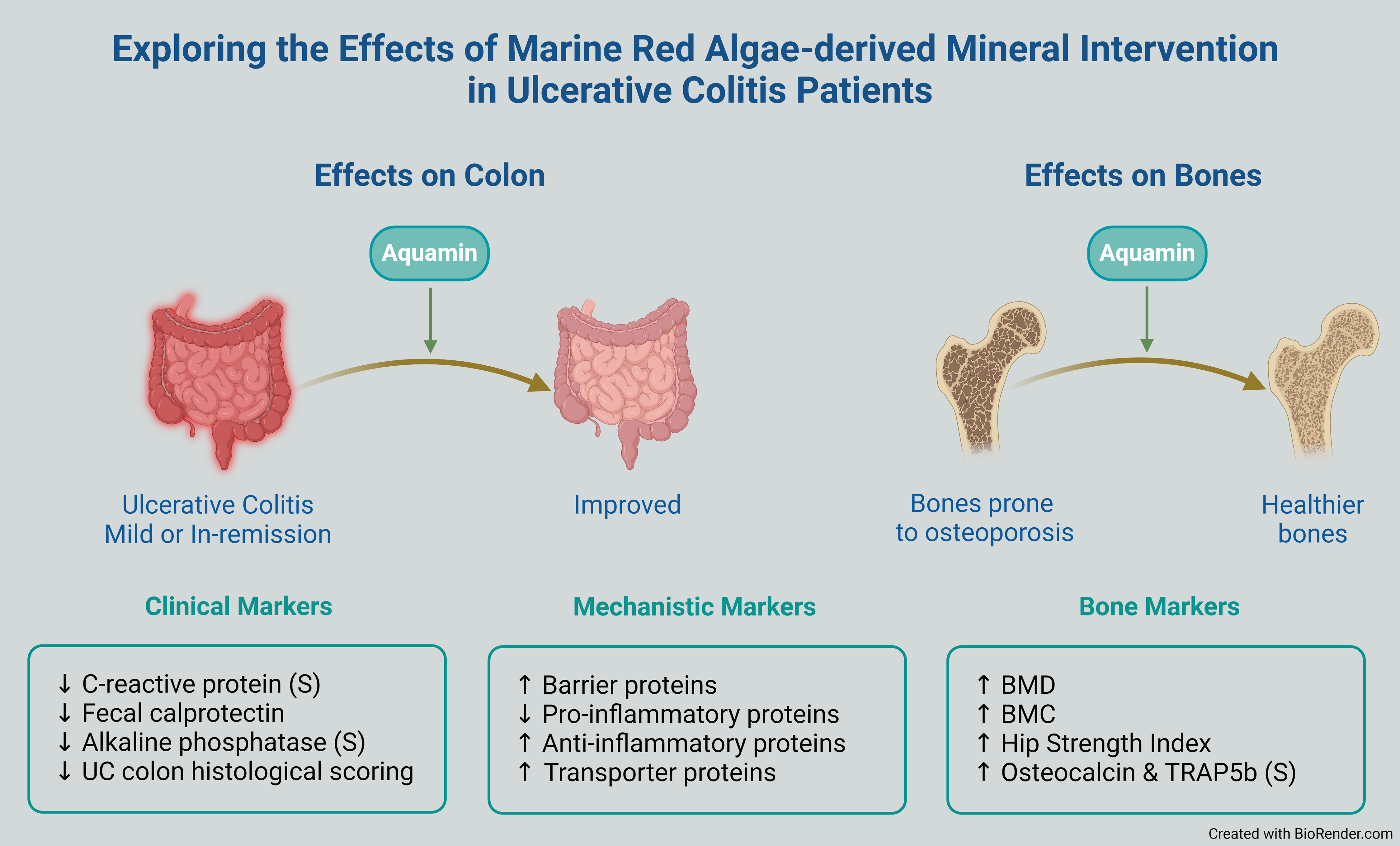

Supplement: S1 Material — (TIF) [file pone.0337408.s015.tif]
