# Supplementary material for: A multi-mineral intervention to improve disease-related and mechanistic biomarkers in ulcerative colitis patients: Results from a randomized trial
Source: PLoS One. 2025 Dec 8;20(12):e0337408. doi: 10.1371/journal.pone.0337408 (PMC12685183; doi:10.1371/journal.pone.0337408)
Supplement: S1 Table — (PDF) [file pone.0337408.s004.pdf]

Supplement Table 1. Composition of Aquamin® TG: Daily element intake

Element	μg/day	Element	μg/day	Element	μg/day
Aluminum	189	Hafnium	0.3	Rubidium	0.06
Antimony	<1.3	Holmium	0.1	Ruthenium	5.2
Arsenic	3.1	Indium	<0.003	Samarium	0.3
Barium	19	Iodine	0.3	Scandium	4.4
Beryllium	4.6	Iridium	<0.003	Selenium	<1.3
Bismuth	<1.3	Iron	1562	Silicon	233
Boron	88	Lanthanum	5.1	Silver	9.1
Cadmium	1.7	Lead	0.4	Sodium	12528
Calcium	800000	Lithium	<1.3	Strontium	6033
Carbon	317949	Lutetium	0.1	Sulfur	8264
Cerium	2	Magnesium	66154	Tantalum	0.02
Cesium	0.005	Manganese	111	Tellurium	<1.3
Chloride	5903	Mercury	<0.003	Terbium	0.06
Chromium	7.7	Molybdenum	<1.3	Thallium	<1.3
Cobalt	4.8	Neodymium	1.2	Thorium	11
Copper	8.4	Nickel	4.6	Thulium	0.04
Dysprosium	0.4	Niobium	31	Tin	0.6
Erbium	0.3	Osmium	0.003	Titanium	82
Europium	0.1	Palladium	1.2	Tungsten	<1.3
Fluoride	9	Phosphorous	200	Vanadium	9.4
Gadolinium	0.3	Platinum	<0.003	Ytterbium	0.2
Gallium	0.3	Potassium	513	Yttrium	9.4
Germanium	<0.003	Praseodymium	0.3	Zinc	7.8
Gold	<1.3	Rhenium	0.01	Zirconium	14
		Rhodium	1		

The trace mineral composition of Aquamin® TG was determined by an independent laboratory (Advanced Laboratories, Inc., Salt Lake City) for Marigot Limited (Ireland) in 2018.

Individual trace element composition was determined by Inductively Coupled Plasma Optical Emission Spectroscopy (ICP-OES) except for carbon (determined by ASTM D-1552), chloride and iodine (determined by Titration), and fluoride (determined by AOAC 939.11).

Values are presented based on daily consumption of Aquamin® normalized to provide 800 mg of calcium.
