# Supplementary material for: A multi-mineral intervention to improve disease-related and mechanistic biomarkers in ulcerative colitis patients: Results from a randomized trial
Source: PLoS One. 2025 Dec 8;20(12):e0337408. doi: 10.1371/journal.pone.0337408 (PMC12685183; doi:10.1371/journal.pone.0337408)
Supplement: S2 Table — (PDF) [file pone.0337408.s005.pdf]

Supplement Table 2. Mayo Score - Disease Activity Index for Ulcerative Colitis (UCDAI)

Subject ID	*Assignment*	Pre	Post	*Subject ID*	*Assignment*	Pre	Post
Subject 1	Placebo	0.0	1.0	Subject 17	AQ-180d	2.0	0.0
Subject 2	Placebo	4.0	0.0	Subject 18	AQ-180d	0.0	1.0
Subject 3	Placebo	0.0	0.0	Subject 19	AQ-180d	2.0	0.0
Subject 4	Placebo	2.0	0.0	Subject 20	AQ-180d	3.0	4.0
Subject 5	Placebo	0.0	0.0	Subject 21	AQ-180d	0.0	0.0
Subject 6	Placebo	0.0	0.0	Subject 22	AQ-180d	0.0	2.0
Subject 7	Placebo	0.0	3.0	Subject 23	AQ-180d	0.0	2.0
Subject 8	Placebo	5.0	3.0	Subject 24	AQ-180d	0.0	0.0
Subject 9	Placebo	2.0	10.0	Subject 25	AQ-180d	3.0	4.0
Subject 10	Placebo	1.0	1.0	Subject 26	AQ-180d	0.0	2.0
Subject 11	Placebo	0.0	0.0	Subject 27	AQ-180d	1.0	0.0
Subject 12	Placebo	0.0	0.0	Subject 28	AQ-180d	0.0	2.0
Subject 13	Placebo	2.0	2.0				
Subject 14	Placebo	2.0	0.0				
Subject 15	Placebo	1.0	0.0				
Subject 16	Placebo	1.0	0.0				
	Mean	*1.3*	*1.3*		*Mean*	*0.9*	*1.4*
	ST Dev	*1.5*	*2.6*		*ST Dev*	*1.2*	*1.5*

Pre: Pre-intervention, Post: Post-intervention. AQ: Aquamin®. Subject IDs are fictitious.

Mayo Score (UCDAI) assesses the severity of ulcerative colitis based on rectal bleeding, stool frequency, physician's global assessment, and mucosal appearance at endoscopy.

Eligible individuals were in remission or had mild disease at enrollment, as determined by the study gastroenterologist, with stable maintenance therapy and a Mayo score of ≤5.

For this study, endoscopic assessment was limited to the sigmoid colon, as flexible sigmoidoscopy allowed visualization of the colon up to 20 cm from the anus.
