# Supplementary material for: A multi-mineral intervention to improve disease-related and mechanistic biomarkers in ulcerative colitis patients: Results from a randomized trial
Source: PLoS One. 2025 Dec 8;20(12):e0337408. doi: 10.1371/journal.pone.0337408 (PMC12685183; doi:10.1371/journal.pone.0337408)
Supplement: S4 Table — (PDF) [file pone.0337408.s007.pdf]

Supplement Table 4. Demographics for subjects who completed 180-Day intervention

A. Study cohort demographics:

	**Female**		**Male**		**Both Genders**	
**Ethnic Category**	N	%	N	%	Total	%
Hispanic or Latino	0	0.0%	0	0.0%	0	0.0%
Not Hispanic or Latino	16	57.1%	11	39.3%	27	96.4%
Unknown	0	0.0%	0	0.0%	0	0.0%
Prefer not to answer	1	3.6%	0	0.0%	1	3.6%
**Total**	**17**	**60.7%**	**11**	**39.3%**	**28**	**100.0%**
**Racial Category** *(single category per participant)*	N	%	N	%	Total	%
American Indian/Alaska Native	0	0.0%	0	0.0%	0	0.0%
Asian	0	0.0%	0	0.0%	0	0.0%
Native Hawaiian or Other Pacific Islander	0	0.0%	0	0.0%	0	0.0%
Black or African American	1	3.6%	0	0.0%	1	3.6%
White	16	57.1%	10	35.7%	26	92.9%
Other	0	0.0%	1	3.6%	1	3.6%
Unknown	0	0.0%	0	0.0%	0	0.0%
**Total**	**17**	**60.7%**	**11**	**39.3%**	**28**	**100.0%**
**Age at Enrollment Category**	N	%	N	%	Total	%
18 - 21 years	1	3.6%	1	3.6%	2	7.1%
22 - 29 years	3	10.7%	3	10.7%	6	21.4%
30 - 39 years	2	7.1%	4	14.3%	6	21.4%
40 - 49 years	4	14.3%	1	3.6%	5	17.9%
50 - 59 years	2	7.1%	0	0.0%	2	7.1%
60 - 69 years	3	10.7%	2	7.1%	5	17.9%
70 - 79 years	2	7.1%	0	0.0%	2	7.1%
> 80 years	0	0.0%	0	0.0%	0	0.0%
**Total**	**17**	**60.7%**	**11**	**39.3%**	**28**	**100.0%**

B. Gender and Age:

	Gender	Age (Y)
Placebo (n=16)	M:6 / F:10	43.1±15
Aquamin (n=12)	M:5 / F:7	50.1±18

Age presented in years at the start.

C. Body Mass Index:

	BMI - Weight/Height^2 (Kg/m^2)	
	Pre	Post
Placebo	25.3±5.1	25.5±5.2
Aquamin	26.8±4.7	27.1±4.5

D. Calcium Intake Levels at the start (Estimated by DHQ3):

	Calcium (mg/day)
Placebo (P)	1134±486
Aquamin (AQ)	1083±439

Subjects above 1000 mg/day Calcium: 8 (Placebo / AQ-90Day) and 7 (AQ-180Day)
