# Supplementary material for: A multi-mineral intervention to improve disease-related and mechanistic biomarkers in ulcerative colitis patients: Results from a randomized trial
Source: PLoS One. 2025 Dec 8;20(12):e0337408. doi: 10.1371/journal.pone.0337408 (PMC12685183; doi:10.1371/journal.pone.0337408)
Supplement: S5 Table — (PDF) [file pone.0337408.s008.pdf]

Supplement Table 5. Treatment-Related Reportable Adverse Events

Event	*Placebo**	*AQ-90d**	AQ-180d
Number of subjects participated	16	16	12
Number of subjects reporting events	14	7	12
Total number of adverse events	39	14	44
COVID-19 infection	0	1 (1)	3 (3)
Sore throat (with enlarged lymph nodes)	0	1 (1)	1 (1)[a]
Earache	0	0	1 (1)[a]
Neuropathy	1 (1)	0	0
Anemia (low iron)	0	0	1 (1)
Elevated blood pressure	1 (1)	0	1 (1)
Dizziness (light headedness)	2 (2)[b]	0	0
Headache	3 (3)	1 (1)	1 (1)
Bilateral flank pain	1 (1)	0	0
Uterine Polyp (with bleeding)	1 (1)	0	0
Fatigue	1 (1)	1 (1)	2 (2)[c]
Back pain	0	1 (1)	0
Joint pain	0	0	5 (1)
Physical injury (car accident)	0	1 (1)	0
Tooth infection	0	0	1 (1)
Tooth removal	0	1 (1)	0
Jaw discomfort (with increased clenching)	0	1 (1)	0
Gastrointestinal events			
Nausea	3 (2)	0	0
Flatulence (& bloating)	20 (9)	1 (1)	21 (9)
Abdominal Pain (gastric discomfort)	4 (4)	1 (1)	3 (3)[d]
Constipation	0	2 (1)	0
Diarrhea (loose stool)	1 (1)	0	0
Blood in stool	1 (1)	2 (2)	2 (1)
UC flare	0	0	1 (1)[e]
Food poisoning	0	0	1 (1)

Number in the parenthesis represent subjects experiencing an adverse event (AE).

**The 16 subjects in the placebo group and the 16 subjects in the *AQ-90d group are the same individuals. These subjects crossed over to Aquamin® after 90 days. Adverse events listed under placebo were reported during the first 90 days and adverse events listed under AQ-90d were reported during the final 90 days of intervention. The adverse events reported by the 12 subjects in the AQ-180d group were reported over the entire treatment period.*

[a] *Associated with Covid-19 infection in one subject*

[b] *Could be due to Saxenda, a medication for chronic weight management, taken by this patient*

[c] *Associated with Covid-19 infection in two subjects*

[d] *One of the subjects reporting abdominal discomfort attributed this to being off of his regular UC treatment for 5 weeks due to lack of insurance*

[e] *The flare was detected during the 90-day visit with improvement by the 180-day visit*
