# Supplementary material for: A multi-mineral intervention to improve disease-related and mechanistic biomarkers in ulcerative colitis patients: Results from a randomized trial
Source: PLoS One. 2025 Dec 8;20(12):e0337408. doi: 10.1371/journal.pone.0337408 (PMC12685183; doi:10.1371/journal.pone.0337408)
Supplement: S6 Table — (PDF) [file pone.0337408.s009.pdf]

Supplement Table 6. Subjective Feedback: Response to Aquamin®

Subject ID (Duration)	Participants reported or noted:
Subject 1 (180-d)	feeling better, biologics (Tofacitinib) dose reduced by the GI doc after the study completion
Subject 2 (180-d)	improved energy level - improved - felt fatigued again 2 weeks after completing study
Subject 3 (180-d)	improved energy level - Improved. However, felt fatigued again after discontinuing Aquamin
Subject 4 (180-d)	feeling much better and felt comfortable; otherwise felt that flare could start anytime
Subject 5 (180-d)	improved stool consistency and improved frequency
Subject 6 (180-d)	hairs got better, Participant reported feeling much healthier
Subject 7 (180-d)	feeling good, Participant noted being without UC maintenance therapy due to lack of insurance
Subject 8 (180-d)	"90% on the way to remission" using own words, did complain of some blood in stool
Subject 9 (180-d)	feeling better in general and had better mobility with decreased arthritis issues.
Subject 10 (180-d)	commented, "I loved the treatment and felt great during my participation"
Subject 11 (90-d)	feeling better
Subject 12 (90-d)	feeling fatigued after completing study and noted that joint pains started again
Subject 13 (90-d)	that stool firmed up; Participant noted never having had that consistency of stool in last 20 years
Subject 14 (90-d)	firmer stool compared to pre-participation stage and less reactive to previous dietary triggers
Subject 15 (90-d)	feeling much better; Participant noted a decreased number of days with GI discomfort
Subject 16 (90-d)	feeling energetic and feeling much better as compared to last visit
Subject 17 (90-d)	patient experienced a flare 2 weeks after discontinuing Aquamin, which required a mesalamine suppository
Subject 18 (90-d)	improved stool consistency
Subject 19 (90-d)	mesalamine reduced to half-dose; decreased dysplastic changes in the colon (on GI surveillance)

Nineteen subjects provided additional feedback at the last study visit or at the close-out call two weeks later. For public dissemination, subject identifiers replaced by numbers 1 through 19.

180-d: Aquamin given for 180 days (n=12 subjects). 90-d: Aquamin given for last 90 days (n=16 subjects).
