# Supplementary material for: A multi-mineral intervention to improve disease-related and mechanistic biomarkers in ulcerative colitis patients: Results from a randomized trial
Source: PLoS One. 2025 Dec 8;20(12):e0337408. doi: 10.1371/journal.pone.0337408 (PMC12685183; doi:10.1371/journal.pone.0337408)
Supplement: S1 Text — (PDF) [file pone.0337408.s012.pdf]

AQUAMIN®, A MULTI-MINERAL NATURAL PRODUCT FROM RED MARINE ALGAE, AS AN ADJUVENT INTERVENTION FOR ULCERATIVE COLITIS IN REMISSION

Local protocol HUM00156676

Principal Investigator:
James Varani, Ph.D.
Department of Pathology
University of Michigan
Ann Arbor, MI 48109
734-615-0298
Email: varani@umich.edu

Muhammad N. Aslam, MBBS, M.D.
Co-Investigator

D. Kim Turgeon, MD
Co-Investigator

Christine Bassis, PhD
Co-Investigator

Ananda Sen, Ph.D.
Biostatistician

Previous submission dates and version numbers:
Michigan Medicine, Ann Arbor, MI
Version 1, January 18, 2019

Michigan Medicine, Ann Arbor, MI
Version 2, June 24, 2019

Name(s) of study agent(s):
Aquamin®

IND number/IND holder (if applicable): 141,600/James Varani, Ph.D.

SCHEMA

40 evaluable subjects with stable ulcerative colitis will be enrolled and randomized to Aquamin® (for 180 days) or placebo (for first 90 days and crossover to Aquamin® for the next 90 days)

↓

At Day 0, subject will have a flexible sigmoidoscopy with colon and stool sampling, a blood draw, Dual energy x-ray absorptiometry (DEXA), saliva sampling, morphometric measurements, a physical exam, and vitals. Subject will begin taking 90-day supply of capsules.

↓

At Day 90, subject will have a flexible sigmoidoscopy with colon and stool sampling, a blood draw, saliva sampling, morphometric measurements, a physical exam, and vitals. Subject will begin taking 90-day supply of Aquamin®.

↓

At Day 180, subject will have a flexible sigmoidoscopy with colon and stool sampling, a blood draw, DEXA, saliva sampling, morphometric measurements, a physical exam, and vitals.

↓

Subject will be off study after a final call, two weeks after the completion of Visit #4 and any AEs have resolved.

ABBREVIATIONS

AE	Adverse Events
ALKP	Alkaline phosphatase
ALT	Alanine transaminase
ANOVA	Analysis of Variance
AST	Aspartate Aminotransferase
BMD	Bone Mineral Density
CaSR	Calcium-sensing receptor
CAECAMS	Carcinoembryonic antigen-related cell adhesion molecule
CK20	Cytokeratin 20
COMP	Comprehensive Metabolic Panel
CRA	Clinical research associate
CRF	Case Report Form
CRP	C-reactive protein
CTCAE	Common Terminology Criteria for Adverse Events
DNA	Deoxyribonucleic acid
DSMC	Data Safety and Monitoring Committee
DEXA	Dual energy x-ray absorptiometry
ELISA	Enzyme-linked immunosorbent assay
FDA	Food and Drug Administration
GCP	Good Clinical Practice
GRAS	Generally Recognized As Safe
HACCP	Hazard Analysis and Critical Control Point
IBD	Inflammatory Bowel Disease
IBDQ	Inflammatory Bowel Disease Questionnaire
IHC	Immunohistochemistry
IL-8	Interleukin 8
IND	Investigational New Drug
ISO	International Organization for Standardization
IRB	Institutional Review Board
IRBMED	University of Michigan Medical School Institutional Review Board
LPN	Licensed practical nurse
MA	Medical assistant

MCP-1	Monocyte chemoattractant protein-1
MCRU	Michigan Clinical Research Unit
P1NP	Procollagen type 1 N-terminal propeptide
PERP	p53 apoptosis effector related to PMP-22
PCNA	Proliferating Cell Nuclear Antigen
PI	Principal Investigator
SAE	Serious Adverse Events
SCFA	Short-chain fatty acids
SCR	Scheduled Continuing Review
SIBDQ	Short Inflammatory Bowel Disease Questionnaire
SOP	Standing Operating Procedure
TNF-α	Tumor necrosis factor-α
TRAP 5b	Tartrate-resistant acid phosphatase 5b
UCDAI	Ulcerative Colitis Disease Activity Index
UMHS	University of Michigan Health System
UMRP	University of Michigan Research Pharmacy

TABLE OF CONTENTS

TABLE OF CONTENTS 5
1 OBJECTIVES 8
1.1 PRIMARY AIMS 8
1.2 SECONDARY AIMS 8
2 BACKGROUND AND RATIONALE 9
2.1 ULCERATIVE COLITIS 9
2.2 PATHOGENESIS OF ULCERATIVE COLITIS 10
2.3 MUCOSAL BARRIER 10
2.4 CALCIUM AND IMPROVED DIFFERENTIATION IN THE COLON......11
2.5 COLON CELL DIFFERENTIATION INDUCED BY CALCIUM IN CONJUNCTION WITH OTHER TRACE MINERALS 11
2.6 PRECLINICAL STUDIES WITH AQUAMIN®: THE TEST PRODUCT PROPOSED FOR USE HERE 12
2.7 CLINICAL STUDIES WITH AQUAMIN® IN HEALTHY SUBJECTS 14
2.8 RATIONALE FOR USE OF AQUAMIN® AS AN ADJUVANT THERAPY IN ULCERATIVE COLITIS IN REMISSION 15
2.9 ADDITIONAL ENDPOINTS: RATIONALE FOR AQUAMIN® EFFECT ON BIOMARKERS OF BONE TURNOVER AND ON ORAL MICROBIOTA 16
3 STUDY PLAN SUMMARY 16
3.1 STUDY DESIGN 16
3.2 STUDY OBJECTIVES 16
4 PARTICIPANT SELECTION 17
4.1 STUDY POPULATION 17
4.2 PARTICIPATING CENTERS 17
4.3 SOURCES OR METHODS OF RECRUITMENT 18
4.4 METHOD OF PARTICIPANT NUMBERING 18
4.5 INCLUSION CRITERIA 18
4.6 EXCLUSION CRITERIA 18
4.7 INELIGIBLE, EVALUABLE, AND NON-ADHERENT SUBJECTS...........19
4.7.1 INELIGIBLE SUBJECTS 19
4.7.2 EVALUABLE SUBJECTS 19
4.7.3 NON-ADHERENT SUBJECTS 19
5 AGENT INFORMATION AND ADMINISTRATION 20
5.1 NAME OF AGENT 20
5.2 DOSE AND DURATION OF EXPOSURE 20
5.3 PREPARATION AND FORMULATION 20
5.4 ADMINISTRATION 21
5.5 SIDE EFFECTS 21
5.6 PACKAGING AND LABELS 21
5.7 STORAGE 21
5.8 DISTRIBUTION 21
5.8.1 STRATIFIED RANDOMIZATION 21
5.8.2 BLINDING AND UNBLINDING 22
5.9 DOSE MODIFICATION 22

5.10 ADHERENCE 22
5.11 ORDERING CAPSULES FOR A PARTICIPANT....23
5.12 CAPSULE ACCOUNTABILITY....23
5.13 DRUG DISPOSAL....23
6 CRITERIA FOR EVALUATION AND ENDPOINT DEFINITION....23
6.1 EVALUATION ENDPOINTS 23
6.1.1 PRIMARY OBJECTIVES....23
6.1.2 SECONDARY OBJECTIVES....24
6.2 PROTOCOL ENDPOINT....24
6.2.1 ADDITIONAL STUDIES 24
7 CLINICAL EVALUATIONS/PROCEDURES 26
7.1 SCHEDULE OF EVENTS....26
7.2 VISIT #1-SCREENING VISIT....26
7.2.1 CONCOMITANT MEDICATION AND SUPPLEMENTS 27
7.3 VISIT #2-DAY 0 (BASELINE VISIT)....27
7.3.1 COLLECTION OF STOOL SAMPLES AND COLON TISSUE....28
7.3.2 TOXICITY ASSESSMENT....28
7.3.3 COMPLIANCE AND BLINDING ASSESSMENT 29
7.4 SAFETY MONITORING 29
7.5 VISIT #3- DAY 90 (HALFWAY VISIT) 29
7.6 VISIT #4-DAY 180 (FINAL VISIT)....30
7.7 DOCUMENTATION OF MEDICATION 31
7.8 MEDICATION DURING TRIAL....31
7.9 SCHEDULING ISSUES, MISSED DOSES, EXTENUATING CIRCUMSTANCES 31
7.10 OFF STUDY 31
8 ADVERSE EVENTS 31
8.1 ADVERSE EVENT REPORTING 32
8.1.1 SEVERITY AND GRADING....32
8.1.2 FOLLOW-UP....32
8.1.3 SERIOUS ADVERSE EVENTS (SAE) 33
8.1.4 WITHDRAWAL OF SUBJECT FROM TRIAL AFTER SAE 33
8.2 FREQUENCY OF MONITORING....33
8.3 PLAN FOR REPORTING ADVERSE OR OTHER REPORTABLE EVENTS OR INFORMATION....33
8.3.1 REPORTING TO THE IRBMED....33
8.3.2 REPORTING TO THE FDA....34
9 DOSE/TREATMENT MODIFICATION AND STUDY STOPPING CRITERIA34
9.1 DOSE/TREATMENT MODIFICATION CRITERIA 34
9.2 STUDY STOPPING CRITERIA....35
9.3 PREMATURE REMOVAL OF A PARTICIPANT....35
9.3.1 REASONS FOR PREMATURE WITHDRAWAL FROM THE STUDY35
9.3.1.1 PARTICIPANT WITHDRAWAL 35
9.3.1.2 NON-COMPLIANCE WITH DOSING 35
9.3.1.3 LOST TO FOLLOW-UP 35
9.3.1.4 UNACCEPTABLE ADVERSE EFFECTS 36

9.3.1.5 PI, INVESTIGATOR DISCRETION 36
9.3.2 RECOMMENDATION IN CASE OF PREMATURE WITHDRAWAL FROM THE STUDY 36
10 DATA MANAGEMENT 36
10.1 CASE REPORT FORM SET 36
10.2 DATA ENTRY, DATA MANAGEMENT AND QUALITY CONTROL 36
11 STATISTICAL CONSIDERATIONS 36
11.1 STUDY DESIGN 36
11.2 ANALYSIS PLAN 37
11.2.1 PRIMARY AIMS 37
11.2.2 SECONDARY AIMS 37
11.2.3 SAMPLE SIZE JUSTIFICATION 38
12 ETHICAL AND REGULATORY CONSIDERATIONS 39
12.1 INSTITUTIONAL REVIEW BOARD (IRB) APPROVAL 39
12.2 INFORMED CONSENT 39
12.3 DATA AND SAFETY MONITORING PLAN 39
12.4 REGULATORY AGENCY MONITORING 39
12.5 RECORD RETENTION 39
12.6 DATA SAFETY AND MONITORING 40
12.6.1 AUTHORITY AND DUTIES 40
12.6.2 COMPOSITION 40
12.6.3 MEETING FREQUENCY 40
12.6.4 RECOMMENDATIONS AND REPORTING 41
13 REFERENCES 42
APPENDIX 1: CERTIFICATE OF ANALYSIS OF AQUAMIN® 48
APPENDIX 2: MINERAL COMPOSITION OF A TYPICAL BATCH OF AQUAMIN® 50
APPENDIX 3: ADVERSE EVENTS (AES) 55
ADVERSE EVENTS IN A SIMILAR AQUAMIN® STUDY 55
ADVERSE EVENTS IN AQUAMIN® PILOT STUDY (HUM00076276) 55
SERUM BIOMARKERS IN AQUAMIN® PILOT STUDY (HUM00076276) 56
APPENDIX 4: DETAILED PROCEDURES FOR UNPREPPED FLEXIBLE SIGMOIDOSCOPY 57
APPENDIX 5: TISSUE/STOOL SOP 58
APPENDIX 6: BLOOD SOP 62
APPENDIX 7: SALIVA SOP 63
APPENDIX 8: ASSESSMENT OF ADHERENCE WITH AGENT 64
APPENDIX 9: IND CLINICAL TRIAL MONITORING PLAN 65
APPENDIX 10: DATA SAFETY AND MONITORING PLAN 67
DATA SAFETY MONITORING COMMITTEE 67
ADVERSE EVENT DEFINITIONS AND REPORTING 70

1 OBJECTIVES

1.1 Primary Aims

1. To evaluate whether Aquamin® taken daily, standardized to 800 mg of calcium per day for 90 and 180 days, will improve clinical findings (reduced symptomatology and decreased frequency of flare-ups) and/or laboratory findings in subjects with ulcerative colitis in remission. Clinical assessment will include a questionnaire (IBDQ) and a gastroenterologist's endoscopic findings based on Mayo Score (UCDAI). Laboratory findings will include histological evaluation and levels of fecal calprotectin and serum C reactive protein (CRP).
2. To evaluate whether Aquamin® taken daily, standardized to 800 mg of calcium per day for 90 and 180 days, will improve defined mucosal markers of differentiation and barrier function and decrease mucosal inflammation (determined by histological assessment) and tissue IL-8 measurement as compared to placebo.

   Hypothesis: Aquamin® taken daily, standardized to 800 mg of calcium at the proposed schedule, will increase markers of differentiation and barrier function in colon tissue and decrease mucosal inflammation compared to placebo treatment. These changes will be associated with improved clinical and/or laboratory findings of ulcerative colitis in subjects with ulcerative colitis in remission

1.2 Secondary Aims

1. To evaluate whether Aquamin® taken daily for 90 and 180 days, standardized to 800 mg of calcium per day, will alter the oral and gut microbiota, and lead to changes in the bile acids profile, short-chain fatty acids (SCFA) and eicosanoids versus placebo.

   Hypothesis: Aquamin® taken daily, standardized to 800 mg of calcium at the proposed schedule, will alter oral / gut microbiota and alter metabolomic profile.
2. To evaluate whether Aquamin® taken daily for 90 and 180 days, standardized to 800 mg of calcium per day will alter levels of liver enzymes (ALT, AST and ALKP) and bone turnover markers in serum versus placebo.

   Hypothesis: Aquamin® taken daily, standardized to 800 mg of calcium at the proposed schedule, will not alter levels of liver enzymes (ALT, AST and ALKP) but will improve bone markers as compared to placebo.

2 BACKGROUND AND RATIONALE

2.1 Ulcerative Colitis

Ulcerative colitis (UC) is a chronic disease that manifests as diffuse mucosal inflammation and superficial ulcers on the inner lining of the large intestine. Since the mucosal layer of the gastrointestinal tract plays an important role in host innate defense mechanisms, regulates secretion, and absorption, and maintains a barrier to bacteria, bacterial products, toxins and food allergens, anything that compromises the integrity of the protective barrier in the large intestine affects the well-being of the host (1,2).

Ulcerative colitis is common. Approximately 1.6 million Americans have a diagnosis of inflammatory bowel disease. About 907,000 of them having ulcerative colitis; a prevalence rate of approximately 238 per 100,000 adults. Between 2 and 14 people are diagnosed with ulcerative colitis per 100,000 person-years (3,4). Ulcerative colitis is slightly more common in males. Risk factors include age, race or ethnicity and family history. The disease usually begins before the age of 30, but it can occur later in the life. This chronic, lifelong condition can be treated but not cured, indicating the need for more research.

Ulcerative colitis can begin fulminantly or gradually and become worse over time. Symptoms can be mild to severe. Most people have periods of remission - times when symptoms disappear - that can last for weeks or years. The course of the disease is highly variable from person to person, with some people having long periods of remission. The goal of therapy is to keep people in remission for as much of the time as possible. Ulcerative colitis can be debilitating and can sometimes lead to life-threatening complications.

Ulcerative colitis symptoms can vary, depending on the severity of disease and location. Signs and symptoms may include: diarrhea (often with blood or pus), abdominal pain and cramping, rectal pain, urgency to defecate, loose stools, weight loss, fatigue, and fever. In children, it also may lead to failure to grow. Ulcerative colitis patients are recommended to have more-frequent screening for colon cancer because of increased risk for colorectal cancer (5,6).

Several categories of drugs may be effective in treating ulcerative colitis. Typically, these medications do not completely eliminate the disease but can reduce symptoms. The goals of therapy are to induce and maintain remission and improve quality of life. Treatment depends on the severity of disease, patient response and the risk of side effects from treatment. Anti-inflammatory drugs are often the first step in the treatment of ulcerative colitis. Examples of anti-inflammatory agents include 5-aminosalicylates (sulfasalazine, mesalamine, and olsalazine) and corticosteroids (prednisone and hydrocortisone). The potential for side effects limits long-term steroid use.

Immune system suppressor drugs also reduce inflammation, but do so by suppressing the immune system response that regulates inflammation. The most commonly used immunosuppressant drugs include Azathioprine, Cyclosporine and Mercaptopurine. Another, newer, class of immune regulatory drugs are biologicals – agents that target specific proteins such as tumor necrosis factor-α (TNF-α) and other cytokines that regulate the inflammatory response. The (potential) benefit of these agents is more precise control than can often be

attained with broad-acting agents such as corticosteroids. If all medical treatment fails, or the course is fulminant and unrelenting (e.g., severe bleeding, toxic megacolon), surgery may be an appropriate solution (7).

Lifestyle and dietary changes are often recommended to help control the symptoms and lengthen the time between the flare ups. Limiting dairy products, fiber, spicy food, alcohol and caffeine may be helpful, especially during the flare up. Many people with digestive disorders have used some form of complementary and alternative therapy to either lessen the symptoms or lengthen the "in remission" period. Of potential value in this regard is the long-term use of oral agents including nutritional supplements and botanicals. Dietary components and nutraceuticals can favorably alter barrier function and apoptosis. Furthermore, due to the unique location of the target cells in the colon, it is possible to introduce agents that function in a "topical" fashion rather than systemically.

2.2 Pathogenesis of Ulcerative Colitis

The exact cause of ulcerative colitis is unknown. Evidence indicates that both genetic and environmental factors contribute. While there is no specific gene that has yet been indisputably linked to ulcerative colitis, the most consistent genetic associations have been shown for the MHC locus HLA Class II alleles. The interleukin-1 family of genes and the multidrug resistance gene MDR1 have also been linked as genetic susceptibility factors for the development of ulcerative colitis (8,9). From an environmental standpoint, there is a relationship between ulcerative colitis and bacterial flora, with an increased number of adherent *Bacteroides spp*. and *Enterobacteriaceae spp.* often present in inflamed bowel segments. The functional activity of these microbial strains may lead to 'dysbiosis' and affect the metabolic activity of colonocytes or enterocytes, leading to the development of ulcerative colitis (10-12). There is evidence of some oral manifestations (halitosis and oral ulceration) associated with ulcerative colitis (13). Additionally, oral microbial flora has implication in the disease pathogenesis and can be a useful parameter to assess the disease status by sampling saliva (14). From an immunologic standpoint, ulcerative colitis has less of a T helper type 1 (Th1) cytokine response pattern than Crohn's disease (15). Recent studies have indicated that in contrast to Th1 cytokines that are associated with Crohn's disease (Interferon-γ, TNF-α and interleukin-12), animal models of ulcerative colitis may be associated with increased natural-killer-cell activity and interleukin-13 (16). In addition, attention is being directed at the possible down regulatory role of transforming growth factor-ß in ulcerative colitis and the possibility that defective signaling of transforming growth factor-ß may account for inadequate tissue repair (15). Despite our improved understanding of the genetics and environmental factors that underpin this disease, the etiology and pathogenesis of ulcerative colitis remain largely undefined.

2.3 Mucosal barrier

While ulcerative colitis is an inflammatory disease, there is sound reason to suggest that a defect in the colon mucosal barrier plays a role in disease initiation. There is, for example, an association between ulcerative colitis and genes that are relevant to the mucosal barrier (17). In another study, increased intestinal permeability was shown in unaffected relatives of ulcerative colitis patients and increased immune reactivity against bacterial antigens was noted (18). Colonic reduction of mucin granules and a thinner mucous layer have been reported with

increased bacterial penetration in active ulcerative colitis lesions (19) and increased mucosa-associated bacteria in children with inflammatory bowel disease has been reported (20). Importantly, direct evidence that barrier defects and bacterial dysbiosis are inflammation-independent hallmarks of ulcerative colitis has been reported (18-21). Finally, mucosal barrier damage in animal models produces inflammatory changes in the gut (22). It is in the context of a defective barrier in the colon (and the potential for its repair by a calcium-rich, multi-mineral natural product) that the proposed trial is based.

2.4 Calcium and improved differentiation in the colon

Compelling epidemiological evidence indicates that calcium intake and the formation of precancerous colonic polyps are inversely correlated (23-25), and a recent meta-analysis suggests that the correlation extends to colon cancer, itself (26). How calcium intake reduces abnormal epithelial growth in the colonic epithelium is not fully understood. What is known, however, is that calcium is the quintessential inducer of epithelial cell differentiation. In monolayer cell culture, calcium induces gene expression associated with differentiation including P27(Kip1), P21(WAF1), cytokeratins recognized by the AE1/AE3 anti-keratin antibody combination, CK20 and E-cadherin (27-34). In the colon, specifically, calcium promotes E-cadherin synthesis and a dramatic increase in cell surface expression. When E-cadherin is present in the cell membrane, cytosolic β-catenin binds to the cytoplasmic tail of E-cadherin and connects the cell surface to the actin cytoskeleton. Sequestration of β-catenin in the cell surface-cytoskeletal complex reduces β-catenin translocation to the nucleus and reduces growth-promoting TCF4 / Wnt signaling (27-34). While this understanding of calcium's role in epithelial growth regulation is well-established in the literature, our laboratory's contribution has been to show that a protein known as the extracellular calcium-sensing receptor (CaSR) is a critical mediator of calcium-generated pro-differentiating and growth-regulating signals in the human colonic mucosa (reviewed in 35-38).

It should be noted that regardless of the role of epithelial differentiation in colon polyp prevention, there is no question that improved differentiation is a prerequisite to effective barrier formation and tissue integrity. Thus, attempting to mitigate ulcerative colitis with calcium should be seen independently of calcium effects on polyp formation.

2.5 Colon cell differentiation induced by calcium in conjunction with other trace minerals

While calcium is critical to epithelial differentiation, a number of other monovalent, divalent and trivalent cationic metals may also be important (39,40). In some cases, the functions are independent of calcium. For example, certain transition metals including copper, zinc, manganese and selenium are components of anti-oxidant enzymes (41). These metals, therefore, affect the formation of oxidant-driven mutations that may cause dis-regulation of differentiation-regulating signaling pathway. Another group of transition cationic metals, the lanthanide metals, may be particularly important in modulating the growth-regulating and differentiation activity of calcium. The lanthanide elements are similar in ionic radius and orbital configuration to calcium, but have higher overall charge density. Several of the lanthanide metals bind to calcium-binding sites on various regulatory proteins, including CaSR. In some cases, the affinity is higher than that of calcium itself (42). Lanthanide metals trigger events normally mediated by

calcium and modulate the effects of calcium on these same events. Our own past studies have demonstrated that lanthanide elements activate CaSR in colon epithelial cells (38) and lower the concentration of calcium needed for effective growth control (43). Of interest, the same lanthanides that interact with calcium to modulate growth and differentiation in epithelial cells also stimulate fibroblast growth (44). Thus, there is no evidence that the epithelial responses are a reflection of non-specific cytotoxicity.

2.6 Preclinical studies with Aquamin®: the test product proposed for use here

Given the importance of adequate calcium intake to epithelial cell differentiation and the potential for additional trace elements to collaborate with calcium in its epithelial-differentiating activity, we began a series of studies with a natural product available under the trade-name Aquamin®. Aquamin® is a calcium-rich, multi-mineral material derived from the skeletal remains of red marine algae, *Lithothamnion sp* (45). The red marine algae thrive in the shallow Atlantic waters off the southwest coast of Ireland and northwest coast of Iceland and accumulate minerals from the ocean water over its lifespan. Eventually, the mineral-rich fronds break off of the living algae and fall to the ocean floor. The fronds are harvested from the ocean floor, separated from extraneous materials (prepared in order to remove all organic material), and sterilized, dried, and milled under ISO and HACCP certification. The natural product contains high levels of calcium and magnesium, but in addition, contains measurable levels of 72 different trace minerals. Aquamin is sold as a dietary supplement (GRAS 000028) (Marigot, Ltd; Cork, Ireland) and is used in various products for human consumption in Europe, Asia, Australia, and North America.

Cell culture studies. The underlying assumption for our initial studies was that individual metals (or combination of metals) in the mineral-rich natural product might provoke a response under conditions in which calcium alone was ineffective. To test this hypothesis, we compared calcium alone with the multi-mineral natural product for effects on growth and differentiation in several human colon carcinoma cell lines and in cells selected from the same parent lines for resistance to calcium (38,46). The mineral-rich algae product was similar to calcium alone in its effects on the calcium-responsive (parental) cells. However, cells non-responsive to calcium alone were still responsive to the combination of calcium and the other minerals in Aquamin® as demonstrated by increased differentiation and reduced proliferation. These findings suggest that resistance to calcium-mediated growth regulation does not imply, *a priori*, resistance to other interventions (46). As such, a multi-mineral supplement may provide better growth control than calcium alone across a range of colonic epithelial cells, and be particularly effective in cells that have reduced calcium-sensitivity.

Animal model. To assess *in vivo* effects, we next conducted two separate studies in healthy (C57Bl/6) mice maintained for 15-18 months on either a normal rodent chow or a high-fat modification of the same rodent chow. The high-fat diet was initially developed to mimic the diet typically consumed by many individuals in Western countries (47,48). In addition to increased saturated fat, the high-fat diet contained several other features. Among these were replacement of methionine with cysteine and reductions in choline, folate, total fiber, and elemental calcium. In our studies, animals maintained on a rodent chow diet (with calcium), had a colon polyp incidence of 18% (16 of 90 mice). In animals fed a Western-style diet (low calcium) without the mineral supplement, the incidence of polyp formation was 29% (26 of 90)

while in littermates fed the Western-style diet with Aquamin®, the incidence was 2% (2 of 90) (49,50). Of note, when the tumors were examined histologically, several of the lesions in the Western diet-fed mice proved to be invasive carcinomas. No invasive tumors were seen in mice fed the Aquamin®-supplemented Western-style diet. These data, suggest that supplementation may affect tumor progression as well as tumor formation

The reduction in polyp formation may be a direct consequence of a mineral effect on epithelial cell proliferation, but could also reflect improved epithelial differentiation / barrier function and, concomitantly, reduced inflammation in the colon. We suggest this because in addition to reducing colon polyp formation, mice receiving Aquamin® in the diet developed fewer liver tumors (51), maintained bone mineral content (52,53) and had fewer and less severe episodes of ulcerative skin lesions (54) than did animals on the same diet without Aquamin®. These effects cannot readily be attributed to a direct effect on colon epithelial cell proliferation. Furthermore, it was noted that animals on the high-fat diet without mineral supplementation had evidence of chronic inflammation throughout the gastrointestinal tract (49) and this was mitigated with Aquamin®.

Enteroid culture studies. We have utilized recently-developed enteroid culture technology - i.e., 3-Dimensional culture of intact human colonic tissue (55-57) - to compare Aquamin® with calcium alone for effects on proliferation and differentiation in human colon adenomas (56). Supplementation of the culture medium with either calcium alone or Aquamin® reduced proliferation (Ki67 expression). Concomitantly, treatment with either agent induced morphological features consistent with differentiation and up-regulated multiple differentiation-associated proteins as seen in immunohistochemistry (IHC) and in a proteomic screen. Barrier protein (occludin) expression was increased and transmission electron microscopy revealed abundant tight junctures and desmosomes in the treated tissue. These features of differentiation were observed at a lower concentration of Aquamin® than seen with calcium alone.

In subsequent studies, we established enteroid cultures from histologically-normal colonic mucosa and examined responses to the same interventions. Unlike what was observed in tumor tissue (56), histologically normal tissue underwent differentiation under low-calcium control conditions over a 5-7 day period. Calcium alone and Aquamin® did not alter this substantially. Of interest, however, in spite of the lack of morphological changes with supplementation, we observed a dramatic increase in the production of several proteins contributing to cell-cell adhesion, cell-matrix adhesion and barrier formation. Among these were cadherins (*adherens* junctions), claudins (tight junctions), desmosomal proteins (desmocolin-2, desmoglein-2, desmoplakin and PERP) as well as several CAECAMS and laminin chains (cell-substrate adhesion). Transmission electron microscopy revealed the presence of large numbers of desmosomes in the Aquamin®-treated tissue while such structures were sparse in tissue maintained under control conditions. Thus, although the interventions did not have a major impact on overall enteroid morphology, these data certainly suggest improvement in barrier function / tissue integrity. These studies with histologically-normal tissue are currently "under review" (57).

Enteroid culture studies with colonic tissue from individuals with ulcerative colitis. Using the same enteroid culture technology, we were able to establish enteroid cultures from colonic mucosal biopsies obtained from six subjects with ulcerative colitis in remission. We also got

colonic tissue from two subject with active disease. Once cultures were established, we compared Aquamin® to placebo for ability to i) improve biomarkers of differentiation and barrier formation, and ii) reduce biomarkers of chronic inflammatory changes in the growing enteroid cultures. As compared to tissue from healthy subjects, colonic biopsies from inflammatory bowel lesions adapted more slowly to enteroid culture. After establishment in enteroid culture, however, ulcerative colitis tissue grew well in culture and underwent differentiation in response to Aquamin® treatment (similar to what we have seen in tissue from control subjects). While this work is still in progress, our findings to date indicate greater desmosome density in Aquamin®-treated enteroids than in controls. Also of interest, we assessed a panel of pro-inflammatory cytokines in the enteroid tissue from ulcerative colitis subjects and found that while there was little detectable IL-1β, TNF-α, IL-6 and MCP-1, a substantial level of IL-8 was found in all samples. IL-8 levels were reduced in Aquamin®-treated samples compared to control. Since IL-8 is thought to be one of the major chemotactic factors for neutrophil accumulation in ulcerative colitis lesions (58), its reduction with Aquamin® treatment is suggestive of a beneficial effect.

2.7 Clinical studies with Aquamin® in healthy subjects

We have recently completed the interventional phase of a pilot biomarker study in healthy subjects at increased risk for colon cancer based on a personal history of colon polyps and / or a family history of colon cancer. The analysis phase is ongoing. In this study, which is being conducted under IND #118194 granted to Dr. James Varani, 30 subjects (10 per arm) were treated daily for 90-days with Aquamin® (approximately 2400 mg of Aquamin®; an amount designed to provide 800 mg of calcium per day), or with calcium carbonate (800 mg of calcium per day) or with maltodextrin as a placebo. Prior to intervention and at the end of the treatment period, subjects underwent flexible sigmoidoscopy and a series of colonic biopsies and fecal specimens (from within the colon) were obtained. A variety of biomarkers are being assessed in these specimens. In parallel, we obtained measures of safety and tolerability. While the analysis is still ongoing, we can report the following;

1st. There appeared to be no safety or tolerability issues with Aquamin®. This is consistent with what has been reported in the past by other investigators (59,60). Appendix 3 summarizes adverse events occurring in subjects consuming Aquamin® in past trials and includes a table of pre- post- liver enzyme levels from our ongoing trial.

2nd. Aquamin® up-regulated and down-regulated numerous proteins in colonic tissue biopsies as compared to biopsies from placebo-treated subjects. Treatment with calcium alone also affected the levels of many proteins.

3rd. Aquamin® treatment for 90 days altered the microbial population of the gastrointestinal tract as compared to placebo. This was observed in both the fecal specimens and colon tissue biopsies. Interestingly, we saw no significant difference between calcium alone and placebo. The gut microbial changes with Aquamin® were accompanied by a reduction in bile acid levels and a tendency toward increase in levels of several short chain fatty acids. These changes were not observed with calcium alone. The findings from this study have been published in abstract form (61) after presenting at Pathobiology for Investigators, Students, and Academicians (PISA) 2018 meeting. These data were also presented orally at the Annual Meeting of the Society for Integrative Oncology (October 27, 2018; Phoenix, AZ).

4th. We are currently assessing expression levels for several proliferation and differentiation markers by IHC / proteomics. Not enough findings are yet available to indicate what the outcome will be.

The study proposed below will closely follow the protocol of our existing biomarker trial. The major difference will be in the study population.

2.8 Rationale for use of Aquamin® as an adjuvant therapy in ulcerative colitis in remission

Long term goal. The long-term goal of this study is to determine the value of the multi-mineral supplement (Aquamin®) as an adjunct therapy for individuals with ulcerative colitis who are currently in stable remission. The use of Aquamin® will (hopefully) improve symptomatology and prolong the remission period. If this can be achieved, the long-term use of Aquamin® should improve the quality of life of individuals with ulcerative colitis.

Working hypothesis. The key feature of our hypothesis is that Aquamin® will promote differentiation in the colonic mucosa. The main consequence of Aquamin®-induced differentiation will be an improved epithelial barrier and improved tissue cohesion. Defective barrier function and poor tissue cohesion in the gastrointestinal tract and chronic inflammation go "hand in hand". Commonly, it is thought that chronic inflammation is responsible for tissue destruction and reduced tissue cohesion. However, it is likely that poor barrier function and poor tissue cohesion contributes to inflammation. In the absence of an effective barrier, bacteria, bacterial products, toxins and food allergens can all gain access to the interstitium. Inflammation in the gastro-intestinal tract and ulcerative colitis symptomatology are linked. Improved barrier function / tissue cohesion should lead to decreased inflammation and, therefore, contribute to decreased symptoms and improved colonic health. Additionally, our recent Aquamin® trial also suggests that Aquamin® may have a role as a prebiotic and has the potential to improve colonic health by altering gut microbiome and microbially-derived metabolites (bile acids and short chain fatty acids) (61, and manuscript in preparation). Taken together, this summary of past studies presented above supports our working hypothesis.

Proposed studies. In the study outlined here, we plan to enroll 40 evaluable subjects with ulcerative colitis in remission in a two-arm protocol (placebo versus Aquamin®). Twenty individuals will receive daily Aquamin® for 180 days (approximately 2400 mg of Aquamin®; an amount designed to provide 800 mg of calcium per day). The other twenty subjects will be treated for 90-days with the placebo (maltodextrin). After the initial 90-day treatment, control subjects will be crossed over and treated with Aquamin® for the last 90 days. Prior to intervention, at the 90-day time-point and at the end of the treatment period, we will use clinical and laboratory findings to determine if Aquamin® has had an impact on disease parameters. Additionally, subjects will undergo an unprepped flexible sigmoidoscopy at all three time points and a series of colonic biopsies and fecal specimens (from within the colon) will be obtained. Salivary samples will also be collected at each time point. A variety of biomarkers will be assessed in these specimens. A dual energy x-ray absorptiometry (DEXA) scan will be performed at baseline (Day 0) and the final visit (Day 180) to assess for bone density changes. Finally, we will obtain measures of safety and tolerability.

This initial study will, hopefully, i) demonstrate safety and tolerability of the intervention, ii) provide an indication of efficacy and iii) demonstrate biomarker changes that suggest mechanisms of action.

2.9 Additional endpoints: Rationale for Aquamin® effect on biomarkers of bone turnover and on oral microbiota

Although the primary aim of the study is to determine if Aquamin® improves the clinical and laboratory findings of ulcerative colitis, and fosters improvement in epithelial barrier function / tissue integrity in the colonic mucosa, we are also interested in determining if Aquamin® modifies serum biomarkers of bone metabolism. Inflammatory bowel disease has been associated with osteoporosis and osteopenia. The etiology of osteoporosis in inflammatory bowel disease is multifactorial, with risk factors including age, corticosteroid use, malnutrition, vitamin D and calcium malabsorption and deficiency, immobilization, and the generalized inflammation (62). Inflammatory bowel disease-related nutritional deficiencies have been implicated as other pathogenic mechanisms resulting in low bone mineral density (63). In the past studies, Vitamin D deficiency has been reported more frequently in patients with ulcerative colitis and Crohn's disease as compared with either control subjects or a healthy population reference range. In addition, our past animal studies demonstrating benefit of Aquamin® against colon polyp formation (49,50) also found reduced bone mineral loss over time. Serum biomarkers of bone turnover including P1NP and TRAP-5b (52,53,64) were modulated in parallel by Aquamin-supplementation. Thus, we believe it of value to assess serum biomarkers of bone turnover in our ulcerative colitis subjects receiving Aquamin® or placebo.

Finally, recent studies have also linked ulcerative colitis with oral microbiome changes (13-14). It would be of value, therefore, to see how the oral microbiota is altered in response to Aquamin® in parallel with any changes observed in gut microbiota.

3 STUDY PLAN SUMMARY

3.1 Study Design

This pilot study includes individuals with ulcerative colitis in stable remission, and is a randomized, double-blind, placebo-controlled trial with two parallel treatment groups for the first 90 days. Subjects on placebo will be crossed over to Aquamin® after the halfway visit at day 90. Subjects on Aquamin® will continue their treatment till the end:

1. Aquamin® (2400 mg/day, containing approximately 800 mg calcium/day) – 4 capsules; 2 to be taken in the morning and 2 in the evening.

2. Placebo (Maltodextrin) – 4 capsules; 2 to be taken in the morning and 2 in the evening.

3.2 Study Objectives

The objectives of this study are to characterize the effects of the oral consumption of Aquamin® twice daily for 180 days on clinical and/or laboratory features of ulcerative colitis. As part of clinical endpoint, clinical and endoscopic assessment (based on Mayo Score-UCDAI) will be

carried out by a gastroenterologist (Dr. Kim Turgeon) on Baseline, Intermediate and Final Visits to assess clinical remission. Additionally, histological assessment will compare microscopic features at day 90 and day 180 with those seen prior to the start of treatment (baseline). For this, one of the biopsy specimens will be fixed in formalin and processed for light microscopy. Hematoxylin and eosin-stained slides will be evaluated for crypt structure. With ulcerative colitis patients in remission, one typically sees crypts with distorted architecture and a low (but often detectable) level of inflammation in the *lamina propria*. Plasma cells tend to be highly expressed. Neutrophils can also be seen if there is any active inflammation. A paneth cell hyperplasia extending into the left-side colon and rectum can also be seen on occasion. Fecal Calprotectin and serum CRP levels will also be measured for clinical assessment. Clinical assessment will be complemented with IBDQ scoring and endoscopic findings. We will also assess biomarkers of differentiation and barrier function / tissue cohesion in the colon (quantitative immunohistology and proteomics) and will assess IL-8 by ELISA. We will also assess the effects of Aquamin® on gut microbial communities and metabolomic profile. We will also obtain a saliva sample for oral microbiome evaluation. Bone health will be assessed by measuring bone mineral density (BMD) using DEXA scan and will be correlated with serum bone markers.

Participants will first give a saliva sample, and undergo a baseline blood draw and a flexible sigmoidoscopy where colonic biopsies and fecal specimens will be obtained. Participants will then be randomized to one of two groups: Aquamin® (2400 mg/day, containing approximately 800 mg calcium/day); or placebo (maltodextrin) daily. After 90 days, subjects will give another saliva sample, and undergo another blood draw and a second flexible sigmoidoscopy with biopsies and fecal sample collection. At this point, subjects will be assessed for safety of the drug. If there is no issue regarding the safety, then these subjects will continue the same regimen for the subsequent 90 days. Additionally, subjects on placebo will be crossed over to Aquamin® for the last 90 days. After the second 90-day period, subjects will undergo another blood draw, a salivary sample and a third flexible sigmoidoscopy with biopsies / fecal sample collection. The analysis at day-180 will be the same as at baseline and day-90. At baseline and day-180 visits, subjects will also go through the DEXA scanning procedure.

4 PARTICIPANT SELECTION

4.1 Study Population

Our study population will be comprised of volunteers both male and female, ages 18 - 80 years, of any ethnicity, who have stable ulcerative colitis (are in remission) as determined during screening of personal medical history.

4.2 Participating Centers

All studies will be conducted at the University of Michigan Health System (UMHS) Michigan Clinical Research Unit (MCRU). The MCRU is located in the University Hospital complex, ensuring quick access to any needed medical services.

4.3 Sources or Methods of Recruitment

A coordinator (member of the study team) will identify subjects scheduled in GI clinics or scheduled for colonoscopy that meet the eligibility criteria by review of MiChart. We may also use I2B2 (Honest Broker's Office) to identify potentially eligible subjects. Potentially eligible subjects may be approached at clinic visits or be sent a letter about the study. Volunteers will also be recruited through UMClinicaltrials.org, flyers posted around the University of Michigan including health system clinics, using Data Direct to find potential participants and through word of mouth. Letters may also be sent to previous IBD study participants who indicted interest in future IBD studies and provided permission for additional contact. Individuals who are interested in participating will be scheduled for a Screening Visit. At the Screening Visit, each potential participant will receive a pre-study evaluation.

4.4 Method of Participant Numbering

Each participant will be assigned a unique subject identification number by the study team that will be used on all study forms and specimens. This number will be assigned at the time of enrollment into the study at the Baseline Visit.

4.5 Inclusion Criteria

1. Must be able to give written informed consent.

2. Male or female, ages 18 to 80 years old.

3. Must have the following:

   Ulcerative colitis with confirmed diagnosis by histology and endoscopy; AND be in stable remission for 3 months or more without therapy or with a maintenance therapy (except steroids and antibiotics for 3 months). Note: Corticosteroids (a type of steroid drug such as prednisone or cortisol that helps the body to regulate stress response, immune response and inflammation) and antibiotics can be used during a flare-up once the study has begun and the subject enrolled.

4. A negative pregnancy test for pre-menopausal women with intact female reproductive organs. The negative pregnancy test must be within 2 weeks of the baseline flexible sigmoidoscopy, and subject must agree to use appropriate birth control over the study period. Post-menopausal is defined as no menses for the previous 12 months. If cessation of menses is within 12 months, then the subject should be treated as pre-menopausal and a pregnancy test performed.

4.6 Exclusion Criteria

1. Must not be pregnant or lactating; and women of child bearing potential unwilling to use acceptable birth control throughout the study.

2. Must not be participating in any other interventional trial using an investigational drug.

3. Subjects likely to be uncooperative or unable to comply with study procedures

4. Participants must not be felt to have active ulcerative colitis for 3 months before study enrollment.

5. Participants must not have a history or diagnosis of any of the following conditions:
   i. Crohn's disease.
   ii. Any stomach or intestinal bleeding disorders (gastrointestinal bleeding from gastric or duodenal ulcers, or gastrin secreting tumors) or active gastric / duodenal ulcers - peptic ulcer disease (without bleeding in last 3 months).
   iii. Any gastrointestinal or colonic malignancy.
   iv. Kidney disease, including kidney "stones" or hypercalcemia.
   v. Coagulopathy/hereditary hemorrhagic disorders/ or receiving therapeutic doses of Coumadin or heparin.

6. Participants will be excluded if they have taken the following, within the last 30 days or are unwilling to forgo the following for 30 days prior to entry into the study:
   - Calcium, Vitamin D, including multivitamins that have low amounts of calcium/Vitamin D and fiber supplements.
   - Non-steroidal anti-inflammatory medications (NSAIDs), such as Naproxen or Ibuprofen (except for occasional pain control or low dose aspirin for cardiovascular disease prevention). Note – IBD patients are cautioned to avoid NSAIDs in general since they are associated with flare of disease.

4.7 Ineligible, evaluable, and non-adherent subjects

4.7.1 Ineligible Subjects

Ineligible subjects are those who come to a screening visit, sign the informed consent, and are not eligible to continue in the study. Ineligible subjects will be replaced to get to the study total of 40 evaluable subjects

4.7.2 Evaluable subjects

Evaluable subjects are those who complete the study visits, take at least 75% of their dose, and complete all three flexible sigmoidoscopies. Subjects who are "not evaluable" can be replaced on study to get to the study total of 40 evaluable subjects.

4.7.3 Non-adherent subjects

Subjects who are not adherent with the study protocol (including those not keeping their calendars up-to-date; those not taking the number of pills required on a consistent schedule, or those that are no-shows for scheduled visits) can be taken off study at the PI's discretion, even if they don't want to go off-study. Since visits are paid on a pro-rated basis, subjects will receive compensation based on the visits they complete.

5 AGENT INFORMATION AND ADMINISTRATION

5.1 Name of Agent

Participants will be randomized to receive one of the two agents for the first 90 days. For the second 90 days, all subjects will receive Aquamin®:

i) Aquamin® (2400 mg/day, containing approximately 800 mg calcium/day) – 4 capsules; 2 to be taken in the morning and 2 in the evening.

ii) Placebo (Maltodextrin) – 4 capsules; 2 to be taken in the morning and 2 in the evening.

Aquamin® is the trade name for a natural product that consists of the skeletal remains of the red marine algae, *Lithothamnion sp*., also known as *Phymatolithon sp* (Pallas) (45). The algae thrive in the cold Atlantic waters off the southwest coast of Ireland and northwest coast of Iceland. Minerals from sea water are accumulated in the algae fronds over the lifespan of the organism. Eventually, the mineralized fronds break off of the living organism and fall to the ocean floor, from where they are harvested. The mineralized fronds are separated from extraneous materials, sterilized, dried, and milled under ISO and HACCP certification. The final powdered product contains approximately 32% calcium, 2.5% magnesium, and measurable levels of 72 other trace minerals. The product is sold as a food supplement under the name Aquamin® (GRAS 000028) and is used in various products for human consumption in Europe, Asia, Australia, and North America [Marigot Ltd, Cork, IR]. The certificate of analysis of Aquamin® is presented in Appendix 1. The detailed mineral composition of a typical lot of the algae product (Aquamin®) is presented in Appendix 2.

5.2 Dose and Duration of Exposure

All participants will receive enough capsules for daily-doses of the randomly-assigned study agent for the first 90 days at Visit #2 and enough capsules for daily-doses of Aquamin® for the second 90 days (participants can come within ± 5 days of 90- or 180-day study period; Day 85 to 95 or Day 175 to 185).

5.3 Preparation and formulation

Aquamin® capsules will be provided by Marigot (Cork, Ireland). Marigot will also be responsible for providing the placebo capsules. Contact information is as follows:

Denise O'Gorman, Ph.D.

R&D Manager

Marigot Ltd

Strand Farm

Currabinny

Carrigaline

Co Cork

IRELAND

Email: denise.ogorman@marigot.ie
Tel: +353 (0)21 4378727
Fax: +353 (0)21 438588
Web: www.aquamin.org

5.4 Administration

Capsules will be self-administered by the participant with as much water or other beverage as the participant needs to ingest all the capsules. Participants can ingest capsules with or without food as appropriate (left up to the individual). It is generally recommended that the capsules are taken with food to minimize any digestive upset.

5.5 Side Effects

Side effects may include increased minor gastrointestinal complaints including belching, dyspepsia, and nausea. Please see Appendix 3A for a list of adverse events from a previous study with Aquamin® taken under virtually identical conditions to the conditions proposed here (59-61). In our own recently finished similar trial in subjects at risk for colorectal cancer for 90 days and Aquamin® was well tolerable and no serious adverse events were reported over the period of 90 days (Appendix 3B). Additionally, Aquamin® did not alter levels of serum liver enzymes in subjects who ingested Aquamin® for 90 days (Appendix 3C).

5.6 Packaging and Labels

All study medication will be labelled with study participant's name, ID number, date, protocol number, dosing instructions, and the University of Michigan Research Pharmacy (UMRP) contact information. These tasks will be handled by the UMRP.

5.7 Storage

The capsule shells are made of hydroxypropyl methylcellulose (HPMC). They are vegan/coeliac friendly. HPMC Capsules are fast dissolving in the stomach and easily digestible. HPMC is derived from vegetable cellulose and is 100% natural. These capsules contain no preservatives, gelatin, wheat, gluten, animal by-products or starch, and they are made from pure cellulose. The powder-filled capsules will be kept in a secure, limited access storage area out of direct sunlight or artificial light and stored between 55 and 75 degrees Fahrenheit in the UMRP.

5.8 Distribution

5.8.1 Stratified Randomization

Study participants will be randomly assigned to receive either Aquamin® or identical looking capsule of placebo (maltodextrin) in a 1:1 ratio. This paired randomization will provide equal group size at the halfway point of the study. The study biostatistician will provide the UMRP with randomization codes. The biostatistician will also consider randomization in a way to ensure balance within each gender.

Upon enrollment, a participant will be assigned a subject ID by the study personnel. The personnel will subsequently contact the UMRP with the relevant participant information. The UMRP will then dispense the treatment according to the randomization code provided to them. Only the UMRP staff will be aware of the subject treatment assignment. After first 90 days, subjects on placebo will be crossed over to Aquamin® for the last 90 days.

Subjects will be unblinded for the safety assessment and interim analysis. Thus, the first 90-day period constitutes a double-blind study and the second 90-day period is an open-label study.

5.8.2 Blinding and Unblinding

Participants, researchers, and all study personnel will be blinded to study assignment during the initial 90 days period. All capsules will look identical. Only the UMRP staff will be aware of what treatment study participants are receiving. A study participant can be unblinded if there is a clear medical need to know what treatment assignment the participant received, e.g. receiving a medical procedure where the study agent may interact. Only the study PI can authorize the unblinding of a participant. Anyone requesting unblinding needs to contact the PI who will determine if unblinding is necessary. The PI will then contact UMRP and obtain the study assignment and give this information to the appropriate party such as the participant's physician. The study will be unblinded after the first 90 days of participation for the purpose of crossover of the subjects on the placebo to Aquamin® treatment. This unblinding will also allow for the conduct of the safety assessment and interim analysis to generate interim data results. This interim data collection will not affect future recruitments.

5.9 Dose Modification

No dose reduction will occur due to toxicity. If a study subject should experience any related serious adverse events or symptoms that are intolerable, he/she will be removed from the study.

5.10 Adherence

Adherence will be assessed by capsule count and calendar review as well as monthly phone calls. Each subject will be given a sufficient number of capsules for each 90-day period and instructed to return all unused capsules at the 90-day visit and 180-day visit. The actual quantity of unused capsules will be compared to the anticipated amount of unused capsules and to the subject's calendar. The calendars will be provided to help subjects track their current doses and to record the date and time the doses were taken. Electronic means (mobile phone calendars or other applications, computer software, etc) for recording individuals' dates and times of doses taken will be allowed if it helps with subject adherence.

Adherence will be assessed at each visit. Subjects will be considered adherent if they take 75% or more of their scheduled dose **AND** they take 75% of their dose on 75% of the days. A table is provided in Appendix 8 to calculate adherence. If subjects did not keep their diary up-to-date during their dosing, adherence will be assessed by pill count and querying the subject. Discrepancies or concerns about adherence with dosing may lead to the subject being replaced on study.

5.11 Ordering Capsules for a Participant

When study personnel enroll a participant, a study team member will provide the prescription for the study agent to UMRP. This prescription will include the name of the study, the study protocol number, the study participant's name and study number, the ordering study team member, the address and fax number of the PI, and the drug and administration instructions. Study capsules will be provided in two installments, each for 90 days.

5.12 Capsule accountability

The UMRP will use an Accountability Form for the active ingredient and placebo capsules. Information on drug receipt, return and disposition, and on capsules preparation and destruction will be logged on the form. During the course of the study, the randomization number and initials of the participant to whom drug is dispensed, the date(s) and quantity of drug dispensed to the participant, and the date(s) and quantity of drug return by the participant will be recorded on the log. Participants should return unused capsules to the study team at the end of days 90 and 180; these will be returned to the UMRP for counting and disposition. These Accountability Forms must be readily available for inspection at any time.

5.13 Drug Disposal

Any unused capsules or empty bottles will be collected from study participants during their last point of contact. These capsules will be brought to the UMRP. UMRP will record the returned drug on the Accountability Forms and dispose of the unused capsules on site.

6 CRITERIA FOR EVALUATION AND ENDPOINT DEFINITION

6.1 Evaluation Endpoints

6.1.1 *Primary Objectives*

To evaluate whether Aquamin® taken daily, standardized to 800 mg of calcium per day for 90 and 180 days, will improve clinical and/or laboratory findings of ulcerative colitis in subjects with ulcerative colitis in remission.
Clinical and / or laboratory features to be evaluated will include i) IBDQ scoring based on subjects' responses to the questionnaire, ii) gastroenterologist's clinical assessment and endoscopic findings by employing Ulcerative Colitis Disease Activity Index (UCDAI) – Mayo Score with stool frequency, rectal bleeding and endoscopic sub scoring. iii) histological assessment of the colon tissue by a board-certified pathologist. iv) fecal calprotectin and serum CRP levels. These will be assessed at baseline, at the mid-point visit (Day 90 ± 5 days) and final visit (day 180 ± 5 days).

To evaluate whether Aquamin® taken daily, standardized to 800 mg of calcium per day for 90 and 180 days, will increase markers of differentiation, improve defined mucosal markers of barrier function and decrease mucosal inflammation in subjects with ulcerative colitis in remission.
Markers of differentiation and barrier function / tissue cohesion will be evaluated by quantitative immunohistology and proteomic analysis. Colonic tissue IL-8 levels will be assessed by ELISA.

These will be assessed at baseline, at the mid-point visit (Day 90 ± 5 days) and final visit (day 180 ± 5 days).

6.1.2 *Secondary Objectives*

To determine whether Aquamin® taken daily, standardized to 800 mg of calcium per day for 90 and 180 days, will alter microbial and metabolic profiles versus placebo.
Fecal specimens will be obtained at baseline, at the mid-point visit (Day 90 ± 5 days) and final visit (day 180 ± 5 days). These will be evaluated by microbial and metabolomic analysis. A salivary sample will be collected at the same time-points and evaluated for oral microbial flora.

To determine whether Aquamin® taken daily, standardized to 800 mg of calcium per day for 90 and 180 days, will alter levels of liver enzymes in serum versus placebo.
An SST tube of blood will be drawn at baseline, at the mid-point visit (Day 90 ± 5 days) and final visit (day 180 ± 5 days). Liver enzymes (ALT, AST and ALKP) as part of Comprehensive Metabolic Panel (COMP) will be assessed in serum.

To determine whether Aquamin® taken daily, standardized to 800 mg of calcium per day for 90 and 180 days, will alter levels of serum bone turn over markers versus placebo.
Ten cc of blood (red top) will be drawn at baseline, at the mid-point visit (Day 90 ± 5 days) and final visit (day 180 ± 5 days). Serum biomarkers of bone turn-over (osteocalcin, P1NP, serum collagen type 1 cross-linked C-telopeptide [CTX], bone-specific alkaline phosphatase) will be assessed. Bone mineral density will also be assessed using a DEXA scan to correlate these markers on baseline and final visits.

6.2 Protocol Endpoint

The study will end once colon biopsies, fecal specimen and blood / saliva have been collected from all the visits – i.e., the baseline visit (day 0), halfway visit (day 90) and the final visit (day 180) - from 40 participants who meet evaluable criteria and the final participant is declared to be off-study. We estimate that we will need to consent and enroll about 48 subjects to reach 40 evaluable subjects due to dropouts, non-adherence and other reasons. Genetic testing will not be performed on any of these tissue samples unless a subject gives us permission to do so. Data analysis will continue after the end of the study and we will continue to maintain regulatory approval until data analysis is complete.

6.2.1 *Additional studies*

An additional sub-study will be conducted. Subjects will be asked if they wish to take part in the sub-study. If they agree to take part in this sub-study, they will be asked to:

- Allow us to save or store coded colonic tissue, stool and serum samples that will be taken at visits 2 and / or 3 and / or 4 either as frozen samples or banked after fixing in the formalin and embedding in paraffin (in case of colon tissue) for future use. If they do not agree to participate in the sub-study, no tissue will be saved for unspecified future use.

- As part of the sub-study, within the main study, it is possible that the coded colonic tissue, serum and stool samples will be saved at a University of Michigan approved storage facility. The storage facility may process the samples and then send portions of these samples to different laboratories (potential collaborators) for analysis. One type of laboratory might analyze DNA by a method called sequencing or lasing threshold. Other laboratories could study these samples by different methods. The remaining tissue might be stored for an unlimited period of time for use in future research focused on preventing, treating, or detecting ulcerative colitis, colon cancer, or perhaps used in other research projects.
- Information obtained from the analyses performed on these coded samples and coded medical information will be entered into Internet-accessible databases along with information acquired by other research participants in this project.

Deidentified information from the analyses, which cannot be traced to any individual subject, may be available to anyone with access to a public Internet database. Information obtained from the analysis of any tissues/specimens and corresponding medical/clinical information may be placed in a controlled access database. This information will only be available to researchers who have received approval from an NIH data-access committee.

7 CLINICAL EVALUATIONS/PROCEDURES

7.1 Schedule of Events

Evaluation/Procedures	Pre-Study-visit 1	Baseline-visit 2 Day 0	[A] Weekly/ Bi-weekly, Monthly	Halfway-visit 3 Day 90±5	Final-visit 4 Day 180±5
Informed Consent	X				
Medical History / Physical Exam (Phenotyping) [B]	X				X
Morphometric measurements [B]		X		X	X
Urine Pregnancy Test [C]	X	X		X	X
Concomitant Medication/ Supplement Use/normal diet	X	X	X	X	X
Monitoring / Assessment by SIBDQ [D]			X		
Clinical assessment / IBDQ / UCDAI [E]		X		X	X
Randomization		X			
Colon Biopsies and endoscopy [F]		X		X	X
Dispense Study Medication		X		X	
Adherence/Toxicity Evaluation/ Adverse Events		X	X	X	X
Dietary History Questionnaire [G]	X	X	X	X	
Off-Study Form					X
Unblinding: Safety Assessment and crossover				X	
Pill Count				X	X
Blood draw		X		X	X
Stool sample [F]		X		X	X
Saliva sample		X		X	X
Fecal Calprotectin		X		X	X

[A] Monitoring (for assessment of toxicity, adherence and a reminder to complete the diet questionnaire) will be completed by phone call or email reminder on the following days; Days 7, 14, 21 (+/- 2 days), and Days 30, 45, 60, 120 and 150. (+/- 5 days).

[B] Physical Exam (Phenotyping) must be done within 60 days of the baseline biopsies. At visit 3, physical exam will consist of body weight measurement. Height will be measured only on Visit 2.
[C] The pregnancy test which must be valid within 14 days of the first dose of study medication. If the time between visit 1 and visit 2 is greater than 14 days, the pregnancy test must be repeated.

[D] Disease assessment (stool frequency, potential rectal bleeding, and abdominal pain) by using SIBDQ via telephone (or email) at Days 7, 14, 21 (+/- 2 days), and Days 30, 45, 60, 120, and 150 (+/- 5 days).

[E] Assessed and reviewed by an MD (Dr. Kim Turgeon).
[F] Performed and collected by an MD during the unprepped flexible sigmoidoscopy.
[G] Given at visit 2 and collected at the visit 3 with reminders at phone monitoring.

7.2 Visit #1-Screening Visit

Interested, healthy subjects with stable ulcerative colitis who responded to recruitment advertising and who qualify based on a phone/email screening questionnaire will be scheduled for a screening visit. At the screening visit, a member of the study team will describe the study and review the consent document. If the subject is eligible and interested in participating, they will sign the informed consent form. The following tasks will be performed.

1. Eligibility assessed

2. Informed consent signed

3. Food questionnaire with questions asked about the concomitant medication/supplement use/medical history/past year food intake administered

4. A physical examination (Phenotyping) done by an MCRU LPN or MA

5. Vital signs assessed (seated blood pressure, pulse, respiratory rate, and temperature)

6. Wash out period established for steroids, antibiotics, NSAIDs and supplement use, if applicable

7. Visit 2 (Day 0 visit) scheduled

8. Urine Pregnancy test performed (if applicable)

7.2.1 Concomitant Medication and Supplements

Concomitant dietary supplements (e.g., herbal, prescribed or over-the-counter medications) are allowed except for those medications that could obscure our ability to detect the effects of Aquamin®. Potential participants will be asked about specific supplement use that could obscure the ability to detect the effects of Aquamin®. These include calcium, Vitamin D or combinations of the two. Subjects will also be asked about their use of antacids containing calcium or foods with added calcium. If a participant is taking one of these supplements or consuming extra, added calcium, he/she could still be eligible to participate in the study if he/she is willing to stop taking the supplements for at least thirty days before study entry and for the study duration. Antibiotics (rocephin, keflex, omnicef) and corticosteroids are also contraindicated at the start of the study but exempt at the time of flare-up during the study. Subjects will not be allowed to participate in any other interventional trial using a new therapeutic during the entire course of the study.

7.3 Visit #2-Day 0 (Baseline Visit)

After reviewing the subject's data, eligible subjects will be contacted to schedule an enrollment visit. On Visit#2, the following tasks will be conducted.

1. Data collection including review of eligibility performed.

2. Concomitant medication/supplement updates collected.

3. Clinical assessment conducted by an MD using UCDAI and IBDQ collected.

4. Vital signs assessed (seated blood pressure, pulse, respiratory rate, and temperature)

5. Morphometric measurements (height and weight, waist/hip measurements) determined.

6. Blood sample taken (Appendix 6)

7. Salivary sample collected (Appendix 7)

8. Comprehensive Metabolic Panel (COMP) (SST tube) and CRP performed

9. DEXA scan performed

10. Serum specimen (10 ml Red top tube) for later assessment of Bone biomarkers saved

11. Urine Pregnancy Test conducted, if applicable and more than 14 days since visit 1

12. Baseline Flexible Sigmoidoscopy performed (Appendix 4) with colon and fecal specimen collection

13. Study agent dispensed (3 months' supply)

14. Calendar to record compliance is given to the subject to take home

15. Payment of $50.00 for completion of Visit 2 arranged

7.3.1 Collection of stool samples and colon tissue

Participants will be requested to evacuate their bowel as close to the biopsy procedure time as possible. Other than this, no bowel preparation procedure will be used. A flexible sigmoidoscopy will be performed in the MCRU by a gastroenterologist who is a trained endoscopist. The biopsies taken at the procedure will then be frozen in liquid nitrogen or fixed in formalin after being placed in designated, labeled containers. Each colonic biopsy obtained during the sigmoidoscopy will weigh about 5 mg and contain approximately 500 µg protein (65). Appendix 4 provides details of the flexible sigmoidoscopy procedure. Stool specimens will be obtained during the same procedure (Appendix 4).

Seven sterile stool samples will be collected (may need multiple passes to collect approximately 50 mg of stool per sample) from the sigmoid colon using sterile biopsy forceps at the time of flexible sigmoidoscopy. Stool collection is followed by a collection of colonic mucosal biopsies (twelve at the baseline visit and ten at the mid-point and final visits) and which will be obtained by using new sterile biopsy forceps so that enough tissue is available for assessment of each biomarker, metabolomics, microbial and proteomic analysis (Appendix 5). These endoscopic biopsies will be obtained from the sigmoid colon circumferentially at least 15 cm above the anus using biopsy forceps. Dr. Kim Turgeon, a physician with years of experience doing flexible sigmoidoscopies will be doing the procedure and collecting stool and colonic tissue biopsies.

7.3.2 Toxicity assessment

Toxicity will be assessed via telephone call or email, once per month during the course of the study and at the baseline visit (Visit #2), at the mid-point visit (Day 90 ± 5 days) and at the final visit (day 180 ± 5 days).

7.3.3 Compliance and Blinding Assessment

Compliance will be assessed by pill count and calendar. Participants will be classified as adherent if the adherence monitoring suggests that 75% or more of the doses were taken as prescribed. This count will be conducted during Visit #4. Subjects will be asked to scan, photograph or fax copies of their calendar each month for an additional check of adherence.

7.4 Safety monitoring

This will comprise an assessment of toxicity, adherence and a reminder to complete the diet health questionnaire by telephone or email or at a visit. The frequency of the monitoring will be weekly for the first month, every two weeks for the second month and monthly onwards; Days 7, 14, 21 (+/- 2 days), and Days 30, 45, 60, 120 and 150. (+/- 5 days). To monitor disease activity and to gather general AE information, a questionnaire- SIBDQ will be used at Days 7, 14, and 21 (+/- 2 days), and Days 30, 45, 60, 120, and 150 (+/- 5 days). This will be in addition to the collection of IBDQ and clinical assessment performed at the Baseline, Halfway and Final Visits using Ulcerative Colitis Disease Activity Index (UCDAI) based on Mayo Scoring. These clinical assessments will be made by Dr. Kim Turgeon.

7.5 Visit #3- Day 90 (Halfway Visit)

After 90 days, on Visit #3, the following tasks will be conducted.

1. Data collection including review of eligibility performed

2. Concomitant medication/supplement updates collected

3. Clinical assessment conducted by an MD using UCDAI and IBDQ collected.

4. Physical examination - phenotyping (body weight measurement, waist/hip measurements) performed and conducted by either by study team, by MD or MCRU LPN or MA.

5. Vital signs assessed (seated blood pressure, pulse, respiratory rate, and temperature)

6. Blood specimen obtained (Appendix 6)

7. Salivary sample collected (Appendix 7)

8. Blood sample sent for Comprehensive Metabolic Panel (COMP) (SST tube) and CRP

9. Serum specimen saved for later research assays (10 ml red top tube) including bone biomarker assessment

10. Flexible Sigmoidoscopy performed (Appendix 4)

11. Calendars, pill bottles and Dietary History Questionnaire collected

12. Study agent-dispensed (3 months' supply with a new calendar)

13. Payment of $150.00 for completion of Visit 3 arranged.

14. Urine Pregnancy Test performed, if applicable

15. Participants asked about any medication use during the study period.

16. Placebo group crossed over to Aquamin®.

17. Participants asked to indicate what treatment they believed they received: "don't know", "Aquamin®", or "placebo".

7.6 Visit #4-Day 180 (Final Visit)

After 180 days, on Visit #4, the following tasks will be conducted.

1. Data collection including review of eligibility performed

2. Concomitant medication/supplement updates collected

3. Clinical assessment conducted by an MD using UCDAI and IBDQ collected

4. Physical examination- phenotyping (body weight measurement, waist/hip measurements) performed by study team, by MD or MCRU LPN or MA.

5. Vital signs assessed (seated blood pressure, pulse, respiratory rate, and temperature)

6. Blood specimen obtained (Appendix 6)

7. Salivary sample collected (Appendix 7)

8. Blood sample sent for Comprehensive Metabolic Panel (COMP) (SST tube) and CRP

9. DEXA scan performed

10. Serum specimen saved for later research assays (10 ml red top tube) including bone biomarkers assessment

11. Final flexible sigmoidoscopy performed (Appendix 4)

12. Calendars and pill bottles collected

13. Payment of $200.00 for completion of Visit #4 arranged.

14. Participants asked about any medication use during the study period.

7.7 Documentation of Medication

All medications (prescription and over-the-counter) and dietary supplements taken by the participant during the study will be documented by the subject on their calendar and/or during the monthly assessment. Information will include:

- Start and stop dates of drugs, herbs, or supplements
- Dose
- Purpose for taking the medication, herbs, or supplements

7.8 Medication during Trial

As this is a 180-day study, it is unrealistic to expect all individuals to comply with a zero tolerance approach to concomitant medication. However, it is expected that all individuals will abstain from adding new supplements (whether over-the-counter, natural or dietary). Should the need for concomitant medication arise, then the participant will be asked to notify the research personnel. The dose, date, and reason for use will be recorded on the Case Report Form (CRF). Should a need for prescribed medication arise, the cause may be considered as an adverse event (whether or not classified as drug-related) and a clinical decision as to withdrawal from the study will be discussed.

7.9 Scheduling issues, missed doses, extenuating circumstances

The days presented above are ideal for the protocol. However, when dealing with volunteers, unexpected things happen, especially to schedules. If there are extenuating circumstances preventing the visit from occurring as scheduled, or too many doses are missed due to things like vomiting/diarrhea-based illness, or other unexpected, but manageable problems occur, we may provide additional agent to keep the levels steady while we reschedule the visits. These variations will be noted in the study file and on appropriate case report forms. UMRP will be asked to dispense additional agent capsules as needed to keep subjects on agent while scheduling visits. Subjects who demonstrate non-adherence (as defined above in section 5.10) may be taken off study. Our biostatistician, study team and/or DSMC will determine whether a subject should continue on study or be taken off and replaced.

7.10 Off Study

Unless there are any ongoing SAE's, subjects are officially "Off Study" after the final contact 2 weeks after taking the final dose. During this Post-agent Day# 14 (±5 days), subjects are contacted to be assessed for toxicity and informed that their part in the trial is complete.

8 ADVERSE EVENTS

An adverse event (AE) is any condition, which appears or worsens after the subject is enrolled in an investigational study. Any adverse events will be noted on the Adverse Event Case Report Form (CRF), whether or not related to taking a study agent/placebo. Adverse Events will be reported to the Data and Safety Monitoring Committee and to the IRBMed per IRBMed reporting requirements.

8.1 Adverse Event Reporting

- Start and stop dates
- Severity (grade)
- AE term (verbatim and CTCAE category/term)
- Whether or not AE was reported as an SAE
- Relationship to study agents (attribution) or placebo
- AE outcome
- Whether or not the subject withdrew due to the AE

8.1.1 Severity and grading

Adverse events will be graded by a numerical score according to NCI Common Terminology Criteria for Adverse Events (CTCAE), version 4.0.

NOTE: Situations may arise where the Common Toxicity Criteria, Version 4.0 do not represent certain agent-specific effects, severity of these effects, and attribution of the effect. On these occasions the CTCAE criteria may be expanded or modified to include these situations. Adverse Events not included in the CTCAE will be scored according to their impact on the subject's ability to perform daily activities as follows:

Grade	Severity	Description
1	Mild	Barely noticeable, does not influence functioning<br>Causing no limitations of usual activities
2	Moderate	Makes subject uncomfortable, influences functioning<br>Causing some limitations of usual activities
3	Severe	Severe discomfort, treatment needed<br>Severe and undesirable, causing inability to carry out usual activities
4	Life threatening	Immediate risk of death<br>Life threatening or disabling
5	Fatal	Causes death of the subject

8.1.2 Follow-up

All AE's, including laboratory abnormalities that, in the opinion of the Investigator, are clinically significant, will be followed up according to Good Clinical Practice, and documented as such.

8.1.3 Serious Adverse Events (SAE)

DEFINITION: ICH Guideline E2A and Fed. Reg. 62, Oct. 7, 1997 define serious adverse events as those events, occurring at any dose, which meet any of the following criteria:

- Results in death
- Is life threatening (Note: the term life-threatening refers to an event in which the subject was at risk of death at the time of the event; it does not refer to an event which hypothetically might have caused death if it were more severe).
- Requires inpatient hospitalization or prolongation of existing hospitalization
- Results in persistent or significant disability/incapacity
- Is a congenital abnormality/birth defect
- Events that may not meet these criteria, but which the investigator finds very unusual and/or potentially serious, will also be reported in the same manner.

8.1.4 Withdrawal of Subject from Trial after SAE

If a SAE is deemed possibly, probably or definitely related to the agent assigned, the subject will stop taking the agent and go off-study. If an SAE is unrelated to the study agent, advice will be sought from the PI, physicians of the study team and/or Data Safety Monitoring Committee (DSMC) regarding the continuation of dosing for that subject.

8.2 Frequency of Monitoring

Adverse events will be monitored from the Screening Visit to the Final Visit at Day 180 (± 5 days) and within 24 hours of the final dose. Subjects will also be contacted at 14 days' post completion of the study. Participants will be contacted via telephone or email, weekly in the first month, bi-weekly for the second month and once per month while on study onwards. Participant will also be asked to contact the researchers by telephone or email if they experience any adverse events. Dr. Kim Turgeon will be the clinical monitor and will be responsible for safety monitoring during the study. Dr. Turgeon's phone number (a twenty-four-hour number) will be listed on the consent form for emergency reporting.

8.3 Plan for Reporting Adverse or Other Reportable Events or Information

8.3.1 Reporting to the IRBMED

Only adverse events considered by the PI to be at least possibly related to the study procedures or study agents (Aquamin® or placebo) will be reported. "Related" means events that are caused by the research itself, not the disease or population under study. Reports will be sent to the IRB as follows:

1. Adverse event is defined as "serious" - report to the IRB within seven days

2. Non-serious adverse events grade 2 or higher - report to IRB prior to Scheduled Continuing Review (SCR).

3. Any unanticipated problems that are related to the study and indicate risks to subjects, (serious problems within seven days, non-serious problems within 14 days) according to the current IRB reporting recommendations.

4. Privacy violation or breach of confidentiality (report to IRB within seven days, and within 24 hours to the UMHS Privacy Office)

5. Protocol deviations - report with SCR

8.3.2 Reporting to the FDA

The Sponsor will collaborate with the Michigan Institute for Clinical and Health Research (MICHR) IND/IDE Investigator Assistance Program (MIAP) office for the reporting of any and all IND safety reports to the FDA as per the requirements outlined in 21 CFR 312.32.

9 DOSE/TREATMENT MODIFICATION AND STUDY STOPPING CRITERIA

9.1 Dose/Treatment Modification Criteria

This criterion will be dictated by study physician (Dr. Kim Turgeon) or by the DSMC during the course of the study whenever there is a safety or toxicity issue. Once subjects start their study drugs after Visit #2 (on day 0), they will continue their ingestion until completion of Visit 3 (on Day 90±5). After Visit #3 (on Day 90±5), a safety assessment will be performed (and reviewed by the DSMC) to decide if a subject should continue to be on the study until day 180±5 days. This safety assessment will comprise review of any AE/SAE data, evaluation of liver enzyme data and assessment by our medical director (Dr. Turgeon). The DSMC will also assess any toxicity issues and evaluate any AE/SAE during their regular monthly meetings and make a decision to stop or modify the study drug or further participation of the subject in the study accordingly as based on the following rules.

- In case of a flare-up, subjects will continue their study drugs unless DSMC or Dr. Turgeon decides to temporarily stop participation. Study team will record flare-up events and all the medications taken during those periods. Subjects will resume study drugs as soon as Dr. Turgeon or DSMC think it is safe to do so.

- After the completion of Visit #3 (at day 90±5), interim safety parameters including review of AE/SAE, clinical assessment (clinical presentation and endoscopic findings evaluated by Dr. Turgeon), and serum liver function test (AST, ALT, and ALKP) results will be assessed by Dr. Turgeon and DSMC. Based on this assessment, decision will be made for a subject to continue to take the study drugs or cease. Subjects will continue their study drugs as per protocol on Day 91±5 until the DSMC makes a decision in the next two weeks.

- Serum liver enzymes (AST, ALT, and ALKP) will be assessed at baseline, and at Day 90±5. Liver enzymes will be compared to the baseline values and the normal reference range values to assess any insult to the liver. The subjects will stop the study drug if there is a huge spike in the liver enzymes (5 fold higher than the baseline value at Day 0 [visit 2] or upper value in the normal reference range - whichever is higher) in the liver function test at the Day-90 interim safety assessment. If an individual's participation is suspended due to liver enzyme values being too high, subjects could resume their participation (and start the study drug) as soon as their liver function test levels come back to the safe range and after an approval by the physician.

9.2 Study Stopping Criteria

An individual has to stop the participation if his/her health is severely affected by the study drug or any other serious health related issues during the study. The study should be stopped in case when more than 50% subjects;

- Have a SAE with the test drug (Aquamin®) or
- Have liver enzyme (AST, ALT, ALKP) levels 5 times higher than their baseline or upper normal reference range values (consistently high even after stopping the study drug) in the Aquamin® group.

The trial will be completed when the final tissue samples have been obtained from the last subject. Subjects will be followed for 14 days after the last dose of the investigational agent. A telephone call or email will be the means for contact.

9.3 Premature Removal of a Participant

9.3.1 Reasons for Premature Withdrawal from the Study

9.3.1.1 Participant withdrawal

A participant may withdraw voluntarily from the study at any time without necessarily giving the investigator reason. In such a case, efforts will be made to schedule a visit to return study materials.

9.3.1.2 Non-compliance with dosing

Should there be <75% of expected doses on self-report, calendar review or pill count (Appendix 8), then investigators and study personnel will address this issue with the participant. If after discussion with participant this cannot be corrected or if this non-compliance occurs more than three times over the 180-day trial period, the participant shall be withdrawn from study.

9.3.1.3 Lost to follow-up

Diligent attempts must be made by telephone, email and letter to determine the circumstances for loss to follow-up, since such loss may be related to the study drug.

9.3.1.4 Unacceptable adverse effects

If a participant develops adverse effects, investigators will follow guidance in Section 8.0 regarding possible termination of study. They will be medically treated as clinically necessary and will continue to be followed up until such event resolves.

9.3.1.5 PI, Investigator Discretion

The Principal Investigator can decide to terminate a participant's participation in the study at any time. This decision could be based on factors such as unacceptable adverse events or for other safety concerns.

9.3.2 Recommendation in Case of Premature Withdrawal from the Study

All participants removed prematurely will be replaced with a new participant unless instructed otherwise by the Study PI or DSMC.

10 DATA MANAGEMENT

10.1 Case Report Form Set

The CRFs, a set of forms for each participant, provides a record of data generated according to protocol. These forms are to be completed on an ongoing basis during the study. The research chart is the source of verification of data. During the study, CRFs will be monitored for completeness, accuracy, legibility and attention to detail. The CRFs will be retained for review.

10.2 Data Entry, Data Management and Quality Control

Hard copies of the data are kept in folders in the study team's office. The coded list of participants on the study is also maintained by the Study Coordinator. Confidentiality of the participants will be maintained and information kept on each participant will be made available only to Study Coordinators and identified investigators. RedCap is an electronic data capture (EDC) system that is secure, HIPAA compliant, and web-based that will also be used.

11 STATISTICAL CONSIDERATIONS

This is a randomized, double-blinded, placebo-controlled pilot study to determine if a daily dose of Aquamin® compared to placebo alone alters clinical and laboratory findings related to ulcerative colitis, as a way to assess efficacy. In parallel, it is a randomized, double-blinded, placebo-controlled pilot study to determine if Aquamin® alters biomarkers of differentiation and barrier function / tissue integrity in the colonic mucosa of subjects with ulcerative colitis in remission. Immunohistochemistry and proteomics will be used for these endpoints. In addition, metabolomics and microbial analyses will also be done. An additional goal is to determine if the same treatment alters serum liver enzyme levels or biomarkers of inflammation in serum. ELISAs will be used for assessment of serum biomarkers.

11.1 Study Design

Forty evaluable participants will be randomized to receive active treatment or placebo. Participants will take the study drug twice daily for 180 days (± 5 days) starting the day the participant receives their baseline flexible sigmoidoscopy (unprepped) until within 24 hours of

their last dose and their Day 180 flexible sigmoidoscopy. Colonic biopsies (twelve at the baseline visit and ten at the mid-point and final visits) and seven fecal specimens will be taken during the flexible sigmoidoscopy performed at the start, halfway, and conclusion of the study. Subjects on the placebo will be crossed over to Aquamin® after the halfway visit (at Day 90). The Aquamin® group will ingest Aquamin for the entire 180 days.

11.2 Analysis Plan

11.2.1 Primary Aims

To evaluate whether Aquamin® taken daily, standardized to 800 mg of calcium per day for 90 and 180 days, will improve clinical and/or laboratory findings of ulcerative colitis in subjects with ulcerative colitis in remission.

The primary outcome of change in clinical features will be evaluated by i) IBDQ scoring based on subjects' responses to the questionnaire, ii) clinical presentation and gastroenterologist's endoscopic findings based on UCDAI / Mayo Scoring, iii) histological assessment of the colon tissue by a board-certified pathologist and iv), laboratory (fecal calprotectin and serum CRP levels) findings. We expect improved IBDQ and UCDAI scores and endoscopic findings with histological representation of better crypt structure with decreased inflammatory cells in the *lamina propria*. Levels of fecal Calprotectin and serum CRP should be decreased or remain stable with treatment. Values for all features (pre- post-values) will be measured and compared using appropriate statistical methods performed by the study biostatistician and the program GraphPad Prism, version 8.0 may also be used.

To evaluate whether Aquamin® taken daily, standardized to 800 mg of calcium per day for 180 days will alters markers of proliferation, differentiation and barrier structure in the colonic mucosa.

Markers of proliferation (Ki67 or PCNA), differentiation (CK20) and barrier structure (cadherin-17, claudin-23 and desmoglein-2) versus placebo will be evaluated using quantitative immunohistology and proteomics exactly as done in our recent studies (56,57), at Day-90 and Day-180 samples.

For each value of interest, we will have a pre- post-comparison for all subjects at three time-points. For values that are normally distributed, we will have group means and standard deviations. We will assess differences by ANOVA followed by paired group comparisons. Data that are not normally distributed will be evaluated by Mann Whitney U. A multiple regression analysis will be carried out controlling for age and gender as covariates. Appropriate model diagnostics will be performed followed by any necessary corrective actions. All appropriate statistical analyses will be performed by the study biostatistician and the program GraphPad Prism, version 8.0 may also be used by the team.

11.2.2 Secondary Aims

To evaluate whether Aquamin® taken daily, standardized to 800 mg of calcium per day for 180 days, will alter microbial and metabolic profiles versus placebo.

The analysis plan for the secondary aims is identical to that for the primary aim, *albeit* with different outcome measurements. Microbial and metabolomics analysis will be performed on colon and stool specimens. Salivary samples will be evaluated for oral microbial flora. Samples collected at Day 180 will be compared with baseline and Day 90 samples. Microbial sequencing data will be evaluated initially in the core laboratory that carriers out the sequencing (Host Microbiome Initiative Lab at the University of Michigan).

When the core provides the sequencing data to us, these data will be evaluated along with metabolomics data and serum chemistry. Specifically, for each value of interest, we will have a pre- post-comparison for all subjects at three time-points. For values that are normally distributed, we will have group means and standard deviations. We will assess differences by ANOVA followed by paired group comparisons. Data that are not normally distributed will be evaluated by Mann Whitney U. A multiple regression analysis will be carried out controlling for age and gender as covariates. Appropriate model diagnostics will be performed followed by any necessary corrective actions. All appropriate statistical analyses will be performed by the study biostatistician and the program GraphPad Prism, version 8.0 may also be used.

To evaluate whether Aquamin® taken daily, standardized to 800 mg of calcium per day for 90 and 180 days, will alter levels of liver enzymes in serum versus placebo.

Liver enzymes (ALT, AST and ALKP) as part of Comprehensive Metabolic Panel (COMP) will be assessed in serum at baseline, day 90 and day 180 as part of the safety studies. We expect minimal changes in the liver enzymes after 90 and 180 days of intervention in response to Aquamin®. The levels at day 90 and day 180 will be compared to the baseline levels, pre-post comparison within each subject and group averages (pre-post) will also be evaluated and with placebo at day 90.

To evaluate whether Aquamin® taken daily, standardized to 800 mg of calcium per day for 90 and 180 days, will alter levels of serum bone turn over markers versus placebo.

Serum bone markers will be assessed using commercially available ELISA kits and we expect improvement in these markers with Aquamin® as compared to the placebo group at day 90. Additional pre-post comparison will also be performed in all subjects at day 90 and day 180. Serum bone markers will also be correlated with bone mineral status using BMD at the baseline and final visits.

11.2.3 Sample Size Justification

The study will randomly allocate 20 patients to each of two groups: Aquamin® (for 180 days), or placebo (for first 90 days/Aquamin® for last 90 days), for a total of 40 evaluable subjects. Gender stratification will be carried out to ensure balance within males and females separately. Anticipating a 15%-20% drop out evenly across arms, we plan to recruit 24 subjects per arm making a total recruitment target of 48 subjects. This is planned as a feasibility, tolerability, and research markers study. Consequently, it may not detect statistically-significant differences across study arms. However, we anticipate that the study will demonstrate trends that will be useful in generating research hypothesis for a future fully powered large-scale study.

12 ETHICAL AND REGULATORY CONSIDERATIONS

12.1 Institutional Review Board (IRB) Approval

Prior to initiating the study and receiving the study agents, the PI must obtain written approval to conduct the study from The University of Michigan Medical School Institutional Review Board (IRBMED). Should changes to study protocol become necessary, protocol amendments will be submitted by the PI to the IRBMED prior to implementation.

12.2 Informed Consent

All potential candidates for the study will be given a copy of the study's informed consent to read. Research personnel will explain all aspects of the study in lay-language and answer all of the candidate's questions regarding the study. If the candidate decides to participate in the study, he/she will be asked to sign the informed consent document. The study agent will not be released to a participant and no procedures performed on a subject who has not signed the informed consent document. Participants who refuse to participate or who withdraw from the study will be treated without prejudice.

The informed consent document must be reviewed and approved by the IRB prior to study initiation. Any subsequent changes to the informed consent must be approved by the IRB prior to activation.

12.3 Data and Safety Monitoring Plan

The study team will meet approximately monthly to review recruitment, enrollment, AEs and protocol deviations. In addition, the Data Safety and Monitoring Committee (DSMC) will meet monthly to review the study. The members of this committee will be an experienced clinical investigator, the principal investigator, the project manager and a biostatistician. During these meetings, members will discuss matters related to safety of protocol participants (AE reporting), validity and integrity of the data, enrollment rate relative to expectation, characteristics of participants, retention of participants, adherence to protocol (potential or real protocol violations) and data completeness. Members will also discuss the safety assessment parameters at the completion of day 90±5 visit for each subject within 2 weeks. Data and safety monitoring reports of these regular meetings will be kept on file.

12.4 Regulatory Agency Monitoring

The FDA may monitor/audit various aspects of the study. These monitors will be given access to facilities, databases, supplies and records to review and verify data pertinent to the study. The study will be audited internally as detailed in Appendix 9.

12.5 Record Retention

Clinical records for all participants studied, including CRFs, history and physical findings, laboratory data, and results of consultations will be maintained by the Investigator in a secure storage facility and stored until destroyed.

12.6 Data Safety and Monitoring

12.6.1 Authority and Duties

The Study Data Safety and Monitoring Committee (DSMC) reviews, makes recommendations, and acts on the following:

- Progress towards completion of the study—recruitment and retention of study subjects, and enrollment rate relative to expectation
- Evaluation of interim new information
- Evaluation of toxicity events and subject safety, including adverse events
- Evaluation of interim safety parameters after Visit #3 (Day 90±5).
- Timeliness of data
- Quality of data
- Ethical conduct of research
- Adherence to the protocol

Data and safety monitoring reports of these regular meetings are kept on file with the DSMC facilitator in the HEMONC group.

The DSMC is empowered with the authority to recommend a study be suspended or terminated based upon concerns in any of the above areas of review. Monitoring also considers factors external to the study, such as scientific or therapeutic developments that may have an impact on the safety of the subjects or the ethics of the study. Recommendations that emanate from monitoring activities are reviewed by the principal investigator and addressed.

12.6.2 Composition

Membership will include faculty members from Gastroenterology and Family Medicine (cancer prevention/biomarker development related projects). The members of this committee will include experienced clinical investigators. At least three faculty members must be present to have quorum. (More information regarding Data safety and Monitoring plan is present in Appendix 10).

12.6.3 Meeting Frequency

The DSMC meets monthly by means of regularly scheduled meetings. Prior to each meeting, the Clinical Research Associate (CRA) distributes a standard summary report detailing accrual, deviations, eResearch amendments, quality control audit information, any ethical concerns, patient-subject complaints, and adverse events or serious adverse events. Members may meet out

of regular schedule to discuss an eligibility of a subject to continue to be the study after Day 90±5.

12.6.4 Recommendations and Reporting

Recommendations for action are sent to the Principal Investigator. The Principal Investigator is responsible for reviewing and if necessary, implementing DSMC recommendations.

13 REFERENCES

1. Danese S, Fiocchi C. Ulcerative colitis. *N Engl J Med* 2011; 365(18):1713-25.
2. Dorofeyev, A. E., Vasilenko, I. V., Rassokhina, O. A., & Kondratiuk, R. B. (2013). Mucosal barrier in ulcerative colitis and Crohn's disease. *Gastroenterology research and practice* 2013;2013:431231.
3. Kappelman MD, Rifas-Shiman SL, Kleinman K, Ollendorf D, Bousvaros A, Grand RJ, Finkelstein JA. The prevalence and geographic distribution of Crohn's disease and ulcerative colitis in the United States. Clin Gastroenterol Hepatol. 2007; 5:1424-9.
4. Loftus EV, Jr. Clinical epidemiology of inflammatory bowel disease: Incidence, prevalence, and environmental influences. Gastroenterology. 2004; 126:1504-17.
5. Kinugasa T, Akagi Y. Status of colitis-associated cancer in ulcerative colitis. *World journal of gastrointestinal oncology*. 2016;8(4):351.
6. Yamamoto T, Hiroi A, Itagaki H, Kato Y, Iizuka B, Itabashi M, Shibata N, Nagashima Y. Well-differentiated adenocarcinoma associated with ulcerative colitis. *SAGE Open Medical Case Reports.* 2017;5:2050313X17692902.
7. Bernstein CN, Fried M, Krabshuis JH, Cohen H, Eliakim R, Fedail S, Gearry R, Goh KL, Hamid S, Khan AG, LeMair AW. World Gastroenterology Organization Practice Guidelines for the diagnosis and management of IBD in 2010. Inflammatory bowel diseases. 2010;16(1):112-24.
8. Ahmad T, Marshall S, Jewell D. Genotype-based phenotyping heralds a new taxonomy for inflammatory bowel disease. *Current opinion in gastroenterology*. 2003;19(4):327-35.
9. Cleynen I, Boucher G, Jostins L, Schumm LP, Zeissig S, Ahmad T, Andersen V, Andrews JM, Annese V, Brand S, Brant SR. Inherited determinants of Crohn's disease and ulcerative colitis phenotypes: a genetic association study. *The Lancet*. 2016;387(10014):156-67.
10. Kostic AD, Xavier RJ, Gevers D. The microbiome in inflammatory bowel disease: current status and the future ahead. Gastroenterology. 2014;146(6):1489-99.
11. Sartor RB. Microbial influences in inflammatory bowel disease. *Gastroenterology*. 2008;134(2):577-94.
12. Chu H, Khosravi A, Kusumawardhani IP, Kwon AH, Vasconcelos AC, Cunha LD, Mayer AE, Shen Y, Wu WL, Kambal A, Targan SR. Gene-microbiota interactions contribute to the pathogenesis of inflammatory bowel disease. *Science*. 2016;352(6289):1116-20.
13. Elahi M, Telkabadi M, Samadi V, Vakili H. Association of oral manifestations with ulcerative colitis. *Gastroenterol Hepatol Bed Bench*. 2012;5(3):155-60.
14. Xun Z, Zhang Q, Xu T, Chen N, Chen F. Dysbiosis and Ecotypes of the Salivary Microbiome Associated with Inflammatory Bowel Diseases and the Assistance in Diagnosis of Diseases Using Oral Bacterial Profiles. *Front Microbiol*. 2018;9:1136. doi:10.3389/fmicb.2018.01136

15. Pallone F, Blanco Gdel V, Vavassori P, Monteleone I, Fina D, Monteleone G. Genetic and pathogenetic insights into inflammatory bowel disease. *Curr Gastroenterol Rep*. 2003;5(6):487-92.
16. Heller F, Fuss IJ, Nieuwenhuis EE, Blumberg RS, Strober W. Oxazolone colitis, a Th2 colitis model resembling ulcerative colitis, is mediated by IL-13-producing NK-T cells. *Immunity*. 2002;17(5):629-38.
17. McCole, D. F. IBD Candidate Genes and Intestinal Barrier Regulation. *Inflammatory Bowel Diseases.* 2014;20(10), 1829–1849.
18. Merga Y, Campbell BJ, Rhodes JM. Mucosal barrier, bacteria and inflammatory bowel disease: possibilities for therapy. *Digestive diseases*. 2014;32(4):475-83.
19. Dorofeyev AE, Vasilenko IV, Rassokhina OA, Kondratiuk RB. Mucosal barrier in ulcerative colitis and Crohn’s disease. Gastroenterology research and practice. 2013;2013.
20. Alipour M, Zaidi D, Valcheva R, Jovel J, Martínez I, Sergi C, Walter J, Mason AL, Wong GK, Dieleman LA, Carroll MW. Mucosal barrier depletion and loss of bacterial diversity are primary abnormalities in paediatric ulcerative colitis. Journal of Crohn's and Colitis. 2015;10(4):462-71.
21. Gitter AH, Wullstein F, Fromm M, Schulzke JD. Epithelial barrier defects in ulcerative colitis: characterization and quantification by electrophysiological imaging. Gastroenterology. 2001;121(6):1320-8.
22. Johansson ME, Gustafsson JK, Holmén-Larsson J, Jabbar KS, Xia L, Xu H, Ghishan FK, Carvalho FA, Gewirtz AT, Sjövall H, Hansson GC. Bacteria penetrate the normally impenetrable inner colon mucus layer in both murine colitis models and patients with ulcerative colitis. Gut. 2013:gutjnl-2012.
23. Baron JA, Beach M, Mandel JS, van Stolk RU, Haile RW, et al. Calcium supplements for the prevention of colorectal adenomas. Calcium Polyp Prevention Study Group. *N Engl J Med* 1999;340: 101-107.
24. Wallace K, Baron JA, Cole BF, Sandler RS, Karagas MR, et al. Effect of calcium supplementation on the risk of large bowel polyps. *J Natl Cancer Inst* 2004;96: 921-925.
25. Grau MV, Baron JA, Sandler RS, Wallace K, Haile RW, et al. Prolonged effect of calcium supplementation on risk of colorectal adenomas in a randomized trial. *J Natl Cancer Inst* 2007;99: 129-136.
26. Keum NN, Aune D, Greenwood DC, Ju W, Giovannucci EL. Calcium intake and cancer risk: Dose-response meta-analysis of prospective observational studies. *Int J Cancer*. 2015; 136:1680-70.
27. Behrens J, Vakaet L, Friis R, Winterhager E, Van Roy F, Mareel MM, Birchmeier W. Loss of epithelial differentiation and gain of invasiveness correlates with tyrosine phosphorylation of the E-cadherin/beta-catenin complex in cells transformed with a temperature-sensitive v-SRC gene. *J Cell Biol*. 1993;120(3):757-66.
28. Hugh T.J., Dillion S.A., Taylor B.A., Pignatelli M., Poston G., Kinsella A.R. Cadherin-catenin expression in primary colorectal cancer: a survival analysis. *Brit. J. Cancer*. 1999;80, 1046-1051.

29. Mareel M., Berx G., Van Roy F., Bracke M. Cadherin/catenin complex: a target for antiinvasive therapy? *J. Cell Biochem*. 1996;61, 524-530.
30. Korinek V., Barker N., Morin P.J., van Wichen D., de Weger R., Kinzler K.W., Vogelstein B., Clevers H. Constitutive transcriptional activation by beta-catenin – TCF complex in APC-/- in colon carcinoma. *Science*. 1997;275, 1784-1787.
31. Gottardi C.J., Wong E., Gumbiner B.M. E-cadherin suppresses cellular transformation by inhibiting beta-catenin signaling in an adhesion-independent manner. *J. Cell Biol*. 2001;153, 1049-1059.
32. Van Aken E., De Wever O., Correia da Rocha A.S., Mareel M. Defective E-cadherin / beta-catenin complexes in human cancer. *Virchows Arch.* 2001;439, 725-751.
33. Conacci-Sorrell M., Simcha I., Ben-Yedidia T., Blechman J., Savagner P., Ben-Ze'ev. Autoregulation of E-cadherin expression by cadherin-cadherin interactions: the roles of beta-catenin signaling, Slug, and MAPK. *The J. Cell Biol.* 2003;163, 847-857.
34. Brembeck F.H., Schwarz-Romond T., Bakkers J., Wilhelm S., Hammerschmidt M., Birchmeier W. Essential role of BCL9-2 in the switch between beta-catenin's adhesive function and transcriptional functions. *Genes & Development*. 2004;18, 2225-2230.
35. Whitfield JF. Calcium, calcium-sensing receptor and colon cancer. *Caner Letters*. 2009;275(1):9-16.
36. Varani J. Calcium, calcium-sensing receptor and growth control in the colonic mucosa. *Histol. Histopathol*. 2011;26:769-799.
37. Rogers AC, Hanly AM, Collins D, Baird AW, Winter DC. Review article: Loss of the calcium sensing receptor in colonic epithelium is a key event in the pathogenesis of colon cancer. *Clin. Colorectal Cancer.* 2011;11:24-30.
38. Singh N, Aslam MN, Varani J, Chakrabarty S. Induction of calcium sensing receptor in human colon cancer cells by calcium, vitamin D and Aquamin: promotion of a more differentiated, less malignant and indolent phenotype. *Mol Carcinog*. 2015; 54(7):543-53.
39. Strain J.J., Cashman K.D. (2002). Minerals and trace elements. In: Introduction to Human Nutrition. Gibney MJ, Vorster HH, Kok FJ (eds.). Blackwell, pp 177-224.
40. Stipanuk M.H. (2006). Biochemical, Physiological, Molecular Aspects of Human Nutrition. 2nd ed. Elsevier, St. Louis, MO.
41. Harris E.D. Regulation of antioxidant enzymes. *FASEB J.* 1992;6(9), 2675-2683.
42. Huang Y., Zhou Y., Castiblanco A., Yang W., Brown E.M., Yang J.J. Multiple Ca2+ binding sites in the extracellular domain of the Ca2+ - sensing receptor corresponding to cooperative Ca2+ response. *Biochemistry* 2009;48, 388-398.
43. Attili D, Jenkins B, Aslam MN, Dame MK, Varani J. Growth control in colon epithelial cells: Gadolinium enhances calcium-mediated growth regulation. *Biological trace element research*. 2012;150(1-3):467-76.
44. Jenkins W, Perone P, Walker K, Bhagavathula N, Aslam MN, DaSilva M, Dame MK, Varani J. Fibroblast response to lanthanoid metal ion stimulation: potential

contribution to fibrotic tissue injury. *Biological trace element research*. 2011;144(1-3):621-35.

45. Adey WH and McKibbin DL: Studies on the maerl species Phymatolithon calcareum (Pallas) nov. comb. and Lithothamnium corallioides Crouan in the Ria de Vigo. *Botanical Marina* 13, 100–106, 1970.
46. Aslam M.N., Bhagavathula N., Chakrabarty S., Varani J. Growth-inhibitory effects of Aquamin, a mineralized extract from the red algae, Lithothamnion calcerum, on Ca^{2+} - sensitive and Ca^{2+} - resistant human colon carcinoma cells. *Cancer Letters*. 2009;283(2), 186-192.
47. Newmark H., Yang K., Kurihara N., Fan K., Augenlicht L., Lipkin M. Western-style diet-induced colonic tumors and their modulation by calcium and vitamin D in C57bl/6 mice: A preclinical model for human sporadic colon cancer. *Carcinogenesis*. 2009;30(1), 88-92.
48. Yang K., Kurihara N., Fan K., Newmark H., Rigas B., Bancroft L., Corner G., Livote E., Lesser M., Edelmann W., Velcich A., Lipkin M., Augenlicht L. Dietary induction of colonic tumors in a mouse model of sporadic colon cancer. *Cancer Res*. 2008;68, 7803-7810.
49. Aslam M.N., Paruchuri T., Bhagavathula N., Varani J. A mineralized extract from the red algae, Lithothamnion calcerum, inhibits polyp formation and inflammation in the gastrointestinal tract of normal mice on a high-fat diet. *Integrative Cancer Therapies*. 2010;9(1):93-99.
50. Aslam, MN, Ingrid Bergin I, Naik M, Paruchuri T, Hampton A, Rehman M, Dame MK, Rush H, Varani J. A Multimineral Natural Product from Red Marine Algae Reduces Colon Polyp Formation in C57BL/6 Mice. *Nutrition and Cancer*. 2012; 64(7), 1020–1028
51. Aslam MN, Bergin I, Naik M, Hampton A, Allen R, Kunkel SL, Rush H, Varani J. A multi-mineral natural product inhibits liver tumor formation in C57BL/6 mice. *Biol Trace Elem Res.* 2012;147(1-3):267-74.
52. Aslam MN, Paruchuri T, Bhagavathula N, DaSilva M, Goldstein SA, Varani J. A mineralized extract from the red algae, Lithothamnion calcerum, preserves bone structure and function in normal mice on a high-fat diet. *Calc. Tissue. International*. 2010;86(4):313-324.
53. Aslam MN, Bergin I, Jepsen K, Kreider JM, Graf KH, Naik M, Goldstein SA, Varani J. Preservation of bone structure and function by Lithothamnion sp. derived minerals. *Biol Trace Elem Res.* 2013;156(1-3):210-20.
54. Hampton AL, Aslam MN, Naik MK, Bergin IL, Allen RM, Craig RA, Kunkel SL, Veerapaneni I, Paruchuri T, Patterson KA, Rothman ED. Ulcerative dermatitis in C57BL/6NCrl mice on a low-fat or high-fat diet with or without a mineralized red-algae supplement. *Journal of the American Association for Laboratory Animal Science*. 2015;54(5):487-96.
55. Dame MK, Jiang Y, Appelman HD, Copley KD, McClintock SD, Aslam MN, Attili D, Elmunzer BJ, Brenner DE, Varani J, Turgeon DK: Human colonic crypts in

culture: Segregation of immunochemical markers in normal versus adenoma-derived. Lab. Invest. 94(2): 222-234, 2014

56. McClintock SD, Colacino JA, Attili D, Dame MK, Richter A, Reddy A, Basrur V, Rizvi AH, Turgeon DK, Varani J, Aslam MN: Calcium - Induced Differentiation of Human Colon Adenomas In Colonoid Culture: Calcium alone versus calcium with additional trace elements. *Cancer Prev Res* 11(7): 413-428, 2018.
57. McClintock SD, Rizvi AH, JA, Attili D, Pandya S, Rehman H, Nadeem DM, Dame MK, Turgeon DK, Varani J, Aslam MN. Response of Colonoid Cultures Derived from Histologically-Normal Human Colon Tissue to Calcium: Morphological Differentiation and Barrier Formation. 2018 (In Review)
58. Daig R, Andus T, Aschenbrenner E, Falk W, Scholmerich J, Gross V. Increased interleukin 8 expression in the colon mucosa of patients with inflammatory bowel disease. Gut 38:216-222, 1996.
59. Frestedt JL, Walsh M, Kuskowski MA, Zenk JL. A natural mineral supplement provides relief from knee osteoarthritis symptoms: a randomized controlled pilot trial. *Nutr. J.* 2008;7:9.
60. Frestedt JL, Kuskowski MA, Zenk JL. A natural seaweed derived mineral supplement (Aquamin F) for knee osteoarthritis: A randomized, placebo controlled pilot study. *Nutr. J.* 2009;8:7.
61. Aslam MN, Bassis C, Bergin I, Turgeon D, Varani J. Multi-Mineral Intervention to Modulate the Microbial Population of the Human Gut. In American Journal of Pathology 2018 Oct 1 (Vol. 188, No. 10, pp. 2427-2427).
62. Bernstein CN, Leslie WD, Leboff MS. AGA technical review on osteoporosis in gastrointestinal diseases. Gastroenterology 2003;124(3):795–841.
63. Cashman KD, Shanahan F. Is nutrition an aetiological factor for inflammatory bowel disease? Eur J Gastroenterol Hepatol 2003;15(6):607–613.
64. Aslam MN, Jepsen KJ, Khoury B, Graf KH, Varani J: Bone structure and function in male C57BL/6 mice: Effects of a high-fat Western-style diet with or without trace minerals. Bone Rep 5: 141-149, 2016.
65. Finley PR, Bogert CL, Alberts DS, Einspahr J, Earnest DL, Blackwell G, Girodias K. Measurement of prostaglandin E2 in rectal mucosa in human subjects: a method study. Cancer Epidemiol Biomarkers Prev.1995;4(3):239-244.

Appendices

1. **Table Certificate of analysis - Aquamin®**
2. **Table showing mineral composition of a typical batch of Aquamin®**
3. **Table of Adverse events seen in subjects taking Aquamin® in similar protocols**
4. **Flexible Sigmoidoscopy SOP**
5. **Tissue SOP**
6. **Blood Collection SOP**
7. **Saliva Collection SOP**
8. **Adherence Table**
9. **IND Clinical Trial Monitoring Plan**
10. **Data Safety and Monitoring Plan**

Appendix 1: Certificate of Analysis of Aquamin®

<rest of page intentionally left blank>

Marine Minerals for Health

Consignee:

Consigner: Marigot Ltd., Strand Farm, Currabinny, Co. Cork. Ireland

RE: AQUAMIN TG

PRODUCT:	**AQUAMIN TG**
BATCH:	**TG809188**
DATE OF MANUFACTURE:	**17TH September 2018**
SHELFLIFE:	**3 YEARS FROM DATE OF MANUFACTURE**
APPEARANCE:	**OFF WHITE FREE FLOWING GRANULATE**

Item	Specification	Result
**CALCIUM** (ICP)	30% Minimum	**30.6%**
**MAGNESIUM** (ICP)	2.2% Minimum	**2.3%**
**MOISTURE**	5% Maximum	**1.0%**
**T.V.C.**	10,000cfu/g Maximum	**132cfu/g**
**E.coli** in 1g	Absent	**Absent**
**Salmonella** in 25g	Absent	**Absent**
**Enterobacteriaceae in 1g**	Absent	**Absent**
**Coliforms in 1g**	Absent	**Absent**
**Staph.Aureus in 1g**	Absent	**Absent**
**Yeast & Moulds**	100cfu/g Maximum	**<20cfu/g**
**Arsenic:** (ICP)	1.5ppm Maximum	**0.8ppm**
**Lead** (ICP)	1.0ppm Maximum	**0.3ppm**
**Cadmium** (ICP)	1.0ppm Maximum	**0.6ppm**
**Mercury** (ICP)	0.1ppm Maximum	**<0.1ppm**
**Flow ability** (8 mm glass funnel)	8-11 seconds	**9.13seconds**
**Bulk density**	0.7-0.9g/cm³	**0.88g/cm³**
**Ash 450⁰C**	90% Minimum	**91.4%**

Signed:...
Angela Buckley Quality Manager 22nd October 2018

MARIGOT LTD, Strand Farm [illegible] | **TEL:** +353 21 4378727 | **WEB:** www.aquamin.org | **VAT NO:** [illegible]
[illegible] | **FAX:** +353 21 [illegible] | **EMAIL:** info@aquamin.org | **COMPANY REG. NO:** [illegible]

Appendix 2: Mineral composition of a typical batch of Aquamin®

(Four Pages)

<rest of page intentionally left blank>

40 West Louise Ave., Salt Lake City, UT 84115
Phone: (801) 485-1800 Fax: (801) 484-9211
Email: utlab@advancedlabsinc.com
FDA Registration #3006423386

If you liked our service, please tell a friend. If you didn't, please tell us!

Test Certificate

Description: Aquamin TG
Sample ID: 801009
Lot No:
Part Code:
Location:
PO No:
Received: 3/23/2018

Client: Marigot Limited
Strand Farm, Currabinny
Carrigaline, Co. Cork
Ireland

Lab No: 148472-01
Completed: 4/4/2018

Analysis	Result	Per Unit	Method
Carbon	124,000	ppm	ASTM D-1552
Aluminum	73.8	ppm	ICP-OES USP <730>
Antimony	<0.5	ppm	ICP-OES USP <730>
Arsenic	1.203	ppm	ICP-MS USP <730>
Barium	7.52	ppm	ICP-OES USP <730>
Beryllium	1.81	ppm	ICP-OES USP <730>
Bismuth	<0.5	ppm	ICP-OES USP <730>
Boron	34.2	ppm	ICP-OES USP <730>
Cadmium	0.650	ppm	ICP-MS USP <730>
Calcium	31.2	%	ICP-OES USP <730>
Cerium	0.762	ppm	ICP-MS USP <730>
Cesium	0.002	ppm	ICP-MS USP <730>
†Chloride	2,302	ppm	USP <221> Titration
Chromium	3.01	ppm	ICP-OES USP <730>
Cobalt	1.87	ppm	ICP-OES USP <730>
Copper	3.28	ppm	ICP-OES USP <730>
Dysprosium	0.143	ppm	ICP-MS USP <730>
Erbium	0.099	ppm	ICP-MS USP <730>
Europium	0.036	ppm	ICP-MS USP <730>

THESE RESULTS APPLY ONLY TO THE SAMPLE SUBMITTED AND NOT TO THE PRODUCT FROM WHICH IT WAS TAKEN. THESE RESULTS ARE PROVIDED ONLY FOR THE BENEFIT OF CLIENT, WITHOUT REPRESENTATION OR WARRANTY OF ANY KIND, EXCEPT FOR THE EXPRESS LIMITED WARRANTY PROVIDED SOLELY TO CLIENT IN ADVANCED LABORATORIES' TERMS OF SERVICE.

THIS CERTIFICATE SHALL NOT BE REPRODUCED EXCEPT IN FULL, WITHOUT WRITTEN APPROVAL FROM ADVANCED LABORATORIES.

Results Approved By: **Dated:** 4/4/2018

[illegible] Farnsworth-Quality Technician

Tests marked with † were done at Atlas Bioscience Labs, LLC, a joint venture with Advanced Laboratories. - 1775 S. Pantano Rd - Ste #110, Tucson, AZ 85710

Printed: 4/4/2018 3:38:43 PM

Page 1 of 4

40 West Louise Ave., Salt Lake City, UT 84115
Phone: (801) 485-1800 Fax: (801) 484-9211
Email: utlab@advancedlabsinc.com
FDA Registration #3006423386

If you liked our service, please tell a friend. If you didn't, please tell us!

Test Certificate

Description: Aquamin TG
Sample ID: 801009
Lot No:
Part Code:
Location:
PO No:
Received: 3/23/2018

Client: Marigot Limited
Strand Farm, Currabinny
Carrigaline, Co. Cork
Ireland

Lab No: 148472-01
Completed: 4/4/2018

Analysis	Result	Per Unit	Method
†Fluoride	3.492	ppm	AOAC 939.11
Gadolinium	0.136	ppm	ICP-MS USP <730>
Gallium	0.136	ppm	ICP-MS USP <730>
Germanium	<0.001	ppm	ICP-MS USP <730>
Gold	<0.5	ppm	ICP-OES USP <730>
Hafnium	0.113	ppm	ICP-MS USP <730>
Holmium	0.033	ppm	ICP-MS USP <730>
Indium	<0.001	ppm	ICP-MS USP <730>
†Iodine	0.103	ppm	Titration
Iridium	<0.001	ppm	ICP-MS USP <730>
Iron	609	ppm	ICP-OES USP <730>
Lanthanum	1.98	ppm	ICP-OES USP <730>
Lead	0.175	ppm	ICP-MS USP <730>
Lithium	<0.5	ppm	ICP-OES USP <730>
Lutetium	0.027	ppm	ICP-MS USP <730>
Magnesium	2.58	%	ICP-OES USP <730>
Manganese	43.2	ppm	ICP-OES USP <730>
Mercury	<0.001	ppm	ICP-MS USP <730>
Molybdenum	<0.5	ppm	ICP-OES USP <730>

THESE RESULTS APPLY ONLY TO THE SAMPLE SUBMITTED AND NOT TO THE PRODUCT FROM WHICH IT WAS TAKEN. THESE RESULTS ARE PROVIDED ONLY FOR THE BENEFIT OF CLIENT, WITHOUT REPRESENTATION OR WARRANTY OF ANY KIND, EXCEPT FOR THE EXPRESS LIMITED WARRANTY PROVIDED SOLELY TO CLIENT IN ADVANCED LABORATORIES' TERMS OF SERVICE.

THIS CERTIFICATE SHALL NOT BE REPRODUCED EXCEPT IN FULL, WITHOUT WRITTEN APPROVAL FROM ADVANCED LABORATORIES.

Results Approved By: **Dated:** 4/4/2018

[illegible] Farnsworth-Quality Technician

Tests marked with † were done at Atlas Bioscience Labs, LLC, a joint venture with Advanced Laboratories. - 1775 S. Pantano Rd - Ste #110, Tucson, AZ 85710

Printed: 4/4/2018 3:38:43 PM

Page 2 of 4

40 West Louise Ave., Salt Lake City, UT 84115
Phone: (801) 485-1800 Fax: (801) 484-9211
Email: utlab@advancedlabsinc.com
FDA Registration #3006423386

If you liked our service, please tell a friend. If you didn't, please tell us!

Test Certificate

Description:	Aquamin TG	Client:	Marigot Limited<br>Strand Farm, Currabinny<br>Carrigaline, Co. Cork<br>Ireland
Sample ID:	801009		
Lot No:			
Part Code:			
Location:			
PO No:		Lab No:	148472-01
Received:	3/23/2018	Completed:	4/4/2018

Analysis	Result	Per Unit	Method
Neodymium	0.456	ppm	ICP-MS USP <730>
Nickel	1.81	ppm	ICP-OES USP <730>
Niobium	12.0	ppm	ICP-OES USP <730>
Osmium	0.001	ppm	ICP-MS USP <730>
Palladium	0.464	ppm	ICP-MS USP <730>
Phosphorus	78.0	ppm	ICP-OES USP <730>
Platinum	<0.001	ppm	ICP-MS USP <730>
Potassium	200	ppm	ICP-OES USP <730>
Praseodymium	0.101	ppm	ICP-MS USP <730>
Rhenium	0.003	ppm	ICP-MS USP <730>
Rhodium	0.372	ppm	ICP-MS USP <730>
Rubidium	0.022	ppm	ICP-MS USP <730>
Ruthenium	2.044	ppm	ICP-MS USP <730>
Samarium	0.107	ppm	ICP-MS USP <730>
Scandium	1.733	ppm	ICP-MS USP <730>
Selenium	<0.5	ppm	ICP-OES USP <730>
Silicon	90.9	ppm	ICP-OES USP <730>
Silver	3.54	ppm	ICP-OES USP <730>
Sodium	4,886	ppm	ICP-OES USP <730>

THESE RESULTS APPLY ONLY TO THE SAMPLE SUBMITTED AND NOT TO THE PRODUCT FROM WHICH IT WAS TAKEN. THESE RESULTS ARE PROVIDED ONLY FOR THE BENEFIT OF CLIENT, WITHOUT REPRESENTATION OR WARRANTY OF ANY KIND, EXCEPT FOR THE EXPRESS LIMITED WARRANTY PROVIDED SOLELY TO CLIENT IN ADVANCED LABORATORIES' TERMS OF SERVICE.

THIS CERTIFICATE SHALL NOT BE REPRODUCED EXCEPT IN FULL, WITHOUT WRITTEN APPROVAL FROM ADVANCED LABORATORIES.

Results Approved By: Alisa Farnsworth-Quality Technician
Dated: 4/4/2018

Printed: 4/4/2018 3:38:43 PM

Page 3 of 4

40 West Louise Ave., Salt Lake City, UT 84115
Phone: (801) 485-1800 Fax: (801) 484-9211
Email: utlab@advancedlabsinc.com
FDA Registration #3006423386

If you liked our service, please tell a friend. If you didn't, please tell us!

Test Certificate

Description: Aquamin TG
Sample ID: 801009
Lot No:
Part Code:
Location:
PO No:
Received: 3/23/2018

Client: Marigot Limited
Strand Farm, Currabinny
Carrigaline, Co. Cork
Ireland

Lab No: 148472-01
Completed: 4/4/2018

Analysis	Result	Per Unit	Method
Strontium	2,353	ppm	ICP-OES USP <730>
Sulfur	3,223	ppm	ICP-OES USP <730>
Tantalum	0.009	ppm	ICP-MS USP <730>
Tellurium	<0.5	ppm	ICP-OES USP <730>
Terbium	0.023	ppm	ICP-MS USP <730>
Thallium	<0.5	ppm	ICP-OES USP <730>
Thorium	4.41	ppm	ICP-OES USP <730>
Thulium	0.015	ppm	ICP-MS USP <730>
Tin	0.228	ppm	ICP-MS USP <730>
Titanium	32.1	ppm	ICP-OES USP <730>
Tungsten	<0.5	ppm	ICP-OES USP <730>
Vanadium	3.66	ppm	ICP-OES USP <730>
Ytterbium	0.091	ppm	ICP-MS USP <730>
Yttrium	3.66	ppm	ICP-OES USP <730>
Zinc	3.06	ppm	ICP-OES USP <730>
Zirconium	5.35	ppm	ICP-OES USP <730>

THESE RESULTS APPLY ONLY TO THE SAMPLE SUBMITTED AND NOT TO THE PRODUCT FROM WHICH IT WAS TAKEN. THESE RESULTS ARE PROVIDED ONLY FOR THE BENEFIT OF CLIENT, WITHOUT REPRESENTATION OR WARRANTY OF ANY KIND, EXCEPT FOR THE EXPRESS LIMITED WARRANTY PROVIDED SOLELY TO CLIENT IN ADVANCED LABORATORIES' TERMS OF SERVICE.

THIS CERTIFICATE SHALL NOT BE REPRODUCED EXCEPT IN FULL, WITHOUT WRITTEN APPROVAL FROM ADVANCED LABORATORIES.

Results Approved By: **Dated:** 4/4/2018

Aleta Farnsworth-Quality Technician

Printed: 4/4/2018 3:38:43 PM

Page 4 of 4

-END of Mineral Composition Report.

Appendix 3: Adverse Events (AEs)

Adverse Events in a Similar Aquamin® Study

Events	Placebo	Aquamin®
Number of subjects treated	14	8
Total number of adverse events	21	10
Upper respiratory events	3	2
Gastrointestinal events	4	1
Food reaction	0	1
Hypertension / cardiovascular	1	1
Musculoskeletal events	4	3
Neurological events	2	0
Increased OA pain	7	2

See citation; Frestedt JL et al (59) for details.

Adverse Events in Aquamin® Pilot Study (HUM00076276)

Events	Placebo	Aquamin®
Number of subjects participated	10	10
Number of subjects reported events	3	3
Total number of adverse events	5	7
Gastrointestinal events	2	6
Upper respiratory events (Flu like symptoms)	1	0
Flu with fever (Upper respiratory)	1	0
Skin rash	0	1
Headache	1	0

*This table shows AEs from the ongoing trial at University of Michigan conducted under IND118194. That trial is ongoing but all subject intervention is complete. There were no serious adverse events reported.

Serum Biomarkers in Aquamin® Pilot Study (HUM00076276)

Group	Total protein (6.0-8.3 g/dl)	Albumin (3.5-4.9 g/dl)	AST (8-30 IU/L)	ALT (<=35 IU/L)	ALKP (30-116IU/L)	Bilirubin (0.2-1.2 mg/dl)
**Placebo V2**	**7.2 ± 0.4**	**4.4 ± 0.2**	**25.6 ± 3.7**	**28.7 ± 8.3**	**75.8 ± 9.0**	**0.46 ± 0.2**
**Placebo V3**	**7.3 ± 0.3**	**4.5 ± 0.2**	**26.3 ± 5.5**	**31.9 ± 7.1**	**77.9 ± 15.3**	**0.54 ± 0.2**
**Calcium V2**	**7.1 ± 0.7**	**4.4 ± 0.4**	**28.1 ± 10.1**	**25.9 ± 12.3**	**78.1 ± 20.5**	**0.59 ± 0.6**
**Calcium V3**	**7.2 ± 0.6**	**4.5 ± 0.4**	**28.7 ± 9.6**	**28.8 ± 11.5**	**80.5 ± 21.0**	**0.60± 0.2**
**Aquamin® V2**	**7.1 ± 0.4**	**4.5 ± 0.1**	**24.8 ± 6.8**	**23.4 ± 11.2**	**73.7 ± 18.4**	**0.57 ± 0.2**
**Aquamin® V3**	**7.3 ± 0.4**	**4.6 ± 0.2**	**30.0 ± 10.9**	**32.5 ± 19.6**	**74.4 ± 19.7**	**0.61 ± 0.4**

No significant differences.

Appendix 4: Detailed procedures for Unprepped Flexible Sigmoidoscopy

1) Subject Preparation for Endoscopy Procedures
Sigmoidoscopies for mucosal sampling will be performed on an unprepped colon. The sigmoidoscope will be prepared for standard endoscopy unit procedures including preparation of suction tubing valves and light source. All the necessary supplies will be arranged for easy access. The subjects are provided with a hospital gown in the MCRU procedure endoscopy room. The physician explains the procedure in detail and obtains appropriate consent. The subject is placed in a left lateral decubitus position; sigmoidoscope is lubricated and introduced by the physician. The entire rectum and sigmoid is inspected.

2) Sampling of Fecal Material
At the beginning of the flexible sigmoidoscopy, biopsy forceps, designed for sampling in the colon, will be inserted through the scope. The biopsy forceps will be extended into the remaining stool. The forceps will be withdrawn into the sheath, and removed from the endoscope. The CRA will scoop out the fecal specimen using a sterile needle into a cryovial. Biopsy forceps will be used to collected fecal material in a cryovial for fecal calprotectin, microbial and metabolomics analysis. These fecal samples will be snap frozen for microbial and metabolomics analysis except samples for calprotectin which will be stored at 2-8°C.

3) Sampling of Normal Appearing Colonic Mucosa (Biopsy and Post-Biopsy Procedures):
A total of Ten samples biopsies will be obtained. Tissue samples are usually taken from the distal sigmoid colon (15-25 cm from the anus). The clean new biopsy forceps are positioned perpendicular to the mucosal surface opened completely and pressed against the mucosal surface with mild pressure to be sure that the forceps jaws are completely filled. The forceps jaws are then closed and the forceps removed. A cuff of edema will develop in the mucosa immediately adjacent to the biopsy site. Sequential biopsies are taken around the sigmoid wall avoiding the edematous region. After the biopsy procedure is complete, the biopsy sites are inspected for bleeding and the endoscope is removed and cleaned. The subject is assisted off the table, escorted to the holding area and allowed to dress. After 10-20 min of observation, the subject is permitted to leave. The subject is counseled for any side effects, given telephone numbers to obtain assistance, and provided with reminders for next contacts.

4) Procedure Morbidity
Bleeding is generally minimal and usually stops spontaneously. The subjects will be advised to return in the case of any evidence of major rectal bleeding. Discomfort is mainly in the form of pressure during the procedure. There is no sensation during the biopsy. No sedation is used. Infection of the biopsy site is essentially unknown after colonic biopsy in clinical practice. Perforation is highly unlikely since the biopsies are less than full thickness.

Appendix 5: Tissue/Stool SOP

Gloves must be worn at all times when handling tissue specimens and supplies. This includes during removal of the tissue biopsy from the biopsy forceps, putting the cap on the vial and disposal of contaminated tubes or needles. Tissue, tubes, needles, and pipets must be properly disposed of in biohazard containers, in accordance with institutional requirements.

1) Colon Biopsy / Stool Samples Collection Supplies
   - Timer
   - Normal Saline to wash tissue (4°C) in shallow dish
   - Gauze to blot tissue after rinsing
   - 18 ga blunt needles
   - Biopsy forceps
   - 6 pre-labeled 2 ml cryogenic tubes
   - 1 pre-labeled tube with 10% Formalin
   - 1 pre-labeled 5ml tube with transport medium (colon tissue for culture; baseline visit only)
   - 4 pre-labeled 2 ml cryogenic tubes (stool)
   - 1 pre-labeled 2 ml tube (Fecal Calprotectin- stool)
   - 1 pre-labeled 2 ml tube with anaerobic transport medium (for microbial cultivation- stool)
   - Liquid Nitrogen or Dry ice in a safe transport container
   - Styrofoam container with ice

2) Colon Tissue Processing

Frozen Biopsies, no solvent

1. Place biopsy into saline directly or use 18g needles if needed to scoot tissue off forceps.
2. Do not use forceps, tweezers or other tools that could crush the tissue.
3. Briefly, rinse tissue piece with cold saline to remove blood, or stool, blot on gauze using blunt 18 ga needles and transfer to corresponding tube.
4. Drop the closed cryogenic vial into liquid nitrogen or onto dry ice exactly 20 seconds after removal of the biopsy from subject.
5. Store at -70 or -80° C

Biopsies on ice, transport medium (for ex vivo culture at baseline visit)

1. Place biopsy into saline directly or use 18g needles if needed to scoot tissue off forceps.
2. Do not use forceps, tweezers or other tools that could crush the tissue.
3. Briefly, rinse tissue piece with cold saline to remove blood, or stool, blot on gauze using blunt 18 ga needles and transfer to corresponding tube, pre-filled with transport culture medium.

4. Place the closed 5 ml tube onto ice in a Styrofoam container as soon as possible after removal of the biopsy from subject.
5. Store at -70 or -80° C for long-term cryopreservation (banked away) if not put in a colonoid culture plate the same day.

Fixed Biopsies

1. Take biopsies off of forceps by swishing into a vial containing 10% formalin-90% PBS pH 7.4. Rinse forceps in saline between passes.
2. Store and transport to the lab
3. Within 45 minutes, orient biopsies on a piece of lens paper under dissecting microscope. Put mucosal side up and avoid touching mucosal side. Stretch out biopsy with mucosa side up.
4. Make a dye mark with a thin, pointed wooden applicator on the left side of the tissue.
5. Put in slide cassette, use pencil (only pencil) to label cassette.
6. Place the cassette into 10% formalin-90% PBS pH 7.4.
7. Keep in 10% formalin-90% PBS pH 7.4 for 18-24 hours between 20 and 25°C. DO NOT exceed 24 hours.
8. Transfer the cassette to 70% ethanol at room temperature.
9. Take to Tissue Processing Core within 1 week for paraffin embedding.
10. One biopsy: store paraffin block. Other biopsy: make eight 4 µm slides: 1 for each, Ki67, E-cadherin, CK20, occludin, cadherin-17, claudin-23 and desmoglein-2. Mark length of biopsy on histology form.

3) Stool Sample Processing

Frozen Samples, no solvent

1. Use 18g needles to scoot stool samples off forceps.
2. Transfer the sample to corresponding tube using the 18g needle.
3. Drop the closed cryogenic vial into liquid nitrogen or onto dry ice exactly 20 seconds after removal of the biopsy from subject.
4. Store at -70 or -80° C

Samples on ice, transport medium

5. Use 18g needles to scoot stool samples off forceps.
6. Transfer the sample using an 18g needle to corresponding tube pre-filled with transport culture medium (either anaerobic medium for microbiome and no medium for fecal calprotectin)
7. Place the closed tubes onto ice in a Styrofoam container as soon as possible after removal of the biopsy from subject.
8. Store at -20° C for long-term storage after processing with the extraction buffer (fecal calprotectin) and cryopreservation if not put in a culture plate the same day. Store the second tube at -70 or -80° C for microbiome cultivation, later in one batch.

Vials and Labels

Cryogenic Vial #1 Stool Sample 1 (Pass # 1)
Label – Microbiome Stool #1
Solvent – None
Storage: Snap Frozen in liquid nitrogen

Cryogenic Vial #2 Stool Sample 2 (Pass # 2)
Label – Metabolomics Stool #1
Solvent – None
Storage: Snap Frozen in liquid nitrogen

Cryogenic Vial #3 Stool Sample 3 (Pass # 3)
Label – Metabolomics Stool #2
Solvent – None
Storage: Snap Frozen in liquid nitrogen

Cryogenic Vial #4 Stool Sample 4 (Pass # 4)
Label – GF Study
Solvent – None
Storage: Snap Frozen in liquid nitrogen

Cryogenic Vial #5 Stool Sample 5,6 (Pass # 5, 6)
Label – Fecal Calprotectin (2 samples)
Solvent – None
Storage: Place on ice (at 2-8° C) during transportation

Cryogenic Vial #6 Stool Sample 7 (Pass # 7)
Label – Microbiome Stool #2
Solvent – Anaerobic transport medium (for microbial cultivation- stool)
Storage: Transport on ice during transportation

Cryogenic Vial #7 Normal colonic mucosa with cold forceps (Pass #8) One biopsy per pass. One biopsy in one vial.
Label – Microbiome # 1
Solvent – None
Storage –1 bx/vial, Snap Frozen in liquid nitrogen

Cryogenic Vial #8 Normal colonic mucosa with cold forceps (Pass #9) One biopsy per pass. One biopsy in one vial.
Label – Microbiome # 2
Solvent – None
Storage –1 bx/vial, Snap Frozen in liquid nitrogen

Cryogenic Vial #9 Normal colonic mucosa with cold forceps (Pass #10).
Label – Metabolomics # 1

Solvent – None
Storage –1 bx/vial, Snap Frozen in liquid nitrogen

Cryogenic Vial #10 Normal colonic mucosa with cold forceps (Pass #11).
Label – Metabolomics #2
Solvent – None
Storage –1 bx/vial, Snap Frozen in liquid nitrogen

Cryogenic Vial #11 Normal colonic mucosa with cold forceps (Pass #12).
Label – IL-8 (Cytokines)
Solvent – None
Storage –1 bx/vial, Snap Frozen in liquid nitrogen

Cryogenic Vial #12 Normal colonic mucosa with cold forceps (Pass #13,14).
Label – Proteomics (2bx)
Solvent – None
Storage –2 bx/vial, Snap Frozen in liquid nitrogen

Vial #13 Normal colonic mucosa with cold forceps for three biopsies (Pass #15-17).
Label – H&E/IHC (3bx)
Assays: Histological assessment by a pathologist for assessment and presence of any inflammatory cells in H&E sections. Immunohistochemistry (IHC) staining for proliferation, barrier and others markers.
Solvent – Taken to lab in 10% formalin-90% PBS
Storage – 3 bx in 1 vial. Fixation in formalin, transfer to individual block containing one biopsy per block lined up in lengthwise position.

Vial #14 Normal colonic mucosa with cold forceps for two biopsies (Pass #18,19) (Baseline visit only)
Label – Colonoid Culture (2 bx)
Assays: Proliferation and Differentiation Assays in culture
Solvent – (cold Dulbecco's minimal essential supplemented with Glutamax and antibiotics as a transport medium)
Storage – 2 bx in 1 vial. Transfer to lab on ice to start a 3D culture or bank away.

Appendix 6: Blood SOP

Gloves must be worn at all times when handling specimens and supplies. This includes during removal of the rubber stopper from the blood tubes, centrifugation, pipetting, disposal of contaminated tubes, and cleanup of any spills. Tubes, needles, and pipets must be properly disposed of in biohazard containers, in accordance with institutional requirements.

Blood Kit and Associated Supplies

Small, biohazard plastic bags that contain:

- One 10 ml red top glass (serum) Vacutainer® tube
- SST tube for clinical labs (MCRU)
- SID Labels for labeling Vacutainer® tubes

Handling Red Top Vacutainer® Tube (Serum) (To Varani Lab)

UPRIGHT IN A RACK COVERED WITH FOIL (in the dark)

Room temperature for a minimum of 30 to a maximum of 60 minutes to allow the clot to form.
4°C: Maximum of 4 hours, No minimum at 4°C required. Transfer to Varani lab on ice (in a Styrofoam box).

After the spin, stored serum at -20°C after labeling it “for serum bile acids and bone markers”.

SST Tube for ALT, AST and ALKP (as part of Comprehensive Metabolic Panel) and CRP

- Collect 1 3ml SST tube
- Place MiChart order for COMP panel (including ALT, AST and ALKP test)
- Apply label with reg number, name, DOB
- Deliver to Central Distribution for test

APPENDIX 7: SALIVA SOP

Gloves must be worn at all times when handling salivary specimens and supplies. This includes during removal of the cap from the tubes, pipetting, disposal of contaminated tubes, and cleanup of any spills. Tubes, and pipets must be properly disposed of in biohazard containers, in accordance with institutional requirements.

- Subjects will provide saliva samples in MCRU by scraping of the oral (buccal and gingival) mucosa using a swab on a plastic shaft and storing the swab in a pre-labeled tube with subject/specimen ID.
- Salivary sample will be kept cold by the use of ice packs while in transport to Varani lab.
- Once in the lab Saliva tube will be stored at -80°C

Appendix 8: Assessment of Adherence with Agent

Number of capsules/Day	75% of daily dose =adherent	Number of Days	75% adherent with dosing period (days)
4	3	14	10
		21	16
		28	21
		35	26
		42	32
		49	37
		56	42
		63	47
		70	53
		77	58
		84	63
		90	68
		104	78
		118	89
		132	99
		146	110
		160	120
		174	131
		180	135

Appendix 9: IND Clinical Trial Monitoring Plan

Proper monitoring ensures adequate protection of the rights of human subjects, the safety of subjects involved in a clinical investigation and the quality and integrity of the data submitted as a result of the investigation. Therefore, ongoing monitoring of this clinical research investigation will be conducted with the intent to:

1. Verify patient consent for study participation has been properly obtained and documented ensuring compliance with Good Clinical Practice (GCP) standards and regulations for protection of human subjects.
2. Verify research patients entered into the study meet inclusion and exclusion criteria.
3. Verify the study is conducted in compliance with the protocol.
4. Verify the accuracy of the data collected.
5. Verify all essential documentation required by GCP standards and Investigational New Drug (IND) regulations are present, current and appropriately filed.

Routine monitoring will be scheduled at appropriate intervals, with more frequent visits occurring at the beginning of the study.
A study initiation meeting will take place prior to starting the study. As part of this meeting, the PI, co-Is, and study staff will review the protocol in detail, review the SOPs, undergo training on all the equipment, review logistics, and ensure that the study team understands their role.
For each subject enrolled, there will be 100% monitoring of informed consent documents, inclusion/exclusion criteria, serious adverse events, and unanticipated problems. In addition, the first eight subjects enrolled will have 100% of the data collection forms reviewed to ensure that all data collection has been completed and to ensure compliance with the protocol.

Consent forms:
1. Review of the consent. The patient must sign the IRB-approved version of the informed consent prior to initiation of the protocol-directed intervention.
    a. Verify that a signed and dated informed consent was obtained from every patient screened and that the principal investigator or designee has signed all consents.
    b. Verify the date that the informed consent was signed is before any study-related activities were performed.
    c. Verify that the correct version of the informed consent was signed initially and that any subsequent versions of the informed consent which were approved during the time the patient participated in the study have also been signed.

Inclusion/Exclusion Criteria:
1. All inclusion/exclusion criteria must be met to determine eligibility to enter the trial.
    a. Verify that the principal investigator has signed the eligibility form confirming patient eligibility.
    b. Verify that there are no contraindications to the planned procedures.
    c. Verify that a pregnancy test is negative.

Protocol compliance:

1. Verify that the study is being conducted in compliance with the current IRB/FDA approved protocol.

SAE reporting:

1. Serious adverse events must be reported according to protocol requirements.
   a. Verify that the serious adverse event was reported within the proper time period.
   b. Verify that a description of the SAE, interventions, and outcome are described in the progress note and have been provided/faxed to the Project Manager.
   c. Verify that a copy of the SAE report and IRB notification are filed in the regulatory binder.
   d. Verify that a SAE report was sent to the FDA and is filed in the regulatory binder

Unanticipated Problems reporting:

1. Unanticipated problems must be reported according to protocol, FDA and IRB requirements.
   a. Verify that an evaluation of the unanticipated problem was completed and that risk determination to subjects has been made.
   b. Verify that the unanticipated problem report was sent to the FDA and IRB and that copies of such correspondence are filed in the regulatory binder.
   c. Verify that a copy of the Unanticipated Problem report, IRB notification, IRB and FDA response are filed in the regulatory binder.

Follow up:

1. Follow up visits must be completed according to the schedule outlined in the protocol.
   a. Verify that all visits took place.
   b. Verify that all follow-up visits took place, including adverse event reporting.
   c. Verify that if any visits have been missed that the site has documented their attempts to contact the patient.

Appendix 10: Data Safety and Monitoring Plan

Data Safety Monitoring Committee

1.1 Definitions

- Data and Safety Monitoring Committee (DSMC)
- Data and Safety Monitoring Report (DSMR)
- Data and Safety Monitoring Plan (DSMP)
- Serious Adverse Events (SAE)*
- Unanticipated Problems (UaP)*
- Adverse Events (AE)*
- Principal Investigator (PI)
- Institutional Review Boards of the University of Michigan Medical School (IRBMED)
- National Institutes of Health (NIH)
- National Cancer Institute (NCI)
- Food and Drug Administration (FDA)
- Investigational New Drug (IND)
- Good Clinical Practice (GCP)
- Early Detection Research Network (EDRN)

1.2 Scope

The DSMC will review those projects in cancer prevention/biomarker development whose PIs wish to include their protocols in our DSMP. The primary focus of this group is to review early detection and prevention-based clinical trials as well as biorepository protocols. The DSMC may accept additional projects for review at its discretion as long as another review mechanism is not available and the research itself does not involve a treatment or intervention that is outside the expertise of at least 3 members of the DSMC.

1.3 Authority

The DSMC reviews, makes recommendations, and acts on the following:

1. All protocols without external oversight will be monitored by the DSMC.

2. Progress towards completion of the trial—recruitment and retention of study subjects.

3. Insufficient accrual to warrant continuation of the trial.

4. Evaluation of interim data analyses.

5. Evaluation of interim new information.

6. Evaluation of toxicity events including reporting of adverse events.

7. Timeliness of data.

8. Quality of data.

9. Ethical conduct of research.

The DSMC is empowered with the authority to recommend a trial be suspended or terminated based upon concerns in any of the above areas of review. The DSMC reviews all adverse events and ensures that these events have been correctly reported to all institutional review boards, and that adverse events have been correctly classified as serious or not serious. The DSMC assesses the impact of these events upon the conduct of the clinical trial. The DSMC is empowered with the authority to suspend or terminate any trials for which there are concerns of toxicity that endanger human subjects. Monitoring also considers factors external to the study, such as scientific or therapeutic developments that may have an impact on the safety of the subjects or the ethics of the study. Recommendations that emanate from monitoring activities are reviewed by the principal investigator and addressed.

1.4 Composition

The principal investigator is present for the DSMC unless asked to step out because multiple studies under multiple PIs are reviewed at one meeting. All DSMC members participating in the review of confidential data and discussions regarding continuance or stoppage of a study have no conflict of interest and no financial stake in the research outcome. Any member of the DSMC,

concerned about conflict of interest, can request that the chair ask the investigator to recuse themselves from deliberations and/or voting. The current UM Prevention research base Data and Safety Monitoring Committee is Chaired by the Dr. Mack Ruffin, MD, MPH. DSMC members include: Dr. Kim Turgeon, M.D., Dr. Michelle Anderson, M.D., MPH, and Elena Stoffel, M.D, MPH, Gastroenterology; Dr. Mack Ruffin, M.D., MPH, and Dr. Suzanna Zick, N.D. Family Medicine, Dr. Dean Brenner, M.D., Dr. Chris Lao, M.D. M.P.H.; and Dr. Ananda Sen, Biostatistics. At least 3 faculty members must be present to have a quorum. If the DSMC cannot meet face-to-face, a conference call is acceptable.

1.5 Meeting Frequency

The UM Prevention Research Base DSMC meets monthly by means of regularly scheduled meetings. If quorum cannot be achieved at a face-to-face meeting, then a conference call is an acceptable substitute meeting. Prior to each meeting, the clinical research associate distributes a standard summary report detailing accrual, biomarker modulations data, new publications or presentations relevant to the ongoing project, quality control audit information, any ethical concerns, patient-subject complaints, and adverse events or serious adverse events of all prevention protocols.

1.6 Recommendations and Reporting

Recommendations for action are sent to the Principal Investigator. The Principal Investigator is responsible for implementing DSMC recommendations. In addition to the Principal Investigator, minutes from the monthly meetings are available to the following as needed:

1. DSMC members and the principal investigators at other sites
2. The University of Michigan Comprehensive Cancer Center Prevention and Control Protocol Review Committee Chair
3. IRBMED (University of Michigan Medical School IRB);
4. NCI/DCP Program Staff;
5. Any other trial sponsor.

Serious adverse events and adverse events are reported to the institutional review boards of all clinical sites, sponsors, and University of Michigan IRBMED as specified in the protocol. If not SAE/AE reporting is not specified in the protocol, then standard SAE reporting guidelines for the IRBMed apply. The UM prevention data management office has the responsibility of informing other trial investigators concerning the data and safety monitoring policy, procedures, and decisions.

Adverse Event Definitions and Reporting

Serious adverse event classification for prevention studies

1. Mild
2. Moderate
3. Serious/Severe
4. Life-threatening
5. Death
6. Related/unrelated to the research
7. Expected per disease or research/unexpected

Adapted SAE reporting from IRBMED Standard (related to the research, not the underlying disease or death from cancer for example).

Regulatory binder:

1. The regulatory binder will be checked at each visit to ensure that all required regulatory documents have been obtained prior to the start of the study and have been kept current during the study. They include:
    a. Protocol and protocol amendments
    b. Sample, approved Informed Consent form
    c. CVs for investigators and sub-investigators
    d. Copies of current licenses and or certifications
    e. Sample CRFs
    f. Subject master log/Subject screening log
        i. Must show documentation of existence/participation of subjects
        ii. Must be consistent with information within the CRF and in the individual subject record.
    g. Serious Adverse Event log
    h. Drug Reconciliation records
    i. IRB approval
    j. IRB composition
    k. IRB correspondence
    l. FDA IND approval
    m. Signature log
    n. Delegation of Authority log
    o. Visitor log
        i. Monitor will sign and date the log indicating:
        ii. Date of the visit
        iii. Name of the individual conducting visit
        iv. Coordinator will confirm that visit took place by initialing.
    n. Correspondence related to the study

Source documents:
Source documents are essential documents that individually and collectively permit evaluation of the conduct of a clinical study and the quality of the data produced. Examples of source documents are hospital records, office charts, lab reports, x-rays, case report forms when date is entered directly, magnetic media, and photographic negatives.
Specific case report forms in this trial will be considered source documents in and of themselves because the data are collected one on one with the subject and entered directly onto the case report form.
